# Structural insights into the pSer/pThr dependent regulation of the SHP2 tyrosine phosphatase in insulin and CD28 signaling

**DOI:** 10.1038/s41467-022-32918-5

**Published:** 2022-09-16

**Authors:** András Zeke, Tamás Takács, Péter Sok, Krisztina Németh, Klára Kirsch, Péter Egri, Ádám Levente Póti, Isabel Bento, Gábor E. Tusnády, Attila Reményi

**Affiliations:** 1 Membrane Protein Bioinformatics Research Group, Institute of Enzymology, Research Centre for Natural Sciences, H-1117 Budapest, Hungary; 2 Biomolecular Interactions Research Group, Institute of Organic Chemistry, Research Centre for Natural Sciences, H-1117 Budapest, Hungary; 3 Signal Transduction and Functional Genomics Research Group, Insitute of Enzymology, Research Centre for Natural Sciences, H-1117 Budapest, Hungary; 4 Doctoral School of Biology, Institute of Biology, ELTE Eötvös Loránd University, H-1117 Pázmány Péter sétány 1/C, Budapest, Hungary; 5 Chemical Biology Research Group, Institute of Organic Chemistry, Research Centre for Natural Sciences, H-1117 Budapest, Hungary; 6 EMBL Outstation Hamburg, c/o DESY, Notkestr. 85, 22607 Hamburg, Germany

**Keywords:** Hydrolases, Phosphorylation, Molecular modelling, X-ray crystallography

## Abstract

Serine/threonine phosphorylation of insulin receptor substrate (IRS) proteins is well known to modulate insulin signaling. However, the molecular details of this process have mostly been elusive. While exploring the role of phosphoserines, we have detected a direct link between Tyr-flanking Ser/Thr phosphorylation sites and regulation of specific phosphotyrosine phosphatases. Here we present a concise structural study on how the activity of SHP2 phosphatase is controlled by an asymmetric, dual phosphorylation of its substrates. The structure of SHP2 has been determined with three different substrate peptides, unveiling the versatile and highly dynamic nature of substrate recruitment. What is more, the relatively stable pre-catalytic state of SHP2 could potentially be useful for inhibitor design. Our findings not only show an unusual dependence of SHP2 catalytic activity on Ser/Thr phosphorylation sites in IRS1 and CD28, but also suggest a negative regulatory mechanism that may also apply to other tyrosine kinase pathways as well.

## Abstract (web summary)

SHP2 is an important human tyrosine phosphatase with key roles in cancer, immune responses and insulin signaling. Here, the authors explore its substrate recognition mechanism in molecular detail and uncover a complex regulatory mechanism for this enzyme that marks specific target sites for dephosphorylation.

## Introduction

**Fig. 1:**
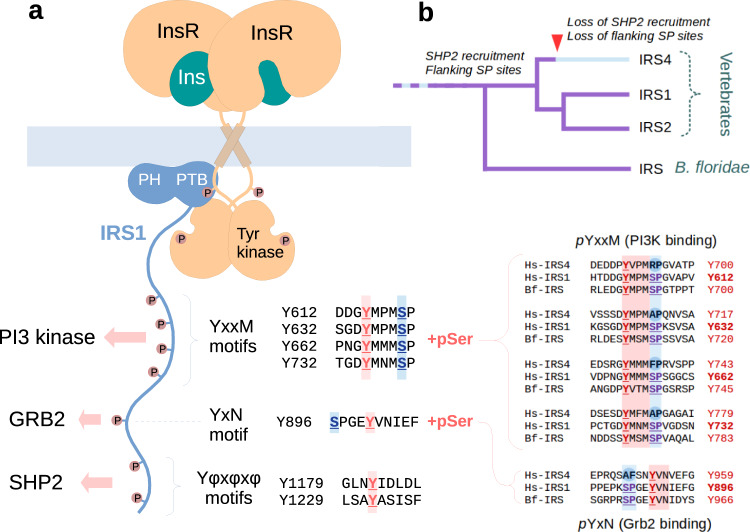
Overview of insulin signaling and Tyr-flanking Ser-Pro sites. **a** A brief overview of the insulin receptor complex with insulin receptor substrates. Specific phosphorylation sites of IRS1 are responsible for recruiting specialized effectors of signaling, such as PI3K, GRB2, and SHP2. Many of these pTyr sites are consistently flanked by phosphorylatable Ser-Pro sites in both IRS1 and IRS2 proteins. **b** Evolutionary analyses showing that flanking serine phosphorylation sites have co-evolved with SHP2 recruitment and were concomitantly lost on the vertebrate IRS4 lineage. Conservation of all five sites in the single *Branchiostoma floridae* (stem chordate) IRS ortholog (UniProt: C3ZU02) clearly shows that their loss in IRS4 is secondary.

Insulin signaling has been studied extensively in the past decades, in an attempt to better understand the pathomechanism of diabetes, one of the greatest medical burdens in the developed world. Insulin resistance is a pathological state of insulin receptor pathway desensitization that usually precedes and eventually leads to type II diabetes. Insulin receptor substrates (IRS proteins) are conserved mediators of insulin tyrosine kinase receptor (InsR) action, intimately involved in the development of insulin resistance^1^. IRS proteins are directly phosphorylated by InsR on tyrosine residues located in multiple YxxM motifs, which are evolutionarily conserved in all IRS paralogs (IRS1-4). These phosphorylation events lead to the membrane recruitment of phosphatidyl-inositol-3-kinase (PI3K) that mediates most cellular effects of insulin stimulation. Other tyrosine-containing IRS motifs are also phosphorylated (such as a GRB2-binding YxN site, or two dedicated SHP2 phosphatase recruitment sites), further contributing to the insulin signaling pathway. Interestingly, these tyrosine phosphorylation sites are frequently preceded or followed by conserved serine phosphorylation sites (see Fig. 1a). While IRS1 and IRS2 play a central, yet redundant role in this pathway, the role of the divergent IRS4 is less clear^2^.

In the past, several mechanisms were proposed that could explain decreased insulin signaling in target tissues^3^. One of the best studied phenomena was serine phosphorylation of IRS1/2 proteins, but the roles and molecular mechanisms underlying these serine modification events have remained unclear^1,4^. For example, we do not know how serine phosphorylation at sites adjacent to YxxM motifs contribute to insulin pathway desensitization. However, recent studies have highlighted a possible link between the phosphorylation of serine amino acids located immediately after this motif (YxxMSP sites) and IRS1 endocytosis^5^. What is more, it has been suggested that inhibition of SHP2—a tyrosine phosphatase capable of dephosphorylating these tyrosines—might ameliorate insulin resistance in cellular as well as in animal models^5^. While SHP2 is indeed recruited and activated by the insulin signaling pathway, its role has been so far poorly understood^6^. Therefore, it has been our principal goal to clarify these biochemical connections at a molecular or even atomic level.

SHP2 tyrosine phosphatase (SH2 domain containing phosphatase 2, also known as PTPN11) has recently emerged as a key player in cell proliferation and immunity, providing an attractive therapeutic target for cancer. SHP2 activity is regulated by autoinhibition, unlocked by recruitment to specific pTyr-containing motif pairs^7^. Another unusual feature of SHP2 is that it is a very picky enzyme, dephosphorylating only relatively few sites in select substrates^8^. Specific dephosphorylation of substrates like Src kinases or Ras-family small G-proteins can now clearly explain why SHP2 activating mutations are oncogenic^9^. Mutations in the PTPN11 (SHP2) gene are frequent causative agents of juvenile myelomonocytic leukaemia (JMML) through Ras hyperactivation^10^. However, effects of SHP2 activation are pleiotropic as it is a key modulator of multiple phosphotyrosine systems, such as the Met receptor^11^. Evolutionary comparisons suggest that SHP2 is also an ancient component of the insulin signaling pathway in all multicellular animals. Interestingly, this partnership is conserved in vertebrate IRS1 and IRS2 paralogs only, but not in the lineage leading up to IRS4. The latter protein also seems to have lost all the five serine phosphorylation sites that flank most of the conserved YxxM and YxN motifs, even in primitive, non-vertebrate chordates. The co-evolution of these two distinct features provides us the first hint that they might be tightly connected in their function (Fig. 1b).

In our current study, we shall present data suggesting that SHP2 is indeed a very special negative regulator of insulin receptor pathway: We show that IRS1/2 tyrosine dephosphorylation by SHP2 is enabled by specific serine phosphorylation events in the former proteins. In addition, we suggest that SHP2 has the capacity to negatively influence other phosphotyrosine-based systems (e.g CD28) as well. The mechanistic basis of these connections is provided by the versatile binding of SHP2 to dually phosphorylated substrate peptides, modified at specific positions.

## Results

### Binding and catalytic activity of SHP2 on IRS1 YxxMSP phosphopeptides is strongly dependent on serine phosphorylation

**Fig. 2:**
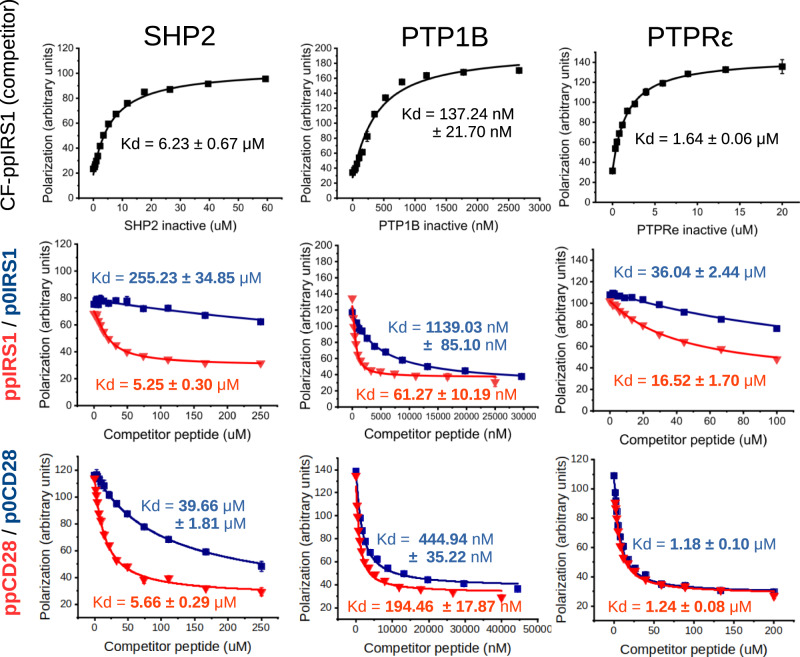
Affinity measurements of phosphatase substrates. Affinity measurements between inactive (Cys → Ser) mutant tyrosine phosphatase domains and IRS1 and CD28-derived YxxM[ST]P phosphopeptides (*n* = 3 technical repeats for each curve showing mean and ±SD for points). The upper lane indicates the direct fluorescence polarization titrations against a carboxyfluorescein-labelled pYpS-IRS1 (CF-ppIRS1) peptide (Y632 + S636), while the lower lanes show competitive titrations against the matching unlabelled pTyr or pTyr+pSer peptides. The effect of IRS1 serine phosphorylation is major on SHP2 binding, while smaller on PTP1B. The last examined phosphatase, PTPRε does not seem to be affected by the presence of pSer. Similar, albeit smaller effects are seen on CD28 phosphopeptides (lowest lane), as the competitive FP titrations with the singly Tyr phosphorylated (blue) or the doubly, Tyr+Ser phosphorylated (red) CD28 peptides show. Source data are provided as a Source Data file.

Human IRS1 has 4 distinct PI3 kinase binding tyrosine phosphorylation sites flanked at the +4 position by a Ser-Pro site, a target of proline-directed kinases^12^. Most of these compound sites (3 out of 4) are also conserved in IRS2. From these highly similar tandem motifs, we selected the 2nd motif from human IRS1 surrounding Y632, to act as an in vitro model peptide of 15 amino acids (GRKGSGD**Y**MPM**S**PKS). The chosen sequence contains two of the most highly phosphorylated sites in IRS1 at its tyrosine and serine according to the PhosphoSitePlus database^13^. This model peptide was synthesized in multiple varieties, with phosphotyrosine only (p0IRS1: pY632) or dually phosphorylated on both tyrosine and serine residues (ppIRS1: pY632 + pS636). Binding affinity of peptides against catalytically inactive tyrosine phosphatase enzymes was measured in fluorescence polarization titrations. For this assay, the isolated catalytic domain of SHP2 was cloned and expressed with a substrate trapping Cys459→Ser mutation^14^. We also tested two other, distantly related phosphatases implicated in negative regulation of insulin signaling: the well-known PTP1B and the less explored receptor tyrosine phosphatase PTPRε^15–17^. Comparison of titrations against the singly versus dually phosphorylated model peptides showed that binding of potential IRS1 substrate sites is enhanced by the Ser phosphorylation to a variable degree. Binding to SHP2 was extensively (∼49-fold increase), to PTP1B moderately (∼19-fold increase) and to PTPRε slightly (∼2-fold increase) improved by addition of the second, pSer modification (Fig. 2 and Supplementary figs. 4–6). While the fact that PTP1B preferentially recognizes tandem phosphotyrosine-containing substrates with its twin-charged pockets is well known^18^, a similar behaviour for SHP2 on asymmetrically phosphorylated, pTyr+pSer epitopes was unexpected.

**Fig. 3:**
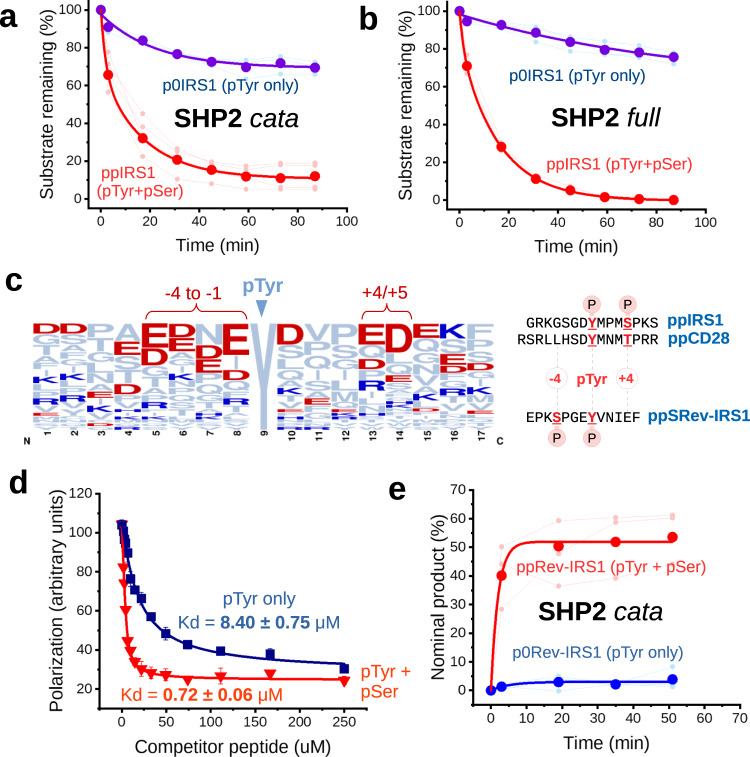
Activity measurements with SHP2 and the role of + 4 and −4 phosphorylation. Dephosphorylation assays with SHP2 catalytic domain (**a**) or full-length activated SHP2 (**b**) using either the tyrosine-phosphorylated p0IRS1 or the dually-phosphorylated ppIRS1 peptide as a substrate (at 50 μM concentration, enzymes were applied at 25 nM and 1 μM, respectively). Thin lines and small dots indicate individual repetitions of the experiment (*n* = 3/*n* = 5 for A, *n* = 3*/n* = 3 for B), while the thick lines are exponential curves fitted over their means (large dots). **c** Frequency logo of 30 direct SHP2 substrate sites described in the literature. Negatively charged amino acids written in red, while positively charged ones are in blue. Two regions show enrichment of negative charges: one at +4/5 and another at −4 to −1. These sites correspond to Ser/Thr phosphorylation sites in our peptides. **d** Fluorescence polarization assays with the tyrosine phosphorylated or dually phosphorylated SRev-IRS1 peptides show an enhancement of binding comparable to +4 Ser/Thr phosphorylated substrates (*n* = 3 technical replicates, with mean ± SD displayed for each point). **e** Dephosphorylation assays monitoring the product formation (expressed as % of substrate peaks) showing superior reaction rates with the doubly-phosphorylated ppRev-IRS1 peptide versus its phosphotyrosine-only counterpart (p0Rev-IRS1) (*n* = 3 for both curves). Thin lines and small points show individual experiments, while the thick lines are exponential curves fitted over their means (large dots). Source data are provided as a Source Data file.

Next, we set out to test if binding affinity differences translate into actual activity differences. We performed a tyrosine dephosphorylation assay (using wild-type catalytic domains of SHP2, PTP1B, and PTPRε) with the capillary electrophoresis technique, to monitor substrate consumption or product generation. The assays (performed at varying substrate concentrations) indicated no difference in the dephosphorylation rate of ppIRS1 and p0IRS1 peptides by PTPRε. In contrast, PTP1B showed a marked preference for the serine phosphorylated ppIRS1, albeit only at low substrate concentrations (<200 nM) (Supplementary fig. 3). Surprisingly, the merely tyrosine phosphorylated peptide was a very poor substrate for SHP2, with catalytic rates being negligibly low even at high p0IRS1 peptide concentrations, but the serine-tyrosine doubly phosphorylated peptide was efficiently dephosphorylated at its tyrosine site (Fig. 3a). To dismiss the possibility that the unexpectedly large difference in the SHP2 catalytic rates were due to an artificially distorted, truncated phosphatase domain, we also expressed and purified the full-length, activated SHP2 protein. In the latter, the auto-inhibition was disrupted by the oncogenic E76K mutation^19^. This construct was catalytically active on our peptides, and it showed the same behaviour as the isolated catalytic domain of SHP2 (Fig. 3b).

According to the ‘Writer-Reader-Eraser’ model of tyrosine kinase signaling, it is the balance of phosphorylation/dephosphorylation and effector binding that determines the output of a given kinase system. Therefore, we also tested if the serine modification in question would affect the binding of the effector PI3 kinase: To this end, the twin SH2 domains of the PIK3R1 regulatory subunit of PI3K (also known as p85) were also subjected to in vitro fluorescence polarization titrations. Although the nSH2 and cSH2 domains bound with different strengths to the model peptides, the effect of serine phosphorylation was negligible in all cases (see Supplementary fig. 1). This observation refutes earlier hypotheses that PI3K binding would be weakened upon Ser phosphorylation^12^. We also performed phosphorylation assays with recombinant InsR kinase domain, with an appropriate substrate peptide pair (0pIRS1 and 00IRS1, with or without the pSer), using capillary electrophoresis. These assays also failed to indicate any major effect of the +4 Ser phosphorylation on InsR activity (Supplementary fig. 2). Based upon these results we suggest that the Tyr-flanking Ser phosphorylation selectively modulates the phosphatase activity, supporting earlier findings on the role of SHP2 in insulin pathway desensitization^5^.

To corroborate these results *in cellulo*, we created a mutant IRS1 expression plasmid with all four YxxMSP serines mutated to alanine (IRS1-SA) and transfected a constitutively InsR-expressing HEK293-T cell line with this construct in combination with SHP2-E76K. Although the curves following insulin stimulation displayed high variability, the baseline Y612 phosphorylation was clearly increased on all western blots due to the Ser-Ala mutation (see Supplementary figs. 13–15).

### SHP2 recognition is also enhanced by Ser/Thr phosphorylation of various other sites

After confirming the pSer/pThr dependentregulation of SHP2 in vitro, we theorized that it is unlikely to be restricted to the Y632-S636 site of IRS1. While the tandem YxxMSP motifs are highly similar within IRS1, and an enhanced dephosphorylation was also reported on the Ser-phosphorylated Y612-S616 pair^5^, such motifs are not limited to IRS1 and IRS2. A highly similar RSRLLHSD**Y**MNM**T**PRR motif has also been described in the T-cell costimulatory receptor CD28, where it plays a crucial role in T-cell activation, proliferation, and immune regulation. This CD28 region has also been shown to be regulated by SHP2 (recruited in *trans*, by the PD1 receptor)^20^. Although the pThr residue (that is situated at a CDK kinase consensus site) has been found to form a mutational hotspot in T-cell lymphomas^21^, its role has so far been unclear. Our experiments with the dually phosphorylated CD28 peptide (ppCD28) and its pTyr-only counterpart (p0CD28) were performed similarly as described earlier with IRS peptides. Similarly to the latter, +4 pThr modification did not affect PI3 kinase SH2 domain binding to CD28 (see Supplementary figs. 11, 12). In contrast, matching our results with the phosphatases acting on IRS1, this modification did increase PTP1B (∼2-fold) and SHP2 (∼7-fold) binding, while PTPRε was unaffected (Fig. 2 and Supplementary figs. 7–9). These experiments suggest that SHP2 consistently binds pTyr motifs with increased affinity if they are Ser/Thr phosphorylated at +4.

Ser/Thr phosphorylation at the +4 position might not be the only way to increase SHP2 catalysis. Since phosphorylated Ser or Thr amino acids can often be substituted by non-phosphorylated analogues, we asked if known SHP2 substrates had a negative charge preference (e.g., Asp or Glu) surrounding the the pTyr residue. To this end, a total of 30 well-established, direct SHP2 substrate sites were collected from the literature, from proteins other than IRS1 (Supplementary Tables 1 and 2). Since SHP2 can also alter Tyr phosphorylation levels by indirect mechanisms (e.g., through the regulation of Src kinases or binding and shielding from phosphatases), we only included sites with a strong indication of being direct targets of SHP2^22^. A sequence logo compiled from these sites indicates a clear preference for negatively charged amino acids (Glu/Asp) at the +4 and +5 positions (Fig. 3c). Interestingly, an even stronger preference for negative charges is seen from positions −1 to −4 relative to the pTyr residue^8^. The latter phenomenon is apparently a more generic feature of multiple tyrosine phosphatase substrates^23^.

To prove that a negative charge at the −4 position in a substrate also increases the catalytic activity of SHP2, we selected the Y896 site of IRS1, where a conserved Ser phosphorylation site flanks this GRB2-binding pTyr from the N-terminal side (HPPEPK**S**PGE**Y**VNIEFGS motif). A pair of peptides was synthesized, one containing the pSer modification in addition to the pTyr (ppRev-IRS1) and one without pSer (p0Rev-IRS1), and their SHP2 mediated dephosphorylation was tested by the capillary electrophoresis-based assay. These results show that there is a considerable difference between the dually phosphorylated ppRev-IRS1 and singly phosphorylated p0Rev-IRS1 peptides in terms of SHP2 catalytic efficacy, matching the large difference in the binding affinity of shortened reverse IRS1 peptides (EPK**S**PGE**Y**VNIEF) (Fig. 3d, e and Supplementary fig. 10). The rate of dephosphorylation is greatly enhanced by the −4 phoshoserine, similarly to what we had seen with the +4 modifications earlier using the IRS1 Y632-S636 site containing peptide. Conversely, the −4 Ser phosphorylated peptide bound one magnitude stronger to the inactive SHP2 catalytic domain despite both SRev-IRS1 peptides already containing a negatively charged Glu at the +4 position. We also compared the phenomenon to the catalytic activity of InsR kinase domain on Y896 containing peptides, this time with a peptide pair containing a non-phosphorylated and a Ser phosphorylated motif, respectively. Again, there were no major differences detected in InsR kinase activity on these two peptides (see Supplementary fig. 2). Taken together, this indicates that SHP2 can be regulated by diverse asymmetric (pSer/pThr + pTyr) phosphorylation sites, with a similar outcome.

### X-ray crystallographic structures of SHP2 with three different substrate peptides

To gain detailed structural insight into the substrate recognition by SHP2, we crystallized the inactive phosphatase domain with three different, multi-phosphorylated peptides. Two of them represent substrates modulated by a phosphorylated +4 position: the 15 aa long pY632-pS636 peptide from human IRS1 (ppIRS1) and the 16 aa long pY191-pT195 peptide from human CD28 (ppCD28); The third peptide, the 13aa long pS892-pY896 (ppSRev-IRS1, shortened Rev-IRS1 peptide) from human IRS1 represents a reverse orientation, modulated by a −4 phosphorylation site. While the native SHP2 (catalytically inactive, C459S mutant) phosphatase domain failed to crystallize, we did obtain peptide-loaded crystals after detailed structural optimization. Our protein alterations did not impact the surroundings of the catalytic site (only truncating a flexible loop at 315–323 and removing the mobile flanking region 219–245 from the phosphatase domain, see Supplementary fig. 16). This careful protein engineering allowed the formation of new monomer contacts and greatly facilitated crystal lattice formation. We obtained crystals from all three complexes with decent resolution under X-ray diffraction (1.5 to 1.9 Å), enabling the study of substrate peptides (See Table 1 and Supplementary figs. 17–20).

**Table 1:** Details of crystal structure solution and refinement

Data collection	SHP2-ppIRS1	SHP2-ppCD28	SHP2-ppSRev-IRS1
Space group	C 2 2 2_1_	C 2 2 2_1_	C 2 2 2_1_
**Cell dimensions**			
*a*, *b*, *c* (Å)	54.89 81.80 147.63	54.19 82.36 147.76	56.14 80.54 149.51
*α*, *β*, *γ* (°)	90.00 90.00 90.00	90.00 90.00 90.00	90.00 90.00 90.00
Resolution range (Å)	147.63–1.56 (1.56–1.53)	49.25–1.90 (1.94–1.90)	46.05–1.48 (1.51–1.48)
CC_1/2_	1.000 (0.885)	1.000 (0.646)	0.999 (0.600)
^a^*R*merge	0.055 (1.003)	0.048 (1.344)	0.041 (2.159)
<*I*/σ(*I)*>	24.3 (2.5)	26.1 (1.8)	26.9 (1.5)
Completeness (%)	100.0 (100.0)	99.9 (99.5)	99.7 (99.2)
Redundancy	13.2 (13.5)	12.9 (9.5)	13.3 (13.5)
No. of reflections	669091 (33413)	341960 (15737)	754810 (37051)
**Refinement**			
*R*_work_/*R*_free_	0.1762/0.1972	0.1801/0.2069	0.1645/0.1961
No. of atoms	2578	2400	2459
Protein	2385	2344	2329
Ligand/ion	33	6	6
Solvent	160	50	124
B-factors (Å^2^)	30.07	52.43	39.34
Protein	29.61	52.54	39.28
Ligand	43.98	66.16	34.32
Solvent	34.18	45.71	40.73
**Ramachandran**			
Favored (%)	97.51%	98.56%	98.19%
Allowed (%)	2.14%	1.08%	1.45%
Outliers (%)	0.36%	0.36%	0.36%
Rotamer outliers (%)	0.38%	0.00%	0.00%
**R.m.s deviations**			
Bond lengths (Å)	0.016	0.006	0.010
Bond angles (°)	1.376	0.875	1.032
PDB ID	7PPL	7PPN	7PPM

^a^R_merge_ = Σ_hkl_Σ_i_|I_i_(hkl)-<I(hkl)>|/Σ_hkl_Σ_i_ I_i_(hkl)

Summary of the X-ray crystallographic analysis of our three SHP2-substrate peptide complexes, resolved between 1.51 to 1.94 Angstroms. Although all three belong to the same symmetry group, there are subtle differences in crystal contacts and hence, also in cell paramaters

**Fig. 4:**
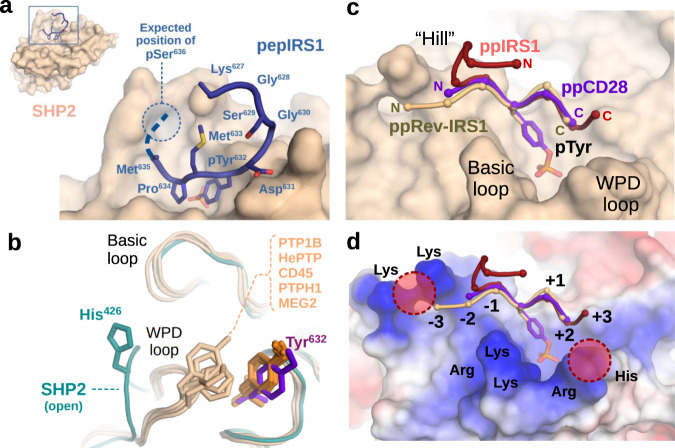
Crystal structures of SHP2 with dually phosphorylated peptides. **a** Overview of the SHP2-ppIRS1 (pY632-pS636) complex, with all amino acids of the dually phosphorylated substrate seen in the X-ray structure. The expected position of pSer636 is also indicated. **b** Our structures differ from most other phosphatases, such as PTP1B (pdb:1EEO), HePTP (pdb:3D42), CD45 (pdb: 1YGU), PTPH1 (pdb:4QUM) and MEG2 (pdb:4ICZ) as having an open catalytic WPD loop (cyan), while already pre-loaded with the substrate. The aromatic amino acid capping the pTyr is indicated on each structure (corresponding to His426 in SHP2). The pTyr residue is also slightly dislocated in our structures (ppIRS1 complex: shown in purple), with respect to other tyrosine phosphatases. **c** Comparison of all three crystal structures showing very similar main chain geometry between positions −2 to +2 from the pTyr, and divergently positioned flanks. **d** The pSer or pThr residues cannot be traced in any of the three complexes, despite the highly charged SHP2 surface these substrate peptides are facing against, near both the +4 and the −4 positions of the peptides.

Comparison of the three crystal structures shows that substrate peptides are bound to SHP2 very similarly (especially from postions −2 to +2 relative to phosphotyrosine) in all cases (Fig. 4a, c). Interestingly, large segments of all three peptides, including all phosphorylated −4 or +4 Ser and Thr residues turned out to be disordered in the crystals (Supplementary figs. 21–23). This is in spite of the fact that the peptide chains are expected to run close to two, positively charged regions in SHP2: Position −4 of the of the substrates close to residues Lys274-Lys280 and position +4 close to the loop carrying residues Arg362-Lys364 (Fig. 4d). In case of the ppSRev-IRS1 peptide, this would allow up to two simultaneous charge contacts (pSer at −4 and Glu at +4) towards the phosphatase, yet none were resolved as traceable on the electron density maps (Supplementary figs. 21–23 and 27–33). Only the distant N-terminus of the ppIRS1 peptide could be traced weakly (thanks to a crystal contact), but this stretch lacks pSer/pThr residues. Mass spectrometric measurements obtained from the mixtures also helped us to exclude the possibility of accidental dephosphorylation during crystallization (Supplementary figs. 34–36). This unexpected observation suggests that the flanking phosphates are coordinated by SHP2 as a fuzzy complex, and not in a single, well-defined geometry (Supplementary fig. 37). It is also worth noting that 2 of our 3 peptides would be highly positively charged at neutral pH without the phosphates: Thus, strong intra-peptide bonds might form upon phosphorylation, increasing their conformational complexity. A similar case is presented by the PTPN3 tyrosine phosphatase, where crystal structures with a highly negatively charged substrate similarly failed to resolve the peptide C-terminus (Supplementary figs. 24,25)^24^.

On a closer look, these complexes display another unusual feature. Our solved crystal structures clearly present SHP2 with an open WPD loop, despite the catalytic site being occupied by a phosphotyrosine peptide (Fig. 4b). This open conformation differs from most other tyrosine phosphatases (PTP1B, CD45, HePTP, etc., with rare exceptions), whose apo structures (i.e., without any ligand) present the open, and the peptide-loaded structures the closed conformation^25–29^. Movement of the WPD loop is also necessary for the Asp amino acid to lock into catalytic position. In the latter state, an aromatic amino acid from the top of the WPD loop (corresponding to His426 in SHP2) moves to cover the pTyr with a π- π stacking interaction. However, in our SHP2-ppIRS1 structure, the same position appears to be occupied by a proline amino acid (Pro634) from the substrate itself, also causing a mild dislocation of the phosphotyrosine residue (Fig. 4b). Likewise, the other two structures show an asparagine from the substrate at the same position (+2), stacking against the similarly dislocated pTyr residue. This unusual arrangement means that SHP2 substrate peptides are captured and held in a state that differs from the typical catalytic state (characterized by WPD loop closure) in other tyrosine phosphatases.

### In silico models of SHP2 pSer /pThr amino acid recognition and their validation

Despite our best attempts, the phosphoserines or phosphothreonines could not be located on any of the density maps; This suggests that the N- and C-terminal ends of the peptides are highly flexible and exist as an ensemble of different conformations even at its phosphatase-bound state. Therefore, we resorted to molecular modelling to better understand the coordination of pSer/pThr. We constructed a representative ensemble of initial models for each crystal structure separately, then subjecting them to flexible docking by Haddock using minimal restraints true to all tyrosine phosphatases (Supplementary table 3). The resulting clusters with the best energy finally shed light on the coordination of pSer and pThr residues by SHP2 as well as intra-peptide bonds (Supplementary dataset 1).

**Fig. 5:**
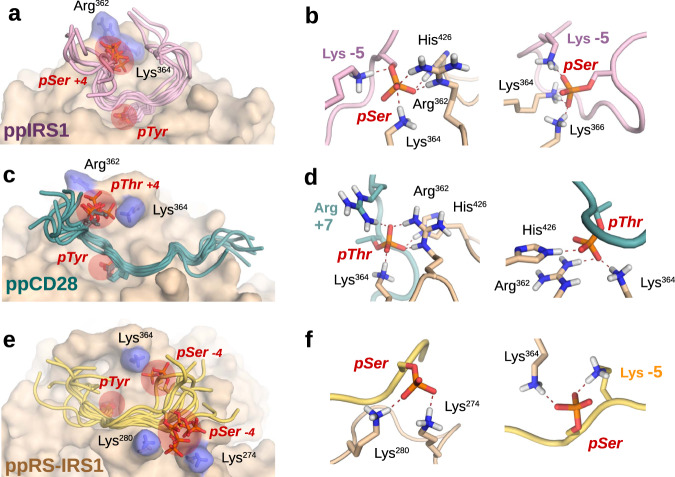
Haddock modelling of all three SHP2-substrate complexes. Structural ensembles from the lowest energy Haddock clusters obtained for the SHP2-ppIRS1 (**a**), SHP2-ppCD28 (**c**) and SHP2-ppSRev-IRS1 (**e**) complexes after docking a 20-state, structurally diverse input file for each structure. The three ensembles are shown from similar angles, providing visibility to the phosphate groups. Residues coloured in blue represent key pSer/pThr coordination points (the two phosphate groups are highlighed in red). Polar H-bonds are drawn as dotted lines on the right panels, detailing two possible phosphate coordination states per complex. **a**, **b** In the SHP2-ppIRS1 complex, the pSer residue is typically coordinated by the Arg362, Lys364, Lys366, and His426 residues, in addition to intra-chain charged H-bond contacts, mostly by the −5 lysine residue to the N-terminus. **c**, **d** In the SHP2-ppCD28 complex the same charged amino acids of SHP2 coordinate the pThr side chain, in addition to a potential intra-chain contact by the +7 arginine. **e** Docking of the SHP2-ppSRev-IRS1 complex revealed an ensemble of two main coordination modes. **f** One potential coordination point is provided by the charged surface Lys280–Lys274 pair, and another by the surface Lys364, where internal contacts by the −5 lysine can also help to stabilize this geometry.

While the phosphotyrosine was tightly locked to a single position in both the ppIRS1 and ppCD28 models, the phosphoserine remained flexible, and was to be located at a broad loop region defined by Arg362, Lys364 and Lys366 in the best models (Fig. 5). The same models also suggest that the highly charged N-terminal end of the peptide (the −5 Lys in ppIRS1 or the +7 Arg in ppCD28) can simultaneously also engage in intra-chain contacts with the phosphate group, further stabilizing the U-shaped conformation of the peptide ligand (Fig. 5). In the case of the ppsRev-IRS1 carrying a −4 pSer residue, a somewhat more complex picture emerged: Here, the phosphoserine was either coordinated by Lys274 and Lys280 (“Hill” region), or Arg278 and Lys364 (as well as Lys−5) in an alternative conformation (Fig. 5). What is more, the +4 position carrying a glutamate residue was loosely associated with the same loop region that coordinated the +4 pSer/pThr residues of the two previous complexes.

**Fig. 6:**
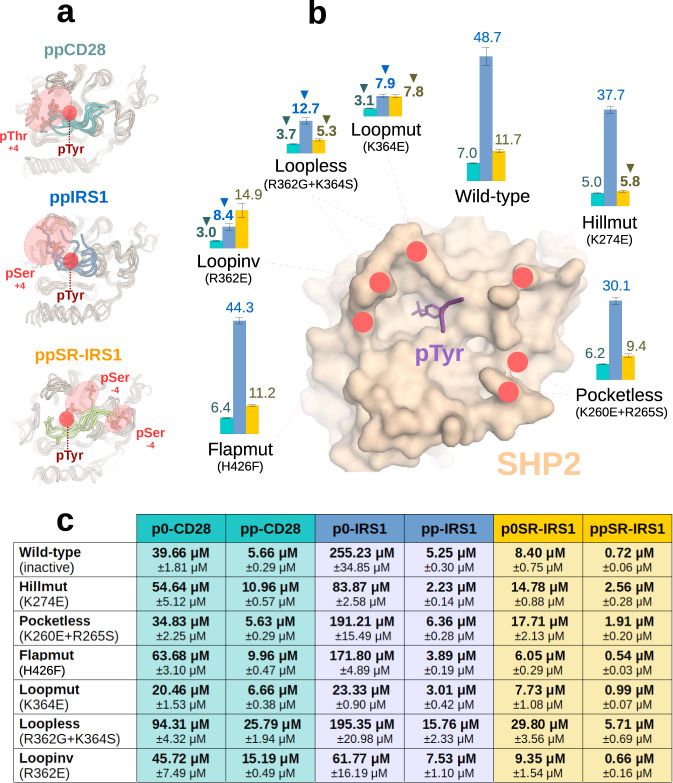
Experimental validation of structural models. **a** Aligned end states for three SHP2-substrate complexes after 10 ns MD simulations (a total of 5 for each complex), indicating the broader regions where the pSer or pThr amino acids are located. **b** The effect of SHP2 surface mutations on phosphoserine recognition. The surface figure indicates the positive charges altered in each mutant, while the bar plots and numbers indicate the relative binding strength of the two peptides (Kd(**pSer)**/Kd(**pTyr** + **pSer**) or pThr in case of CD28). The blue bars refer to the IRS Y632-S636 peptides, the green bars show the behaviour of the CD28 Y191-S195 peptides, while the IRS1 S892-Y896 peptides are indicated by the yellow bars. The underlying experiments had *n* = 3 technical repeats, yielding Kd values with error estimates after nonlinear curve fitting (Supplementary note 1). Mean and ±SD estimates for the Kd ratio distributions shown on the bar plots were calculated using a Monte Carlo method (5000 randomly simulated points for each ratio, see Supplementary note 2 for more details) Reductions in the relative binding strengths that are also expected based on the MD simulations, are indicated by small arrows, **c** All the dissociation constants used to calculate Kd ratios on panel b are tabulated below (see Supplementary figs. 40–57 for the curves, all with *n* = 3 technical repeats, with error estimate for each Kd). For two of the three peptides, phosphorylated at the +4 position, the largest effect is always seen with mutations involving Arg362 and Lys364 (Loopmut/Loopless and Loopinv constructs). For the last pair of peptides, phosphorylated at the −4 position, the largest effects are seen when mutating either Lys274 or Lys364 (Hillmut/Loopless constructs). Source data are provided as a Source Data file.

To validate the results of crystallographic and modelling studies, we analyzed the positively charged surface of the SHP2 catalytic domain and generated a set of mutants around the substrate binding pocket. Apart from the highly conserved pTyr coordination site and two buried, structural arginine residues, other positively charged positions are varied and mostly specific to SHP2 (Supplementary figs. 37–39). To neutralize or invert these charges, a series of surface mutants were constructed: R362E (Loopinv), R362G + K364S (Loopless), K364E (Loopmut), K274E (Hillmut), H426F (Flapmut) and K260E + R265S (Pocketless). Next, we searched for mutants that would decrease the affinity proportion between the dually phosphorylated and the phosphotryosine-only peptide pairs (e.g., the p0IRS1 / ppIRS1 pair). Binding affinities were assayed by fluorescence polarization-based titrations (see Supplementary figs 40–57). While the dissociation constants varied, mutations to the “Loop” region (containing Arg362 and Lys364) consistently lowered the difference in the p0IRS1-ppIRS1 pair, unlike other regions. In order to show that the effect is not unique to this particular peptide, we also repeated the same assay with the CD28-derived peptide pair (p0CD28 and ppCD28). As expected, the “Loop” region mutants decreased the proportion of Kd(p0CD28)/Kd(ppCD28) to the highest degree (Fig. 6). Albeit this region falls close to the pTyr coordinating catalytic pocket, it is unlikely that mutations would have interfered with pTyr binding. For example, the K364E mutant actually bound stronger to both ppIRS1 and p0IRS1 peptides than to the wild-type protein. Notably, the proportion of dissociation constants never reached unity in any mutant, suggesting the presence of intra-peptide salt bridges in the phosphorylated variant. These experimental results are all well in-line with the X-ray structures and strongly support our in silico models of phosphoserine coordination.

The coordination of the ppSRev-IRS1 peptide, phosphorylated at the −4 serine was expected to be rather different from ppIRS1 and ppCD28 based on our models. Affinity measurements with our SHP2 surface mutants also corroborated the docking results, with the greatest effect (i.e., the smallest relative proportion) seen when mutating either the complete “Loop” (R362G + K364S) or “Hill” (K280E) regions (Fig. 6). The combination of these moderate effects further supports our previous structural data, revealing interchangeability of the positively charged regions in the coordination of the phosphoserine residues.

To gain more insight into the stability of phosphate coordination over time, we also performed all-atom molecular dynamics (MD) simulation on our docking-derived structural models. All short (10 ns) MD runs on GROMACS display a relatively high mobility of peptide N- and C-temini (beyond positions −2 and +2). We observed a dynamic exchange of pSer/pThr coordinating side chains, explaining the lack of a well-defined density in X-ray strucures (See Supplementary dataset 3 for the movies, Supplementary dataset 2 for the PDB files and Fig. 6a for the end states after 10 ns). MD simulations also lend strong support for intra-chain charge contacts in all three SHP2-phosphopeptide complexes. The top five residues coordinating the Ser/Thr phosphate were Arg362, Lys364, Lys366 and His430 as well as an intra-peptide side chain (Lys-5 or Arg+7) in both the ppIRS1 and ppCD28 simulations (+4 phosphorylated). In case of the ppSRev-IRS1 (−4 phosphorylated), the pSer coordination dynamically alternated between the “Hill” site (Lys270, Lys274) and the “Loop” site (Lys364, Arg278), with an intra-chain Lys-5 also contacting this residue in the majority of runs. At the same time, mobility of the pTyr side chain at the catalytic pocket was minimal. Compared with the doubly phosphorylated models, the singly phosphorylated peptides are considerably more flexible, as they are not tethered to the surface by the same charge contacts (Supplementary dataset 2 and Supplementary figs. 74–79). The adjacent charged loop and the WPD loop also appear to be highly mobile (see the results of principal component analyses of the complex with the doubly-phosphorylated peptide in Supplementary figs. 74–76 and the singly-phosphorylated peptide under Supplementary figs. 77–79), although no closure of the catalytic site can be observed in the studied time frames.

### SHP2-substrate co-crystal structures represent a pre-catalytic state

In contrast to most of the published tyrosine phosphatase structures, our crystals contain enzymes with a clearly open WPD loop. In this open conformation, the key catalytic residue Asp425 is too far away from the phosphotyrosine to mediate hydrolysis of the phospho-cysteine catalytic intermediate. Adding to the mystery, earlier publications suggested that the D→A mutation is insufficient to create a substrate-trapping, phosphatase-dead SHP2, unlike other phosphatases^14^. These findings raised the possibility of alternative catalytic mechanisms in SHP2. At the same time however, the canonical catalytic apparatus of all known tyrosine phosphatases is clearly conserved in SHP1 and SHP2 as well.

**Fig. 7:**
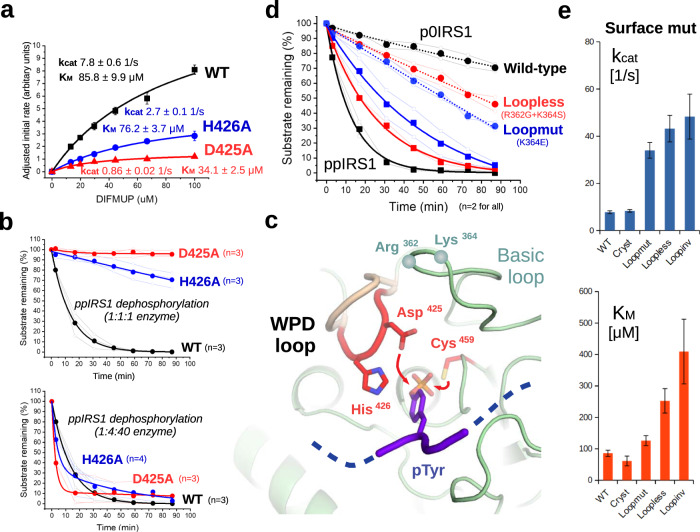
Validation of SHP2 catalytic apparatus. **a** Kinetic experiments (*n* = 3 technical repeats, mean and ±SD plotted for each point) with the small-molecule substrate DIFMUP indicate that mutation of either Asp425 (required for nucleophilic attack on the phosphocysteine intermediate) or His426 (required for optimal positioning of the pTyr) impairs catalytic activity of SHP2, with a more-or-less pronounced effect on k_cat_. **b** Capillary electrophoresis (CE)-based kinetic assays with the ppIRS1 substrate peptide display slowed reaction rates mirroring those seen with DIFMUP. With the enzyme quantities set to 1:4:40 the dephosphorylation rates became more similar. Thin lines and small points show individual experiments, the thick lines are exponential curves fitted over their means (large dots). **c** Model of the catalytic state of SHP2, illustrating the closure of the WPD loop with key residues involved in catalysis (D425, H426, C459) or controlling the formation of the closed catalytic state (R362, K364). **d** CE-based dephosphorylation assays with the active Loopmut (K364E) and Loopless (R362G + K364S) mutants vs. WT SHP2 catalytic domain (*n* = 2). Dotted lines and circles show enhanced dephosphorylation of non-cognate p0IRS1 peptide substrate by mutants, while solid lines and squares the slowed reaction on the cognate ppIRS1 substrate peptide. **e** Bar plots of fitted values and error estimates of k_cat_ and K_m_ for various other surface mutants using the small-molecule pTyr mimic DIFMUP.These plots show the increased non-specific catalytic activity observed upon altering R362 and/or K364, as well as the correlated change of k_cat_ and K_m_. WT: SHP2 catalytic domain 219–528, Cryst: Δ(219–245)-Δ(315–323)+GSSG crystallization construct, Loopmut: K364E, Loopless: R362G + K364S, Loopinv R362E as on Fig. 6, but all in a catalytically active form (no inactivating C459 mutation). The original kinetic experiments had *n* = 3 technical repeats. Source data are provided as a Source Data file.

To address this discrepancy, we created two different mutants to the core catalytic apparatus of SHP2: the Asp425→Ala (D425A) mutant lacking the nucleophile responsible for the activation of water molecules as well as the His426→Ala (H426A) mutant removing the aromatic amino acid assisting the loop closure. Next, we measured Michaelis-Menten kinetic parameters of mutant versus the wild-type catalytic domain using the phosphotyrosine mimic synthetic substrate 6,8-difluoro−4-methylumbelliferyl phosphate (DIFMUP). These measurements indicate that the H426A mutant is mildly impaired in its activity (k_cat_ reduced) approximately to 1/3–1/5, while the D425A mutant was much more severely impaired (k_cat_ reduced by about a magnitude compared to the wild-type catalytic domain) yet not completely dead. (Fig. 7a and Supplementary figs. 58–67) Kinetic curves taken with a peptide substrate (ppIRS1) revealed similar changes to the catalytic activity of mutants, with approximately ∼4-fold H426A and ∼40-fold D425A mutant enzymes generating similar substrate consumption curves to the wild-type enzyme (Fig. 7b). These findings show that the Asp425 and His426 residues are in fact required for efficient catalysis of SHP2 and imply a transient closure of the active site of SHP2 to a conformation similar to most other tyrosine phosphatases. To visualize this canonical catalytic state of SHP2, we also created a set of models with a closed WPD loop modelled after PTP1B with the help of Haddock and subjected them to MD simulations for 10 ns. However, we observed that the closed state of the WPD loop was rather unstable in multiple runs, with a substantial fluctuation of its distances (D425, H426) from the substrate pTyr moiety (Supplementary dataset 2 and Supplementary figs. 80–82).

We also measured the activity of surface mutant SHP2 enzymes on the small molecule pTyr mimic DIFMUP. Interestingly, the catalytic activity (k_cat_ but also K_m_) of charged loop mutants is considerably increased versus the wild-type catalytic domain (Fig. 7e). On the other hand, this gain of function was reflected differently on more complete peptide substrates: Here, the loss of the charged loop only accelerated dephosphorylation of a non-specific substrate (singly phosphorylated p0IRS1 peptide), while simultaneously decreasing SHP2 activity on a specific substrate (doubly phosphorylated ppIRS1 peptide). Therefore, Arg362 and Lys364 also contribute to specificity by selectively restraining the activity of SHP2 on nonspecific substrates (Fig. 7c and d). The correlated changes of K_m_ and k_cat_ (on DIFMUP) could be indicative of a primarily k_cat_-driven effect, with the assumption that k_cat_ » k_off_ for the hyperactive mutants (Fig. 7e and Supplementary fig. 58–66). Our findings also suggest that the charged loop (with K364 and especially R362) is not only important for pSer/pThr recognition, but also for the maintenance of the pre-catalytic state, reducing background activity.

Our finding that the pre-catalytic “open” state of SHP2 is unusually stable compared to other tyrosine phosphatases is also supported by the binding modes of small molecules. Many orthosteric inhibitors were developed against SHP1 and SHP2. All their published crystal structures (with chemically unrelated inhibitor structures) reveal binding at the “open” conformation and stabilization thereof. The competitive inhibitors Tautomycetin D1 (PDB: 3MOW) and Cefsulodin derivatives (PDB: 4RDD) bind in poses resembling our peptide substrates (see Supplementary fig. 83), with an open WPD loop^30,31^. But other small molecules likely occupy similar poses at the catalytic site of SHP2. To model their binding modes, we collected four, chemically rather different high-affinity inhibitors (PHPS1, NSC87877, C21 and SPI-112) from the literature, known to act competitively^32–35^. Docking simulations using Autodock Vina supports that these inhibitors do recognize features unique to SHP2 (such as the highly charged Arg362-Lys364 loop)^36^. Moreover, free enthalpy estimates suggest that they are capable to bind to a stably “open” (pre-catalytic) conformation of the catalytic site (Supplementary fig. 84).

## Discussion

In our studies, we focused mostly on the structural aspects of SHP2-IRS1 interaction. However, the peculiar regulation of SHP2 phosphatase represents an entirely new model of pSer/pThr action. What is more, such cross-regulation of tyrosine phosphorylation pathways by serine-threonine kinases does not seem to be limited to the IRS or CD28 proteins only. If we restrict ourselves to the +4/+5 proline-directed Ser/Thr phosphorylation sites following a phosphotyrosine (that likely enable dephosphorylation specifically by SHP2), we can still find a good number of unexplored candidates in the human proteome (See Table 2). Judged by the conservation of motifs, it seems that a joint control of SHP2 action by a tyrosine kinase and a proline-directed Ser/Thr kinase (such as MAPK) might also be realized on GAB-family proteins. Here, the ERK2 kinase is known to feed back onto the adaptor proteins GAB1/2 in a complex manner, to restrict EGFR pathway activation^37^.

**Table 2:** Prediction of pSer/pThr modulated SHP2 target pTyr motifs

Protein name	Category	Sequence	Matched consensus	Predicted effector(s)
**AFAP1L2**	Cytoskeletal	esdrv**Y**LDL**T**Pvksfl	Y[ILV].[ILV][ST]P; Y.[DE].[ST]P	PLCγ; SRC
**TNS1**	Cytoskeletal	asdgq**Y**ENQ**S**Peatsp	Y.N.[ST]P	GRB2
**TRIOBP**	Cytoskeletal	rqald**Y**VEL**S**Pltqas	Y[ILV].[ILV][ST]P; Y.[DE].[ST]P	PLCγ; SRC
**BCR**	RhoGEFs/GAPs	qdglp**Y**IDD**S**Psssph	Y.[DE].[ST]P	SRC
**PYK2**	Misc pTyr signaling	ldpmv**Y**MNDK**S**Pltpek	Y.N..[ST]P	GRB2
**CD28**	Receptor/adaptor	llhsd**Y**MNM**T**Prrpgp†	Y..M[ST]P; Y.N.[ST]P	PI3K; GRB2
**GAB1**	Receptor/adaptor	iqean**Y**VPM**T**Pgtfdf	Y..M[ST]P	PI3K
**GAB2**	Receptor/adaptor	nsqsv**Y**IPM**S**Pgahhf	Y..M[ST]P	PI3K
**IRS1**	Receptor/adaptor	htddg**Y**MPM**S**Pgvapv†	Y..M[ST]P	PI3K
	Receptor/adaptor	kgsgd**Y**MPM**S**Pksvsa†	Y..M[ST]P	PI3K
	Receptor/adaptor	vdpng**Y**MMM**S**Psggcs	Y..M[ST]P	PI3K
	Receptor/adaptor	pctgd**Y**MNM**S**Pvgdsn	Y..M[ST]P	PI3K
**IRS2**	Receptor/adaptor	lpngd**Y**LNV**S**Psdavt	Y.N.[ST]P; Y[ILV].[ILV][ST]P	GRB2; PLCγ
	Receptor/adaptor	gaddg**Y**MPM**T**Pgaala	Y..M[ST]P	PI3K
	Receptor/adaptor	crsdd**Y**MPM**S**Pasvsa	Y..M[ST]P	PI3K
	Receptor/adaptor	gdsdq**Y**VLMS**S**Pvgril	Y..M.[ST]P	PI3K
**HNRNPK**	mRNA binding	psrrd**Y**DDM**S**Prrgpp	Y..M[ST]P; Y.[DE].[ST]P	PI3K; SRC
**LARP1**	mRNA binding	pespn**Y**RNTR**T**Prtprt	Y.N..[ST]P	GRB2
**DPF2**	Gene regulation	ldded**Y**EED**T**Pkrrgk	Y.[DE].[ST]P	SRC
**KMT5A**	Gene regulation	gqski**Y**SYM**S**Pnkcsg	Y..M[ST]P	PI3K
**ZC3H4**	Gene regulation	kghrk**Y**REY**S**Ppyaps	Y.[DE].[ST]P	SRC

In silico prediction of phosphotyrosine motifs from the human proteome, flanked by regulatory phosphoserine sites at the +4/+5 position that could plausibly affect effectors (GRB2, PI3K, PLCγ or SRC kinases). Initial motif scans were performed by SlimSearch 4 (ref. 58), and motifs were subsequently filtered (using PhosphoSitePlus data) so that only *n* > 1 times detected phosphorylation sites were retained, that are conserved at least in mammals, excluding folded domain blocks. Motifs that were confirmed experimentally to affect dephosphorylation by SHP2 (this article and Choi et al.^5^), are marked with a † symbol.

Sticking with the IRS1/2 system, our structures together with earlier publications^5^ also support an elegant hypothetical model of desensitization in type II diabetes: In the absence of flanking serine phosphorylation, only the ‘forward’ signaling (i.e., PI3K activation) is active. However, with the phosphoserines in place, the forward signaling would never be efficient, as SHP2 will constantly keep removing the tyrosine phosphates. Then an increased insulin stimulation would be required to achieve the same PI3K stimulation while also feeding a futile cycle of phosphorylation-dephosphorylation reactions. We cannot help but speculate if this simple model captures a portion of the desensitization observed in insulin resistance due to Ser/Thr kinases. The effects seen upon SHP2 (PTPN11) gene disruption (conditional knockout models) are variable and highly tissue dependent, probably due to the pleiotropic role of SHP2 in various growth factor pathways. Nevertheless, multiple experiments support that inhibition of SHP2 activity in the liver (but not in the skeletal muscle or pancreas) can ameliorate insulin resistance in mouse models^5,38–40^. In accordance with animal experiments, individuals with hereditary SHP2 gain-of-function mutations (Noonan syndrome) display insulin resistance more frequently than the generic population, despite their overall leaner phenotype^41^.

Last but not least, our findings might have practical implications in pharmaceutical design and development. The role of SHP2 is especially intriguing on CD28, as these proteins are key downstream targets of anti-PD1 therapies in cancer^7^. Our results suggesting a multi-level SHP2 regulation (i.e., recruitment, unlocking and target site enabling by pSer/pThr) reinforce the idea that anti-PD1 antibodies and SHP2 inhibitors might be synergistic in immune activation^42^. As SHP2 is already a tempting therapeutic target for leukaemia as well as solid tumors^43,44^, a possible immunostimulatory action might boost the usefulness of such medicines. Similarly, if SHP2 inhibitors indeed turn out to enhance insulin sensitivity in clinical trials, they could potentially be useful to offset the deleterious, hypoglycaemic effects of anti-cancer PI3K inhibitors^45^; or they could be utilized as a component of oral anti-diabetic combinations. Currently the only clinically useful SHP2 inhibitors are of an allosteric nature^46^. However, the peculiarities of SHP2 substrate coordination (such as the widely open catalytic pocket when the peptide substrate is already loaded) might aid the development of better orthosteric inhibitors in the future. These could make full use of the preferred WPD-open conformation of SHP2 to achieve higher selectivity.

## Methods

### Cloning, protein expression & purification

The two SH2 domains of PI3KR1 (UniProt P27986; nSH2: 321−433 cSH2: 614–724) were cloned from HEK293T cDNA pool, and subsequently ligated into a modified pET vector encoding maltose binding protein (MBP) tag on the N-terminal and a hexa-His tag on the C-terminal end of the construct. Similarly, the catalytic, tyrosine phosphatase domains of PTPRε (P23469 first domain: 107–399) and PTP1B (P18031; 1–229) were cloned from a HEK293T cDNA pool, and inserted into a pBH4 vector encoding an N-terminal His6 tag (for the final binding experiments, an MBP-tagged version of PTPRε was employed). Full-length, GST-tagged SHP2 (UniProt Q06124-2) was cloned similarly, and its mutants were generated by site-directed mutagenesis (activated: E76K, inactive: C459S). The (active/inactive) SHP2 catalytic domains (219–528 with or without C459S) were sub-cloned into pBH4 vectors. The other inactive phosphatase mutants (Cys to Ser mutations: C215S for PTP1B and C335S for PTPRε) were generated by the QuickChange mutagenesis protocol. Surface mutants of SHP2 were constructed from the SHP2-C459S plasmid, using the QuickChange protocol. The D425A and H426A catalytic site mutants and all enzymatically active surface mutants were generated similarly, but from the plasmid encoding the wild-type catalytic domain of SHP2. Wild-type C-terminal FLAG-tagged human IRS1 was cloned between HindIII-NotI sites of pcDNA3.1(+) vector (#V79020 Invitrogen, Waltham MA USA). Ser/Ala mutant IRS1 was generated by replacing the region of S616/S636/S666/S736 positions with a cassette carrying S616A/S636A/S666A/S736A mutations. All constructs were validated by complete Sanger sequencing before protein expression and purification. The oligonucleotide sequences used are shown on Supplementary table 4.

Constructs were expressed in the BL21 (DE3) strain of *E. coli*. Briefly, transformed bacteria were grown to OD ∼0.5 in antibiotic-containing LB medium, when they were induced by the addition of 0.075 mM isopropyl-beta-D-thiogalactoside (IPTG), and kept at 18 °C overnight. Cells were harvested and centrifuged twice, after washing with phosphate buffered saline (PBS). The bacterial pellet was then resuspended in 30 ml lysis buffer (300 mM NaCl, 50 mM Na_2_HPO_4_ [pH = 8.0], 10 mM imidazole, 0.1% IGEPAL detergent, 2 mM beta-mercapto-ethanol [βME], 0.4 mM phenylmethylsulfonyl-fluoride and 2 mM benzamidine) and sonified until complete cell lysis with a Branson sonifier. The lysate was centrifuged at 20,000 rpm for 30 min and 1-1 ml pure Ni-NTA resin (Ni-sepharose fast flow, GE healthcare) was added to the supernatant. After rocking for 30 min on 4 °C, the resin was transferred to a manual column and washed with 40 ml of wash buffer #1 (300 mM NaCl, 50 mM Na_2_HPO_4_ [pH = 8.0], 40 mM imidazole, 2 mM βME) and wash buffer #2 (1000 mM NaCl, 20 mM TRIS [pH = 8.0], 20 mM imidazole, 2 mM βME). Proteins were eluted in one step with 10 ml elution buffer (200 mM NaCl, 20 mM TRIS [pH = 8.0], 400 mM imidazole, 10% glycerol and 0.1% IGEPAL), and immediately supplemented with 2 mM tricarboxyethyl phosphine (TCEP). Typical yields were 10 to 20 mg Ni-NTA-purified protein from 1 litre culture.

Ni-NTA purified stocks were subjected to anion exchange chromatography using an Äkta explorer instrument (Amersham Pharmacia) and Resource Q columns (GE healthcare). Prior to anion exchange, all proteins were dialyzed overnight against a low salt buffer (10–50 mM NaCl, 20 mM TRIS [pH = 8.0], 10% glycerol and 1 mM 1,4-dithiothreitol [DTT]) using a Servapor MWCO 12,000–14,000 membrane. Proteins loaded onto the column were eluted using a salt gradient up to 1 M NaCl over 30 min. Purity of proteins was assessed by SDS-PAGE and final samples were typically found to have >95% purity (see Supplementary figs. 85, 86). The protein aliquots were snap-frozen in liquid nitrogen and stored in −80 °C. All proteins, except for the GST-tagged tyrosine kinase domain of insulin receptor (InsR: G989-S1382) were produced with these methods. The latter was ordered from SinoBiological (Catalog number 11081-H20B1) and tested for kinase activity before use.

### Cell-based IRS1 phosphorylation assays and western blots

HEK293T cells (#CRL-3216 ATCC, Manassas, VA USA) were maintained in DMEM (#41966029 Gibco, Waltham MA USA) supplemented with 10% FBS (#10500064 Gibco), 1% penicillin-streptomycin (#P0781-100ML Sigma, St. Louis MI USA) and 0.1% Amphotericin B (#15290026 Gibco). Cells were plated into 24-well plates in 7.5 × 10^4^/well concentration and co-transfected with wild-type or Ser/Ala mutant pcDNA3.1-IRS1-FLAG and constitutive active pcDNA3.1-SHP2 plasmids using Lipofectamine 3000 (#L3000008 Invitrogen) reagent following the manufacturer’s instruction. After overnight serum-starvation cells were treated with 1 µM insulin (I9278, Sigma) for 0–30 min and harvested in Western sample buffer. Samples were run in 4–20% gradient gel (#4561096 BioRad, Hercules, CA USA) and transferred to nitrocellulose membrane using Transblot-Turbo System (#1704271 BioRad). Membranes were incubated in 1:5000 M2 anti-FLAG (#1804-200UG Sigma) and 1:1000 anti-pY612-IRS1 (#44-816 G Invitrogen) overnight and labelled with 1:10000 IRDye anti- mouse 680 (#92668070 LICOR Biosciences, Lincoln, NE USA) and 1:10.000 IRDye anti- rabbit 800 1:5.000 (#92632211 LICOR Biosciences) secondary antibodies. Membranes were scanned using the Odyssey CLx Fluorescence Imaging System (LICOR Biosciences, v5.2.5.).

### Peptide synthesis

Peptides used in this study were obtained as follows: The p0IRS1 (GRKGSGD{pTyr}MPMSPKS), ppIRS1 (GRKGSGD{pTyr}MPM(pSer)PKS), 0pRev-IRS1 (HPPEPK{pSer}PGEYVNIEFGS), ppCD28 (RSRLLHSD{pTyr}MNM{pThr}PRR), p0CD28 (RSRLLHSD{pTyr}MNMTPRR), ppSRev-IRS1 (EPK{pSer}PGE{pTyr}VNIEF), p0SRev-IRS1 (EPKSPGE{pTyr}VNIEF) peptides as well as the C-terminally lysine-ε-carboxyfluorescein-labelled CF-ppIRS1 peptide were ordered from GeneScript Inc. The 00IRS1 (GRKGSGDYMPMSPKS), 0pIRS1 (GRKGSGDYMPM{pSer}PKS) and 00Rev-IRS1 (HPPEPKSPGEYVNIEFGS) peptides were synthesized in-house, on Rink Amid resin, with a PS3 peptide synthesizer, using the Fmoc/tBu strategy. Subsequently, these peptides were purified by RP-HPLC using a Jupiter 300 Å C_18_ column (*Phenomenex*). The quality of peptides was monitored by HPLC-MS (Shimadzu LCMS-2020) and validated by mass spectrometry. The p0Rev-IRS1 (HPPEPKSPGE{pTyr}VNIEFGS) and ppRev-IRS1 (HPPEPK{pSer}PGE{pTyr}VNIEFGS) peptides (used for dephosphorylation assays only) were prepared in situ from the Rev-00IRS1 and Rev-0pIRS1 peptides, reacting them with purified, recombinant InsR tyrosine kinase. Briefly, 200 µM peptide was added to the reaction mix (100 mM NaCl, 50 mM TRIS [pH = 8.0], 5% glycerol, 0.05% IGEPAL, 2 mM DTT, 5 mM MgCl_2_, 1 mM ATP) with 2 ul Insulin receptor kinase, and incubated for 2 h before heat inactivation (10 min) at 98 °C (completion of reaction was verified by capillary electrophoresis).

### Flourescence polarizations and kinetic assays

Fluorescence polarization (FP) assays were performed as follows: In direct titration experiments, protein concentration was varied in an 1/2 or 2/3 dilution series (90 µl protein+ 45 µl mix), while concentration of the reporter peptide (CF-ppIRS1) was kept constant at 100 nM. In competitive titrations, the labelled CF-ppIRS1 peptide and protein were both fixed (at 50–80% saturation) and the unlabelled competitor peptide concentration was varied. Fluorescence polarization was always measured in triplicates, on black Corning low volume round bottom 384-well plates (with 10 ul volumes), using a BioTek Cytatation 5 reader, with the green fluorescent protein (GFP) cube filter set (ex: 485/20, em: 528/20, gain: 50, height: 7.5). Fluorescence polarization data was evaluated on Origin 2018. The dissociation constants (Kd) were determined by using the appropriate quadratic and cubic equations inserted as a user-defined function and applying nonlinear fit until convergence. The fitting formulae are shown at Supplementary note 1. Kd ratio distributions for Fig. 6 were calculated using a Monte-Carlo approach (Supplementary note 2).

For measurement of Michaelis-Menten kinetic parameters, the small-molecule fluorogenic substrate 8-difluoro−4-methylumbelliferyl phosphate (DIFMUP, Invitrogen) was utilized in serial dilusions from 100uM in H_2_O. SHP2(WT) catalytic domain or its H426A (HA),D425A (DA), K364E (Loopmut), R362E (Loopinv) or K364S + R362G (Loopless) mutants or the active version of the crystallization-optimized construct (Cryst) were applied at 2.5 nM, or 12.5 nM (for DA only) end concentrations respectively, in a phosphatase buffer (50 mM TRIS, 150 mM NaCl, 1 mM EDTA and 2 mM DTT at end concentration; pH 7.4, also containing BSA). To detect the fluorescence of the product, we used an EnSpire multimode plate reader (Perkin Elmer Inc.) with black Corning low volume 384-well plates, setting excitation wavelength to 358 and detection to 455 nm. Monitoring of fluorescence started immediately upon substrate addition, with a reading interval of 10 s, deriving the average slope from linear regression fits to at least 30 initial pointsof each curve (in triplicates). Data points were fit using Origin 2018 with standard functions, adjusted for the enzyme concentrations.

### Capillary electrophoresis assays

Phosphorylation and dephosphorylation assays with peptides were performed using capillary electrophoresis on an Agilent Capillary Electrophoresis ^3D^CE system (Agilent Technologies, Waldbronn, Germany) applying DB-WAX coated silica capillary having a 33.5 cm total and 25 cm effective length with 50 μm I.D. (Agilent Technologies, Santa Clara, CA, USA). On-line absorption at 200 nm was monitored by DAD UV-Vis detector. The capillary was thermostated at 25 °C. Before measurements the capillary was rinsed subsequently with distilled water for 15 min and between measurements with BGE (100 mM trimethylamine-phosphate buffer (pH 2.5)) for 3 min. Samples were injected by 5 × 103 Pa pressure for 6 s. Runs were performed in the positive-polarity mode with 20 kV. Phosphatase reactions were done in 50 mM TRIS, 150 mM NaCl, 1 mM EDTA and 2 mM DTT; and IGEPAL (0.1%), pH 7.4. The reaction was initialized by addition of the phosphatase enzyme. Kinase reactions were realized with the following buffer: 20 mM potassium phosphate (monobasic), 15 mM sodium phosphate (dibasic), 100 mM NaCl, 5 mM MgCl_2_, 5% glycerol, 0,05% non-ionic detergent IGEPAL, pH 7.5; reaction was started with the addition of 1 mM of ATP. Sampling from reactions was done in real time, except when conditions required pre-concentration of samples (with PTP1B only). In the latter case, reactions were run in 15 ml Falcon tubes, using an NH_4_HCO_3_-based buffer (100 mM end concentration), with 5 nM enzyme. After heat inactivation (10 min at 75 °C), the latter samples were lyophilized until dry and reconstituted in 40 ul distilled H_2_O. Kinetic curves in the article are always presented with exponential fits (that approximate the integrated Michaelis-Menten functions decently on the measured time intervals).

### Crystallization and X-ray structure determination

After several unsuccessful crystallization attempts with the native SHP2 phosphatase domain (219–528, C459S from UniProt Q06124-2), we decided to optimize the construct for crystallization. Helical segment Thr219 to Gln245 (TRINAAEIESRVRELSKLAETTDKVKQ) was removed entirely and the highly flexible loop from Glu315 to Pro323 (ETKCNNSKP) was substituted with a short Gly-Ser linker (GSSG) to rigidify the surface. In addition, three extra amino acids (SGS) were inserted between the hexa-His tag and the phosphatase domain to facilitate cleavage with TEV protease. This optimized construct was produced in E coli using an overnight expression after induction with IPTG. Purification was done on a Ni-NTA column, using the same procedure applied for the other proteins. After TEV clevage, the protein was further purified with ion exchange (Äkta explorer, GE healthcare) on a resource Q column. Finally, a gel filtration was performed on a HiLoad 16/600 Superdex 75 column. The protein, which eluted as a single peak, was concentrated to ∼9 mg/ml and mixed with the corresponding peptides: (1) the 15aa long IRS1 pTyr632-pSer636 peptide (ppIRS1), (2) the 13 aa long IRS1 pSer892-pTyr896 peptide (ppSRev-IRS1), (3) or the 16 aa long CD28 pTyr191-pThr195 peptide (ppCD28), at a 3:2 molar excess. Crystallization was done by the vapour diffusion method, with the hanging drop technique using 0.75 M NaCl as reservoir at 4 °C temperature. Crystallization yielded rhomboid shaped crystals in PEG 20000, HEPES buffer pH 7.5 (SHP2 -ppIRS1) or citrate buffer pH 5.5 (SHP2-ppSRev-IRS1, SHP2-ppCD28) and 50 mM EDTA glycerol 20% (end concentration) was applied to harvested crystals before flash freezing them on liquid N_2_. X-ray diffraction data was collected at Hamburg (EMBL PETRA III beamlines) on several crystals, and the best diffraction datasets were selected for each complex. -ray data sets were processed and scaled using XDS^47^ (version Jan 26, 2018) and Aimless^48^ (0.6.2), respectively. Crystal structures were solved using the molecular replacement method and the program Phaser^49^ (3.19) with the catalytic domain of human SHP2 (PDB ID: 3ZM0^50^) as a search model. Refinement was done with PHENIX^51^ (1.17.1-3660) while model building and correction was carried out with Coot^52^. (0.8.9.2) The presence of phosphopeptides is supported by the Fo-Fc omit maps at 1.5 sigma level, see Supplementary fig. 17.

### In silico modelling of SHP2-substrate complexes

For the purpose of in silico modelling, 20 substantially different initial models (for each structure) were constructed from the experimental X-ray structures using Coot and PyMOL. For docking, we created the following unambiguous restraints for the phosphotyrosine and the −2 Gly from 22 already published tyrosine phosphatase structures in the PDB: dist(TyrO2P-Ala461N) = 3.1 (−0.2 + 0.3), dist(TyrO1P-Ile463N) = 3.2 (−0.7 + 0.1), dist(TyrO1P-Gly464N) = 2.9 (−0.3 + 0.4), dist(TyrO3P-Arg465N) = 2.9 (−0.3 + 0.2), dist(TyrCB-38CG) = 3.8 (−0.4 + 0.1), dist(TyrCB-Ile282CG1) = 3.7 (−0.3 + 1.6), dist(GlyO-Lys280N) = 3.3 (−0.5 + 0.8), numbering as of UniProt Q06124-2. (See supplementary table 3.) For the definition of the last distance criterion, only the 19 structures showing H-bonding to the surface at the −2 position (similarly to the Gly in our SHP2 structure) were considered. These unambiguous restraints were used to drive docking of each 20-membered ensemble in Haddock 2.2 webserver with default setup. Clustering was performed based on fraction of common contact (FCC). Only the resulting best cluster was analysed further for each structure. The input models for the hypothetical catalytic state (WPD-closed models) were created by editing SHP2 with the help of homologous PTP1B catalytic complexes (PDB: 1EEN and 3I7Z, yielding 6 SHP2 models) as well as modifying the peptide substrate accordingly (yielding 6 peptide models for each complex). Using the Haddock outputs with the same ambiguous restraints as above, we eventually selected 3 different models to represent each complex.

Molecular dynamics (MD) simulations were performed using GROMACS (2018.8)^53^, with modified force field 54a8^54^ on a representative subset of lowest-energy structural ensembles (5 models for each complex) docked by Haddock^55^. Phosphorylated amino acids were converted to the appropriate format through the Vienna-PTM 2.1 server^56^. The topology files were generated by using SPC/E water model and defining charged termini (NH_3_^+^ and COO^−^, corresponding to protein termini in crystal and the exact free termini of the peptides used in experiments). The molecules were solvatated in a cube with periodic boundary and by using SPC216 model. The system was neutralized by adding Cl^−^ or Na^+^ ions. The assembled system was then relaxed by energy minimalization (800 step). We equilibrated the system both NVT and NPT in 100 ps. MD simulations were run for 10 ns with 2 fs steps. Temperature was set to 300 K. We used the gromos54a8 force filed, because the modified residue types (S2P and Y2P, both phosphates with −2 charge) were defined in this firce field. The singly and doubly phosphorylated (pre-catalytic) models used identical starting structures, save the Ser/Thr phosphate groups. Molecular dynamics videos were prepared using Pymol and converted to MPEG−4 using OpenShot (v 2.6.1).

Orthosteric inhibitors with no experimentally determined structure were docked using AutoDockTools and AutoDock Vina^36,57^. The input protein template was constructed from our SRev-ppIRS1 structure with minimal conformational modifications. 3D ligand structures were downloaded from PubChem and prepared using AutoDockTools (version 1.5.7, Scripps Institute) alongside with the protein template.

### Reporting summary

Further information on research design is available in the Nature Research Reporting Summary linked to this article.

## Supplementary information

Supplementary information

Description of supplementary datasets

Supplementary dataset 1

Supplementary dataset 2

Supplementary dataset 3

Reporting summary

## Data Availability

Source data supporting the findings of the manuscript has been included in the current article. X-ray structures were deposited to the Protein Data Bank (PDB) under entries 7PPL (ppIRS1), 7PPN (ppCD28) and 7PPM (ppSRev-IRS1), respectively. All other structures analyzed in our article are freely available in the worldwide PDB (entries 1EEN, 1EEO, 1G1F, 1G1H, 1G1G, 1LQF, 1YGU, 3D42, 3D44, 3I7Z, 3MOW, 3OLR, 3OMH, 3ZM0, 3ZMP, 4ICZ, 4GFU, 4GFV, 4NND, 4QUM, 4RDD, 4RH5, 4S0G, 4RH9, 4RHG, 4ZRT). Representative Haddock ensembles and MD simulations are also provided as supplementary data (Supplementary datasets 1, 2 and 3). Source data are provided with this paper.

## Source data

Source data

## Code availability

Beyond commercially available software packages (see methods), we utilized simple Python scripts to calculate the estimated mean and ±SD for dissociation constant proportions (Fig. 6b) and for the comparative evaluation of molecular dynamics (Supplementary figs 68–73). The source codes are provided under Supplementary notes 2 and 3.
