# Supplementary material for: Structural insights into the pSer/pThr dependent regulation of the SHP2 tyrosine phosphatase in insulin and CD28 signaling
Source: Nat Commun. 2022 Sep 16;13:5439. doi: 10.1038/s41467-022-32918-5 (PMC9481563; doi:10.1038/s41467-022-32918-5)
Supplement: Supplementary file 1 — Supplementary information [file 41467_2022_32918_MOESM1_ESM.pdf]

Supplementary information

Structural insights into the pSer/pThr dependent regulation of the SHP2 tyrosine phosphatase in insulin and CD28 signaling

András Zeke, Tamás Takács, Péter Sok, Krisztina Németh, Klára Kirsch, Péter Egri, Ádám Levente Póti, Isabel Bento, Gábor E Tusnády, Attila Reményi

Table of contents

Supplementary figures 2
Supplementary tables 80
Supplementary notes 84
Supplementary references 87

Supplementary figures

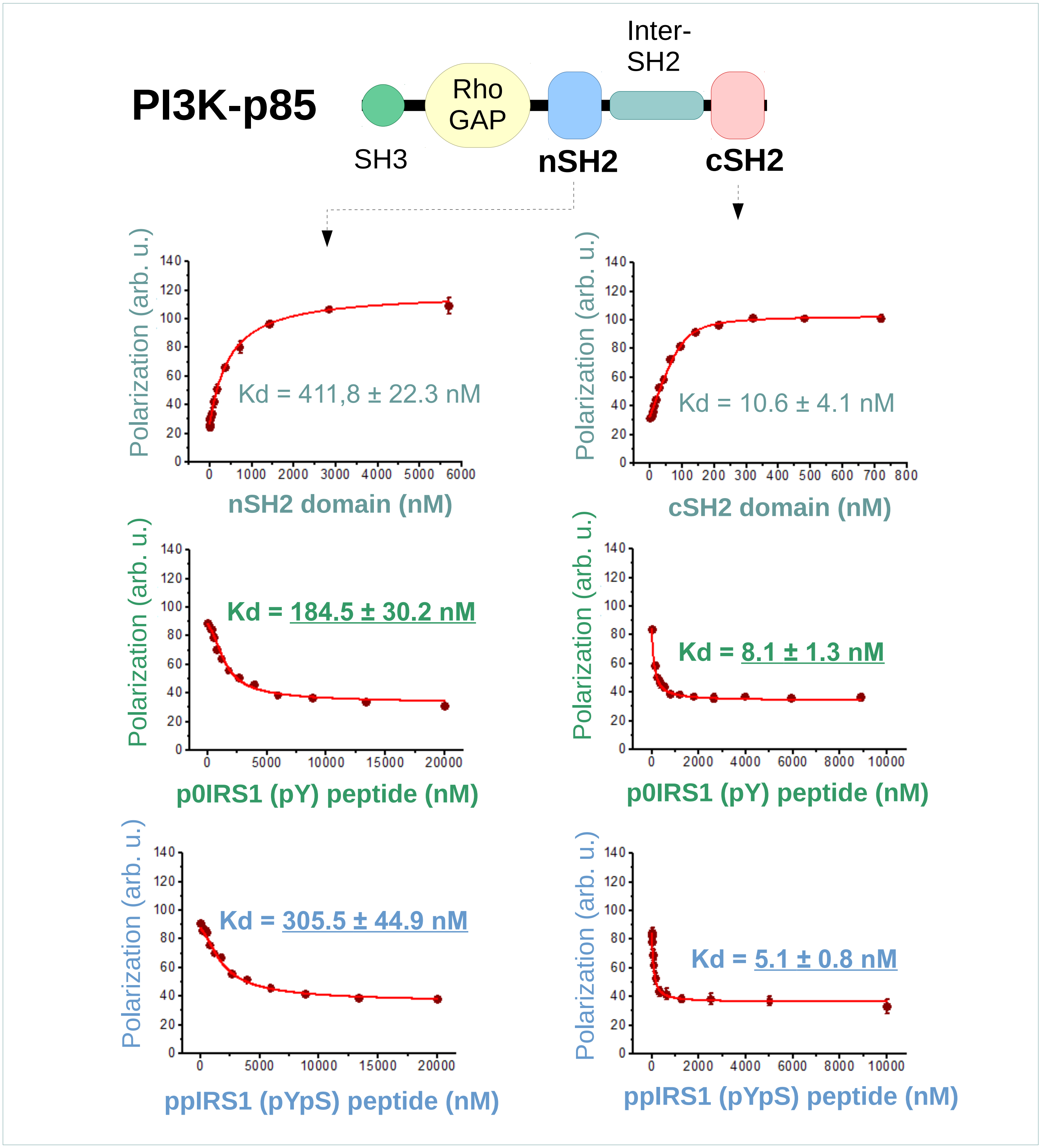

Supplementary figure 1. Fluorescence polarization assays on the isolated N- and C-terminal SH2 domains of the phosphatidyl-inositol-3-kinase (PI3K) regulatory subunit p85. Direct titrations against the carboxyfluorescein-labelled ppIRS1 peptide were followed by competitive titrations using unlabelled singly- and doubly-phosphorylated IRS1 peptides. Although the C-terminal SH2 domain binds these peptides much stronger than the N-terminal domain, the presence of the phosphoserine barely alters the dissociation constants (Kd values). Error bars refer to SD values (n=3 technical replicates for all measurements). Source data are provided as a Source Data file.

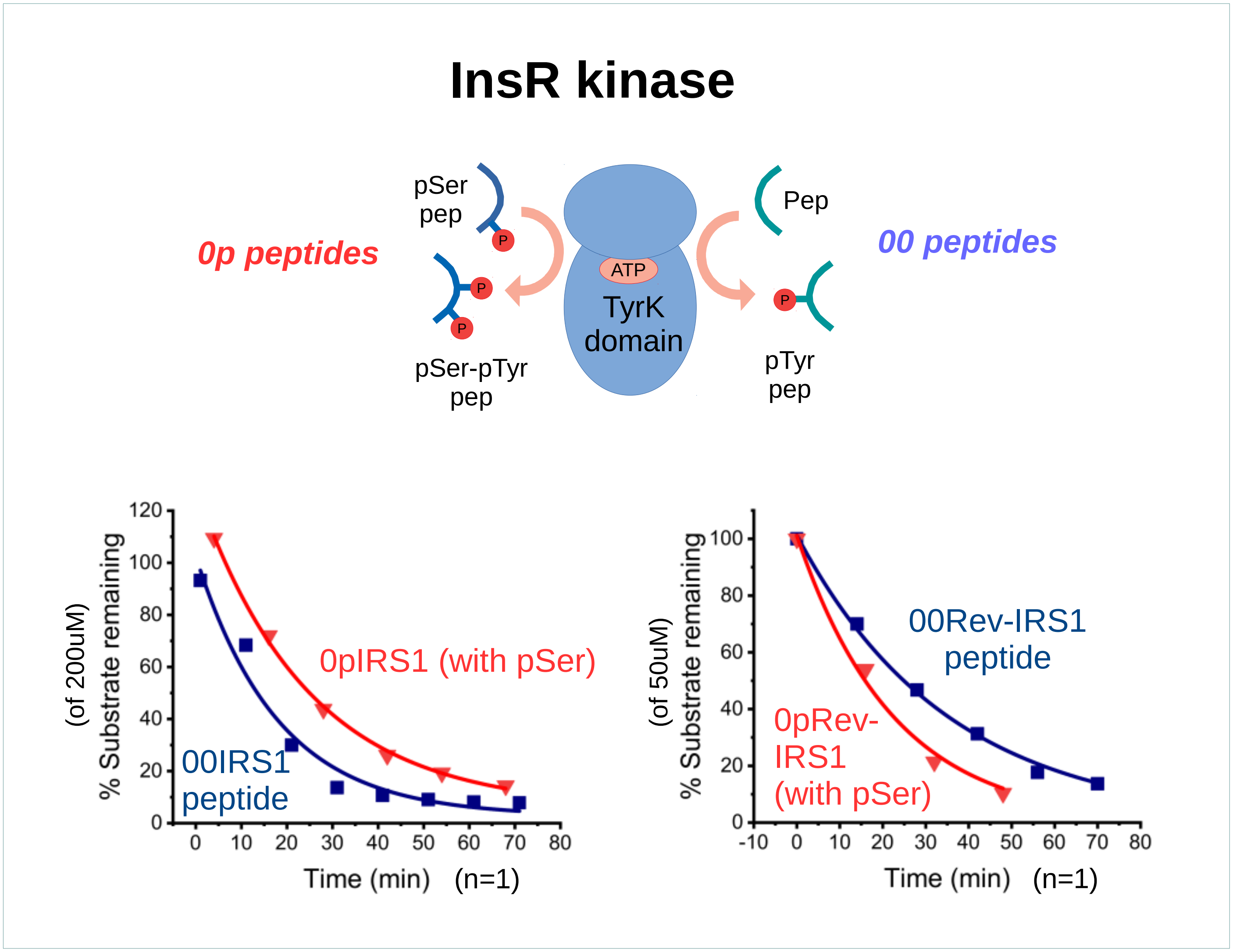

Supplementary figure 2. Phosphorylation assays using the recombinant, isolated tyrosine kinase domain of the human insulin receptor (InsR) on IRS1 peptides, with or without phosphoserines. In all experiments, the presence of a pSer resudie either at the +4 position (0pIRS1) or at the -4 position (0pRev-IRS1) altered kinetics to a very limited degree. Besides the illustration of substrate and product peptides, two representative kinatic curves are presented, showing the substrate consumption over time as monitored by a capillary electrophoresis assay. Source data are provided as a Source Data file.

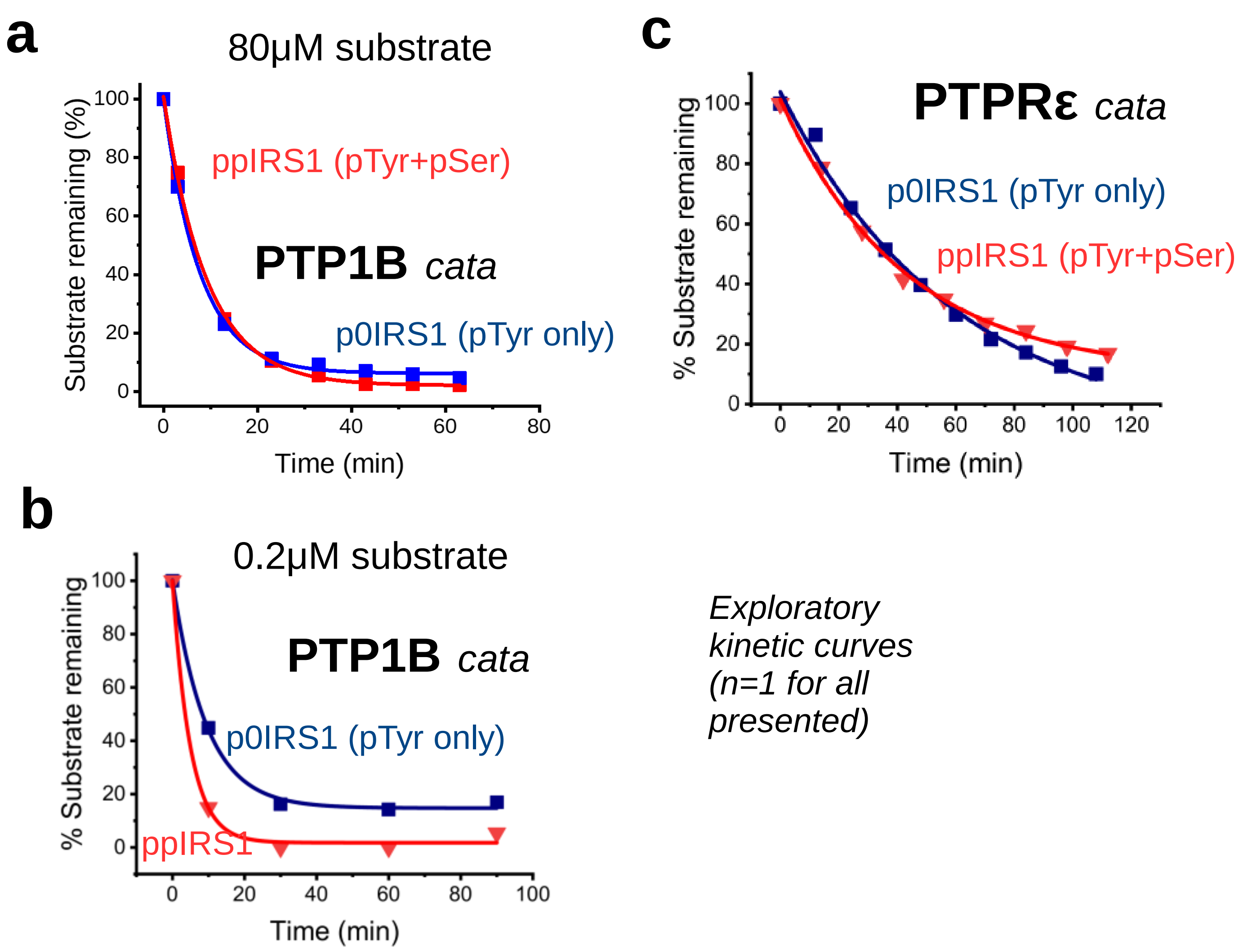

Supplementary figure 3. Capillary electrophoresis-based dephosphorylation assays using the p0IRS1 or ppIRS1 peptides on PTP1B at high substrate concentration (**a:** 6nM PTP1B + 200 nM peptide). Probing dephosphorylation by PTP1B at low substrate concentration, using lyophilization and subsequent capillary electrophoresis (**b:** 6nM PTP1B + 200 nM peptide). Dephosphorylation experiments with PTPRε (**c:** 270nM PTPRε + 80µM peptide) indicated no modulation of activity by the flanking Ser phosphorylation site. In every case, a single representative curve is shown, with simple exponential fits that approximate the integrated Michaelis-Menten kinetics. Source data are provided as a Source Data file.

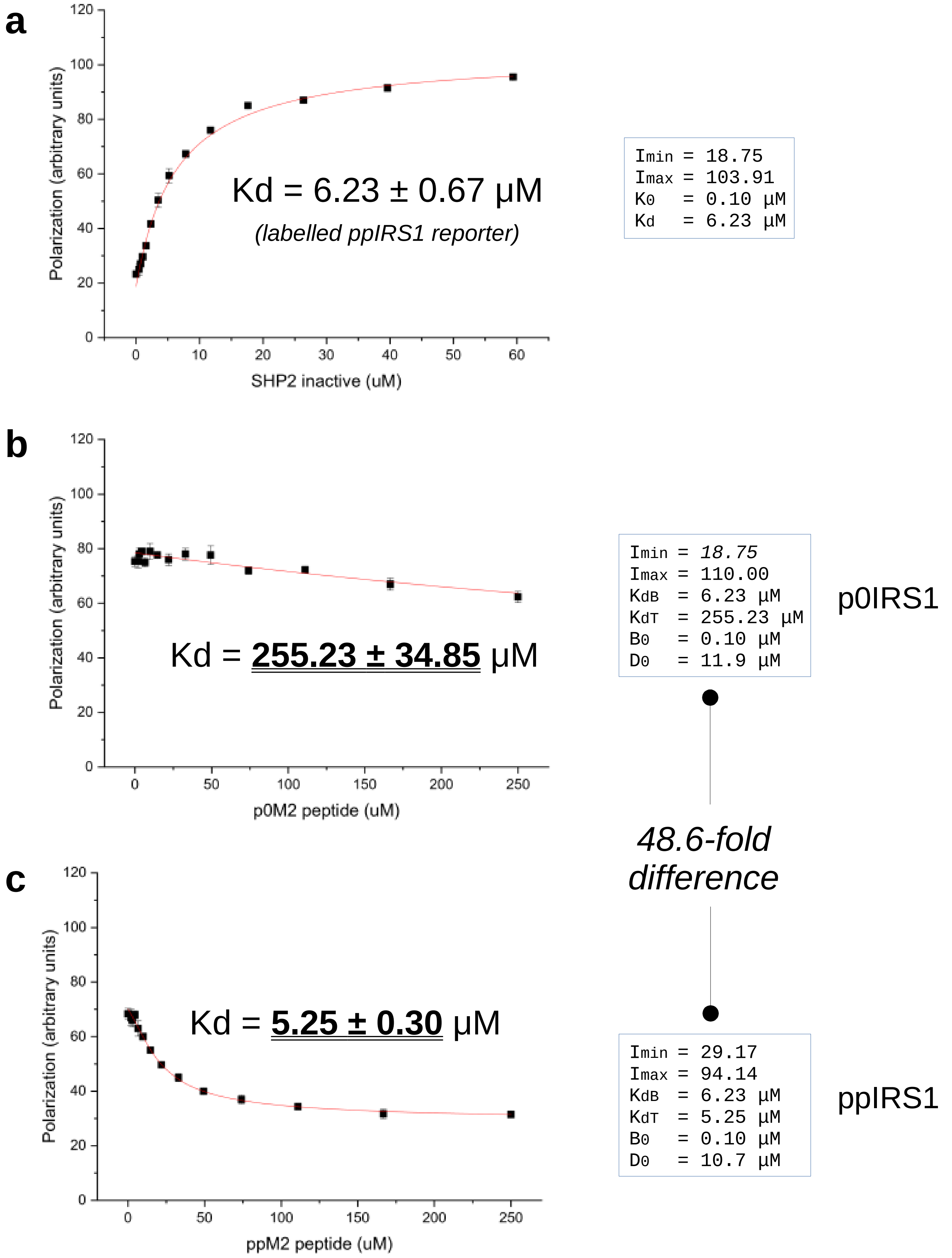

Supplementary figure 4. SHP2 direct (a) and competitive (b, c) fluorescence polarization titrations against IRS1 (=M2) peptides (n=3 technical replicates, error bars show ±SD for each point). Source data are provided as a Source Data file.

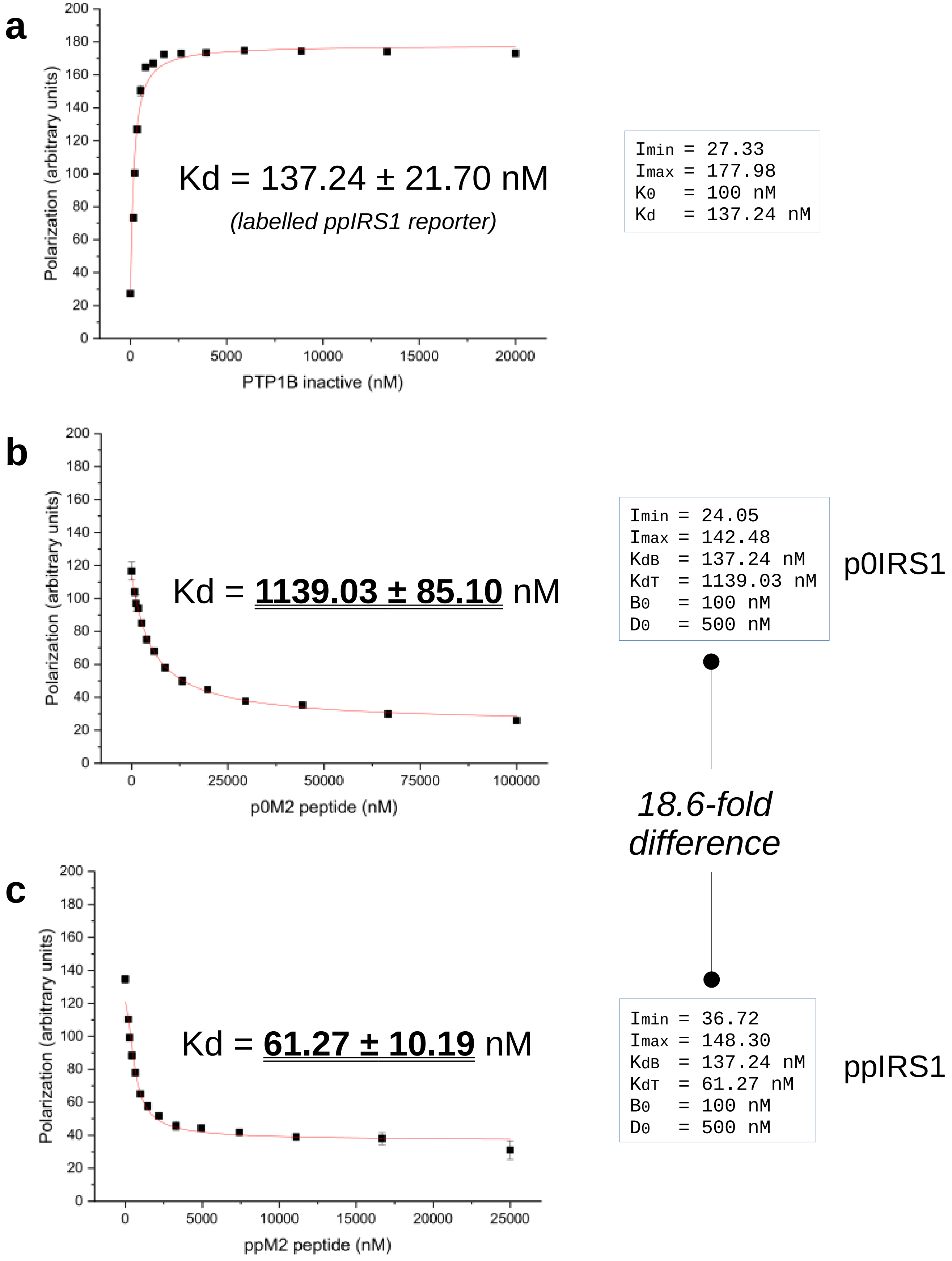

Supplementary figure 5. PTP1B direct (a) and competitive (b,c) fluorescence polarization titrations against IRS1 (=M2) peptides (n=3 technical replicates, error bars show ±SD for each point). Source data are provided as a Source Data file.

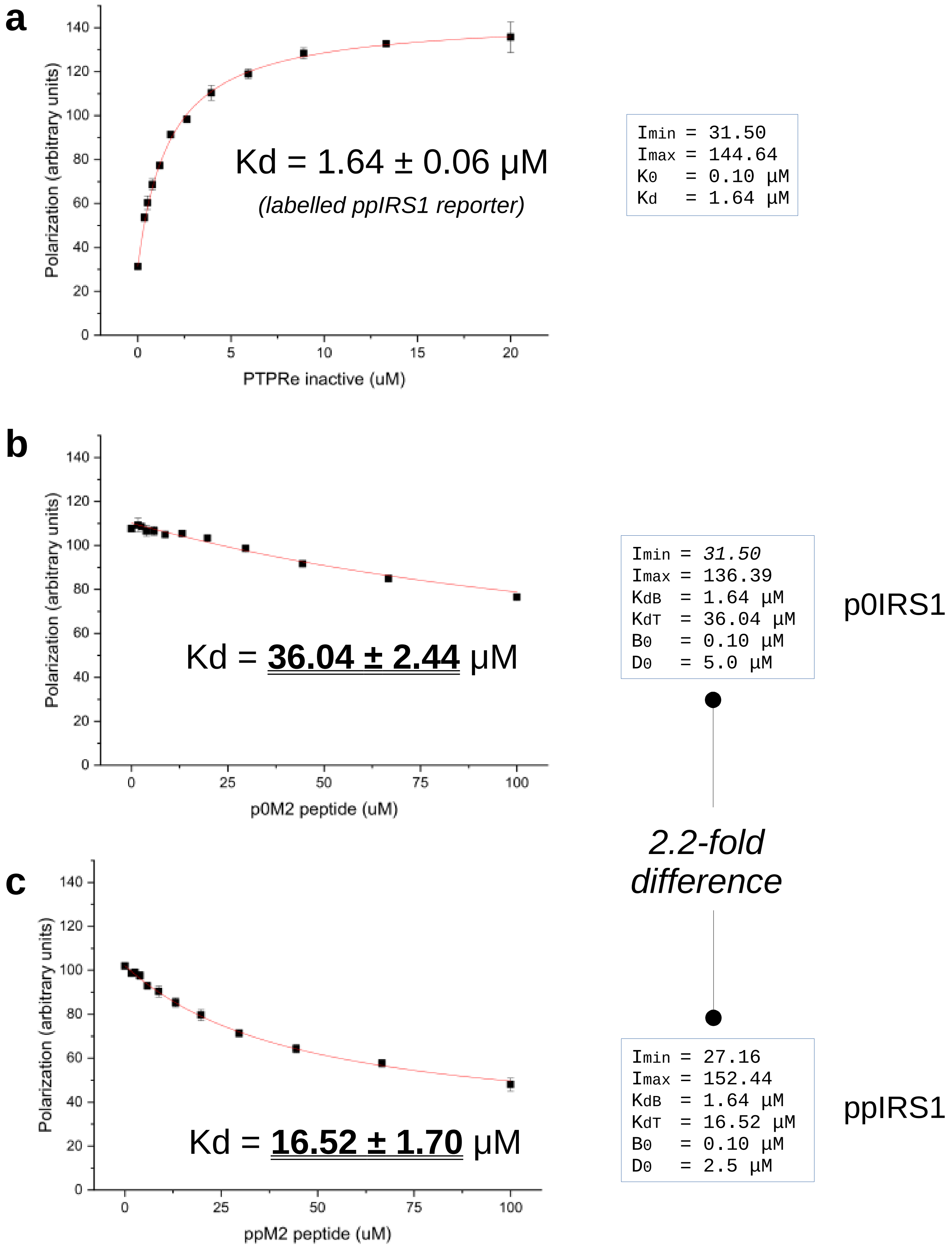

Supplementary figure 6. PTPRε direct (a) and competitive (b,c) fluorescence polarization titrations against IRS1 (=M2) peptides (n=3 technical replicates, error bars show ±SD for each point). Source data are provided as a Source Data file.

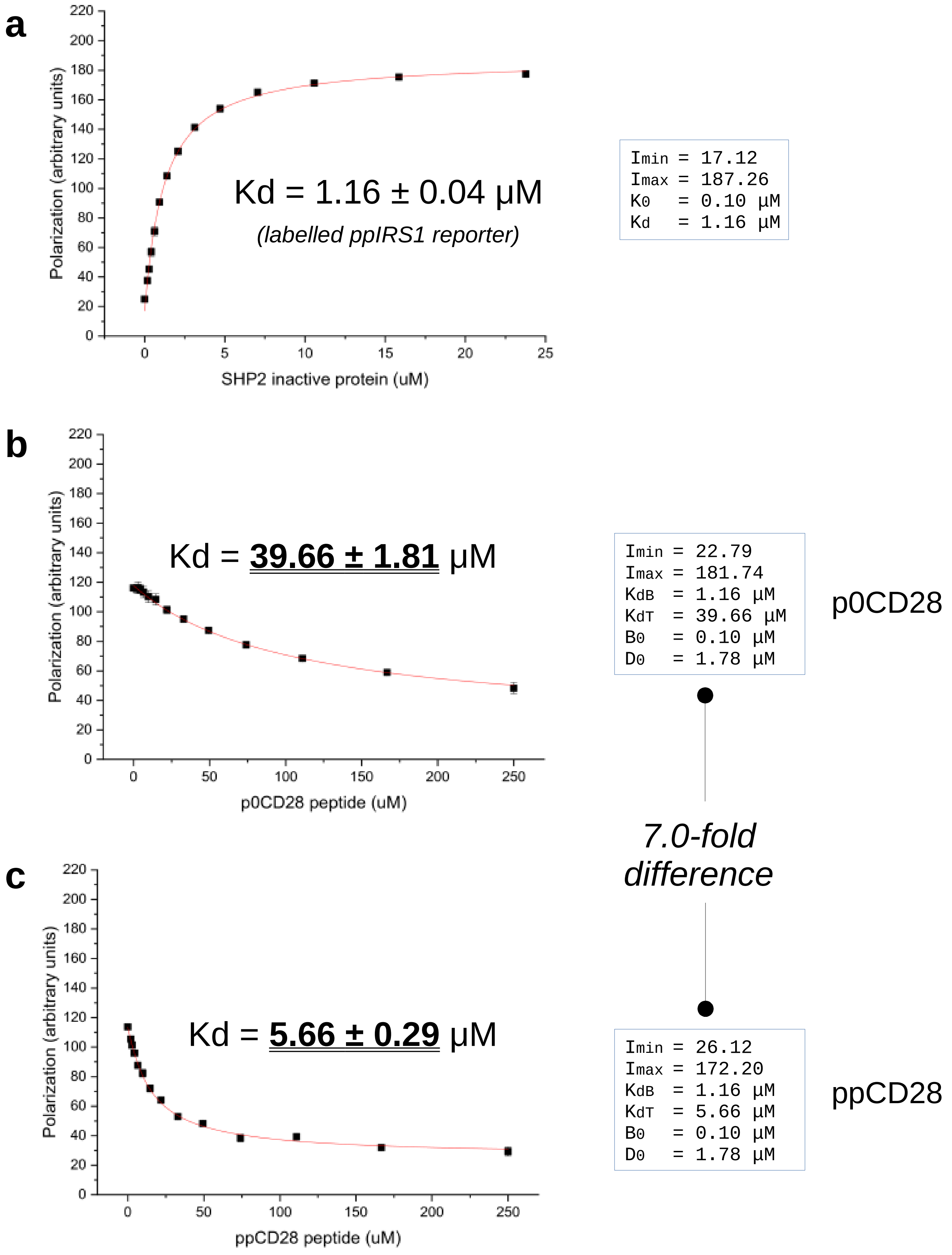

Supplementary figure 7. SHP2 direct (a) and competitive (b,c) fluorescence polarization titrations against labelled IRS1 and CD28 peptides (n=3 technical replicates, error bars show ±SD for each point). Source data are provided as a Source Data file.

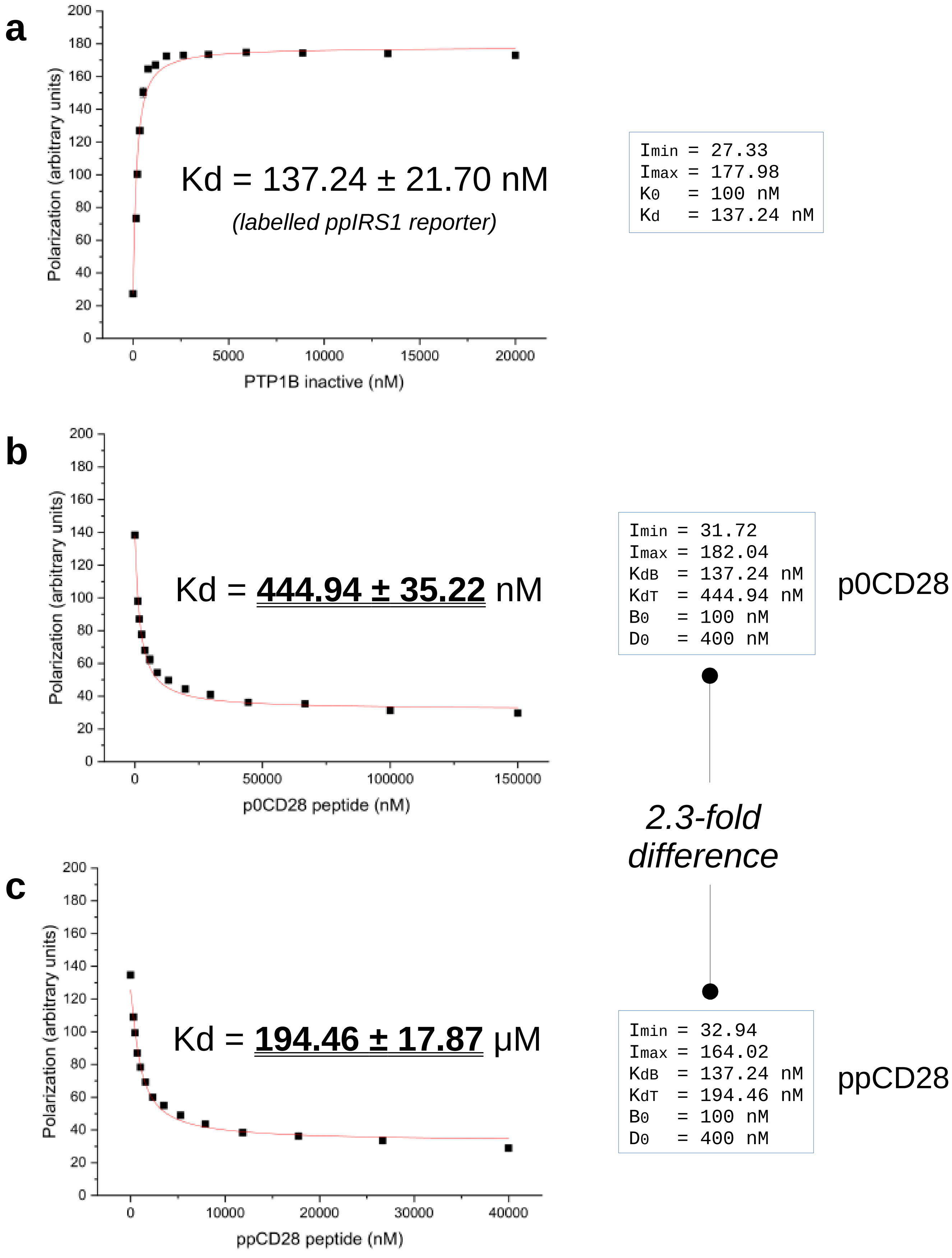

Supplementary figure 8. PTP1B direct (a) and competitive (b,c) fluorescence polarization titrations against labelled IRS1 and CD28 peptides (n=3 technical replicates, error bars show ±SD for each point). Source data are provided as a Source Data file.

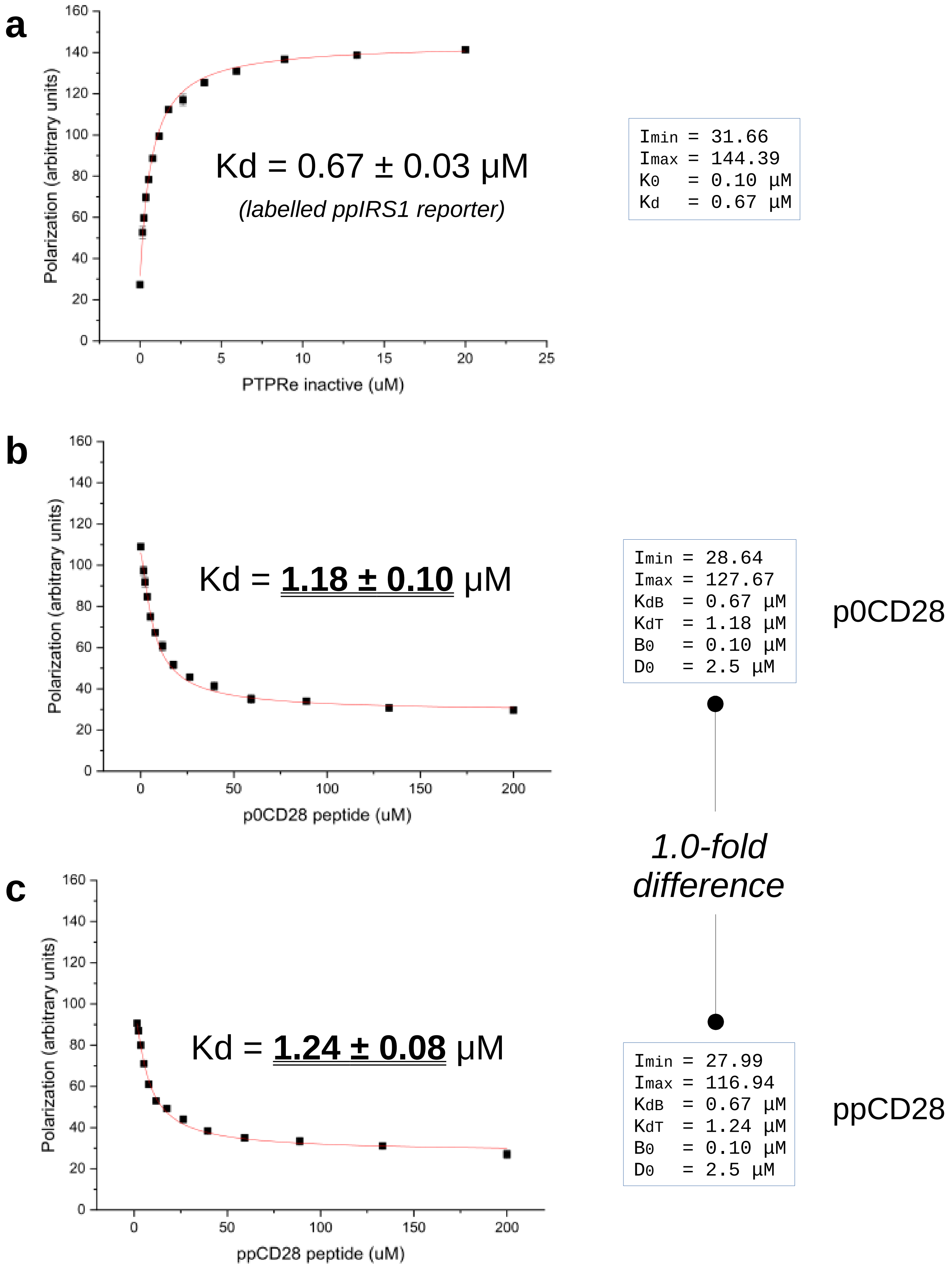

Supplementary figure 9. PTPRε direct (a) and competitive (b,c) fluorescence polarization titrations against labelled IRS1 and CD28 peptides (n=3 technical replicates, error bars show ±SD for each point). Source data are provided as a Source Data file.

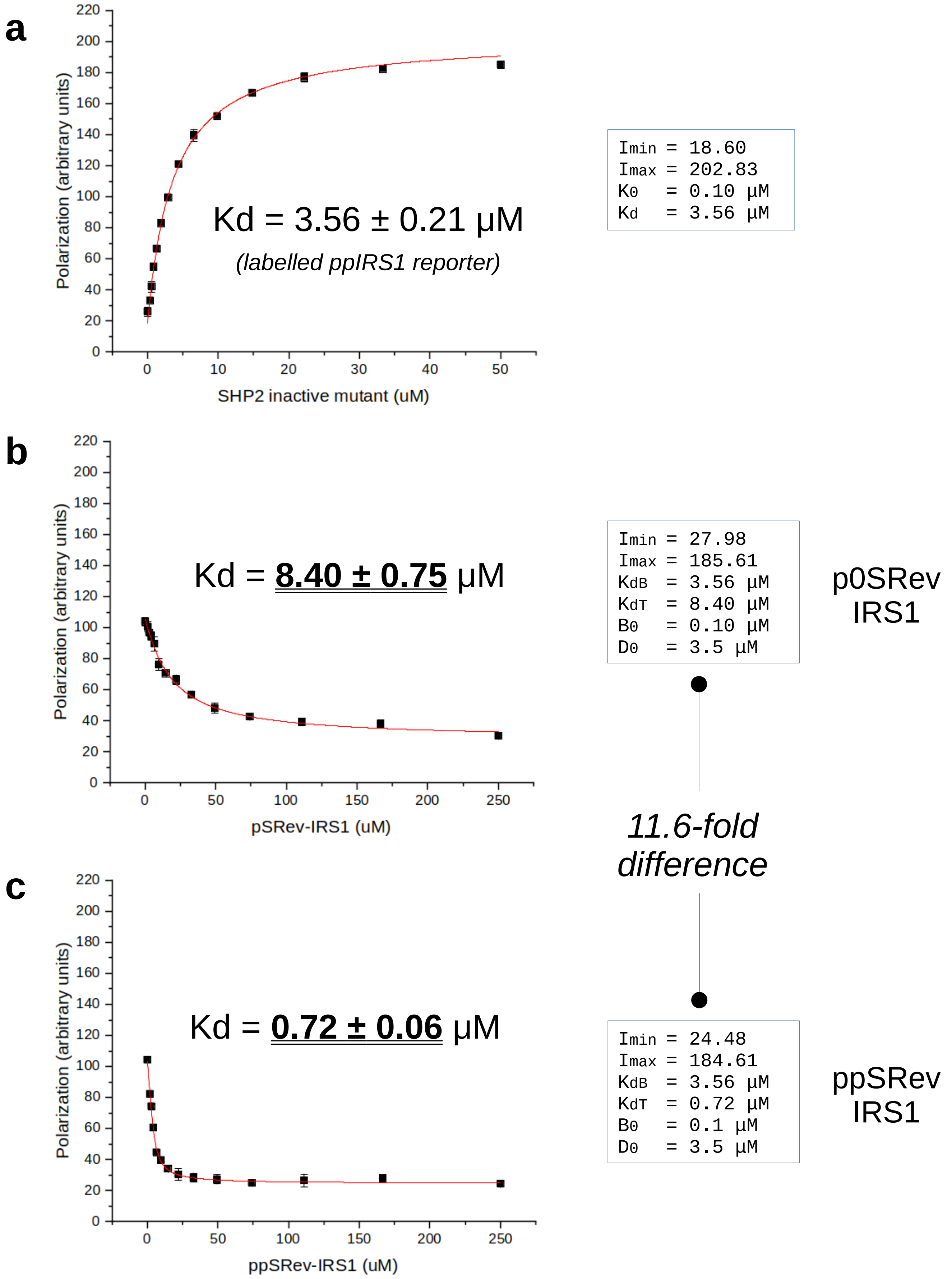

Supplementary figure 10. SHP2 direct (a) and competitive (b,c) fluorescence polarization titrations against labelled IRS1 and short reverse (SR) IRS1 peptides (n=3 technical replicates, error bars show ±SD for each point). Source data are provided as a Source Data file.

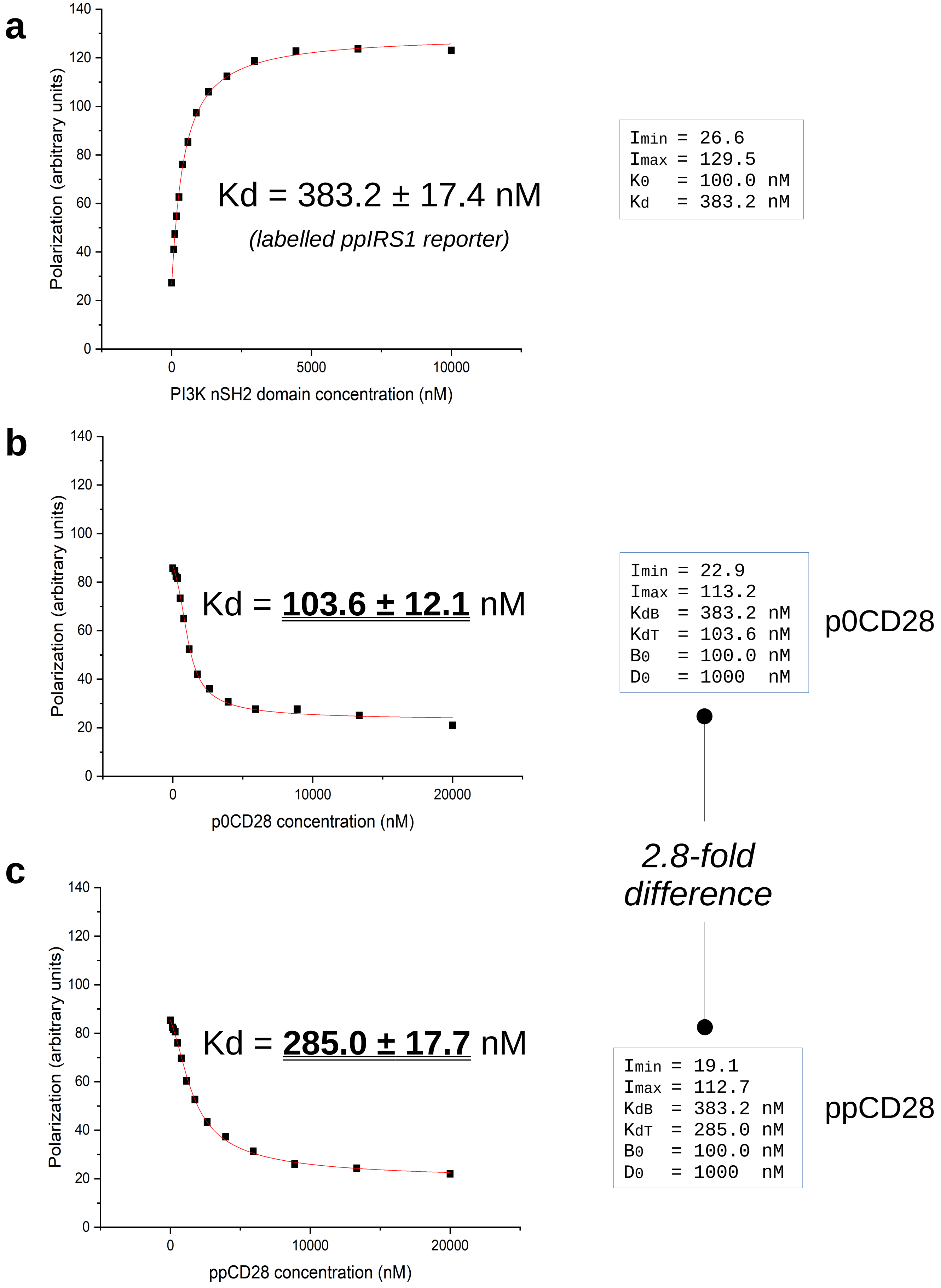

Supplementary figure 11. PI3K N-terminal SH2 domain direct (a) and competitive (b,c) fluorescence polarization titrations against CD28 peptides (n=3 technical replicates, error bars show ±SD for each point). Source data are provided as a Source Data file.

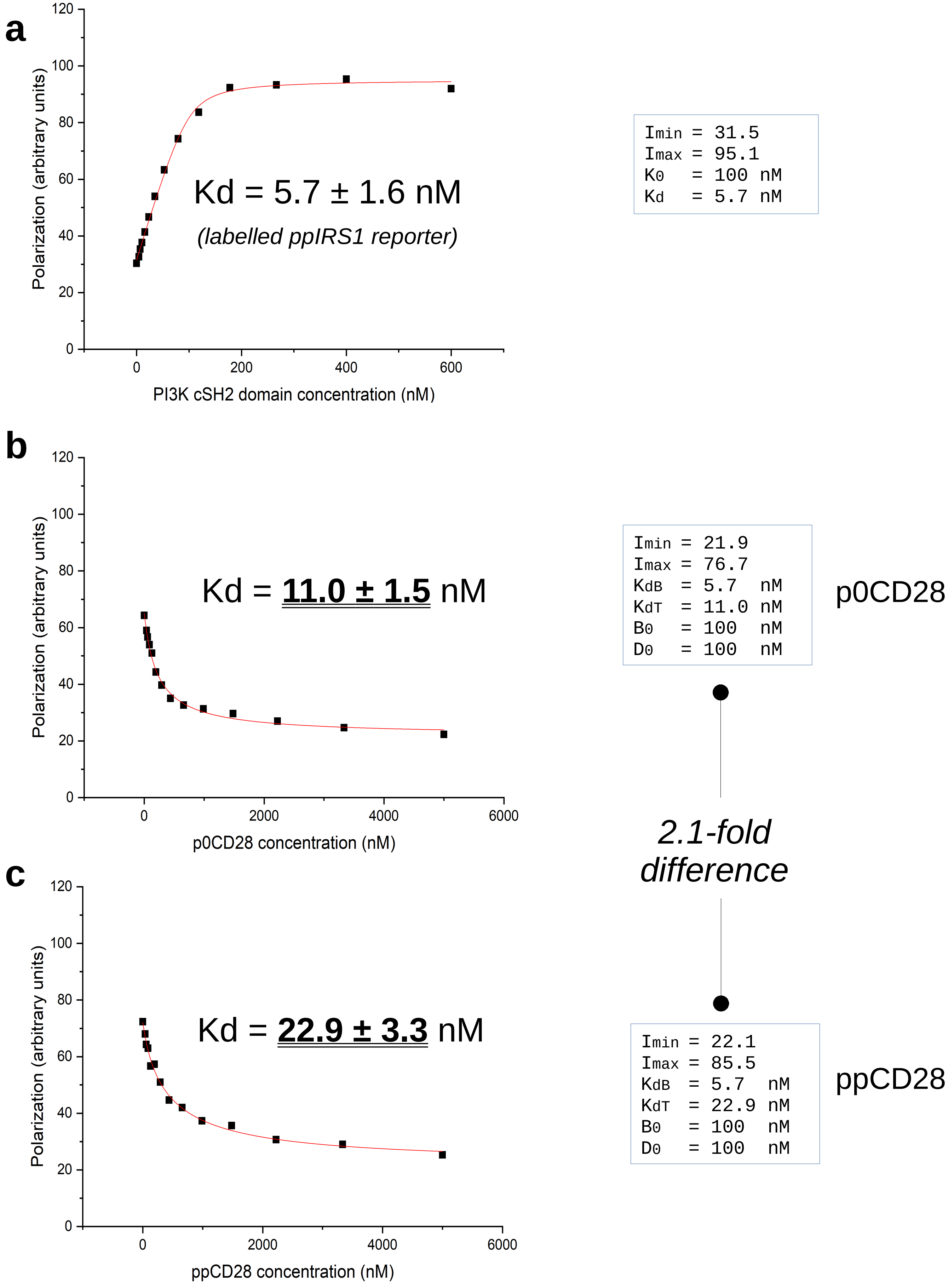

Supplementary figure 12. PI3K C-terminal SH2 domain direct (a) and competitive (b,c) fluorescence polarization titrations against CD28 peptides (n=3 technical replicates, error bars show ±SD for each point). Source data are provided as a Source Data file.

a. Baseline trend analysis (all pooled experiments)

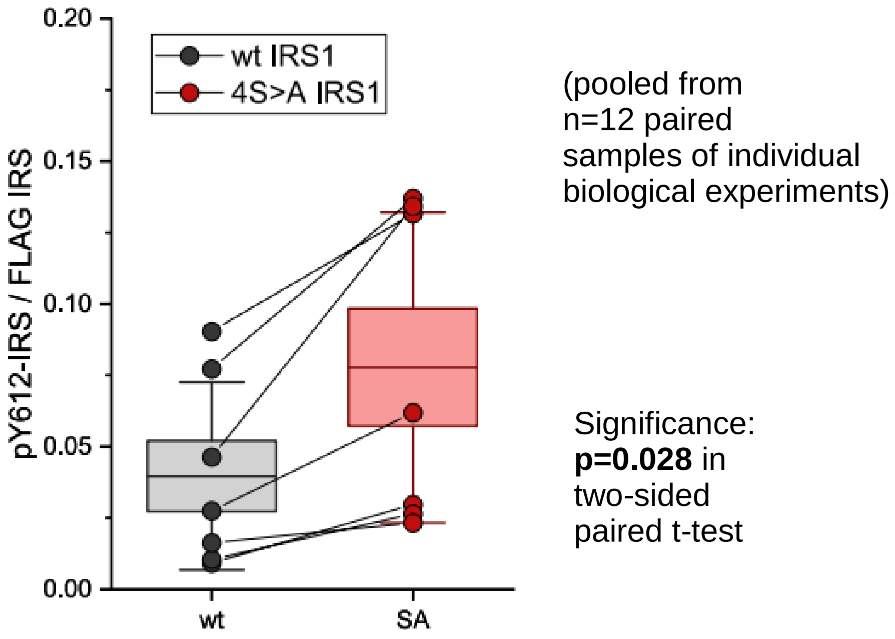

b. Time couse analysis (all pooled experiments)

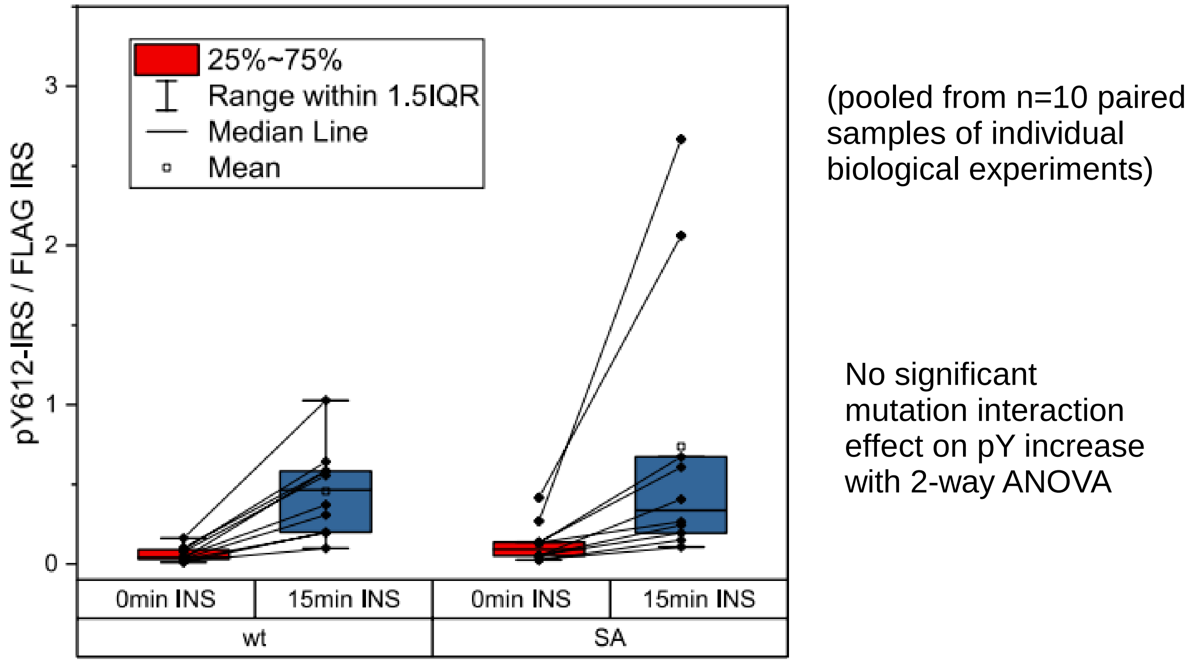

Supplementary figure 13. Summary of all western blot analyses: Insulin stimulation baseline (a) and time-dependent kinetics (b) with wild-type IRS1 and IRS1-4SA lacking +4 flanking phosphorylation sites). The analyses show box and whiskers plots of the paired samples, displaying lower and upper quartiles, medians and the ±1.5 interquartile ranges as whiskers. Source data are provided as a Source Data file.

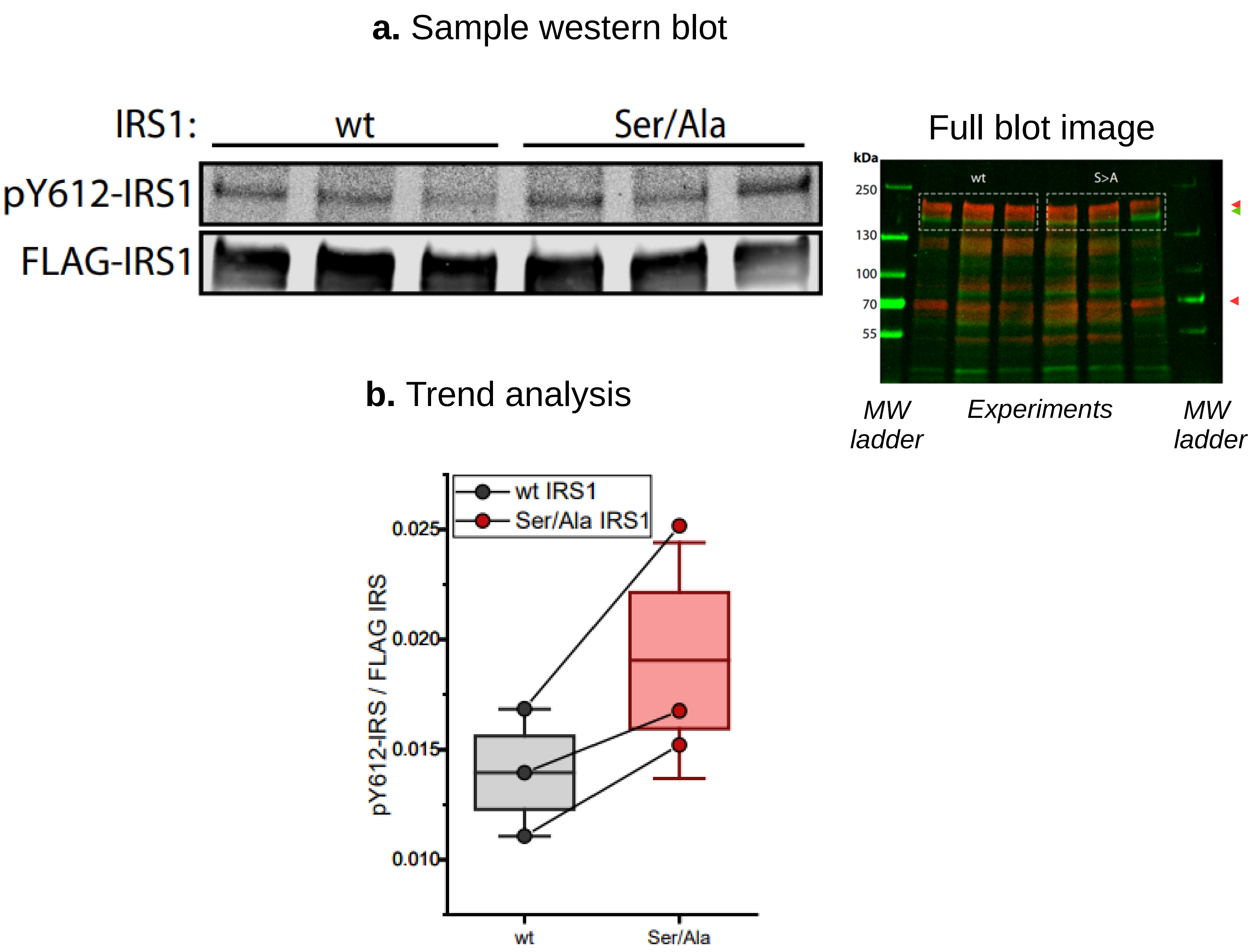

(n=3 biologically independent experiments)

Supplementary figure 14. Sample western blot (a) showing that the baseline IRS1 Tyr phosphorylation at site Y612 is consistently enhanced in the mutant lacking flanking +4 Ser phosphorylation sites. On the full blot inset, the red channel indicates anti-FLAG and the green channel the anti-pY612 intensity as well as the marker bands. Small arrows show the position of the FLAG-IRS1, pY612-IRS1 and FLAG-SHP2 bands. The trend analysis (b) shows a box and whiskers plot of the paired samples, displaying lower and upper quartiles, median and the ±1.5 interquartile range as whiskers. Source data are provided as a Source Data file.

a. Sample western blot

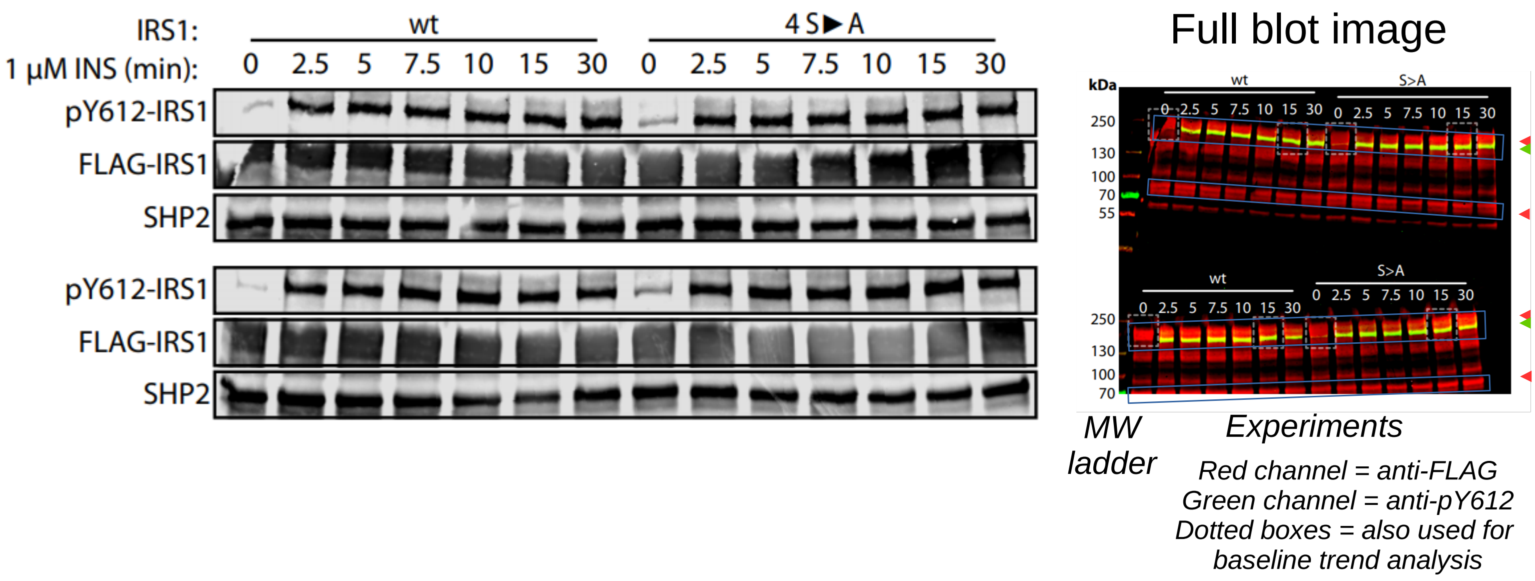

b. Time course (n=2 biologically independent experiments)

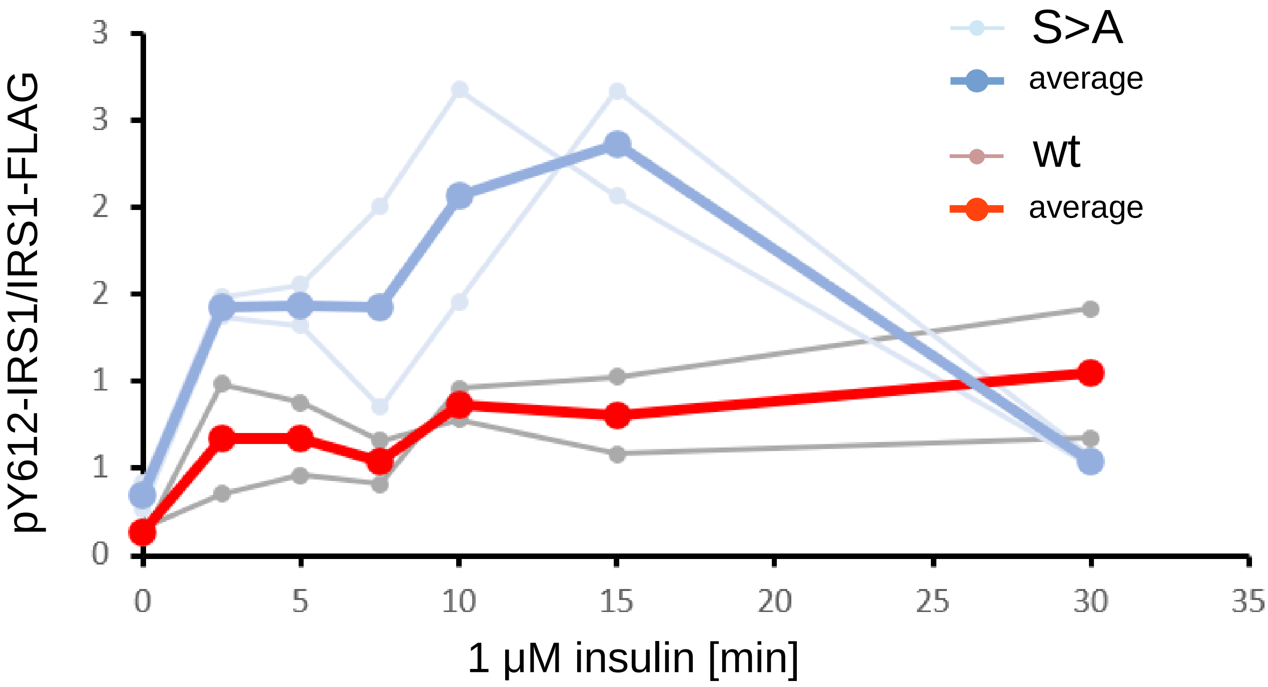

Supplementary figure 15. Sample kinetic experiments (a) showing mild, but highly variable and non-significant changes of IRS1 Y612 tyrosine phosphorylation when lacking flanking phosphosites. On the full blot inset, the red channel indicates anti-FLAG and the green channel the anti-pY612 intensity. Small arrows indicate the position of the FLAG-IRS1, pY612-IRS1 and FLAG-SHP2 bands. On the graph (b), thin lines show values for individual experiments, while their average is shown by the thick lines. Source data are provided as a Source Data file.

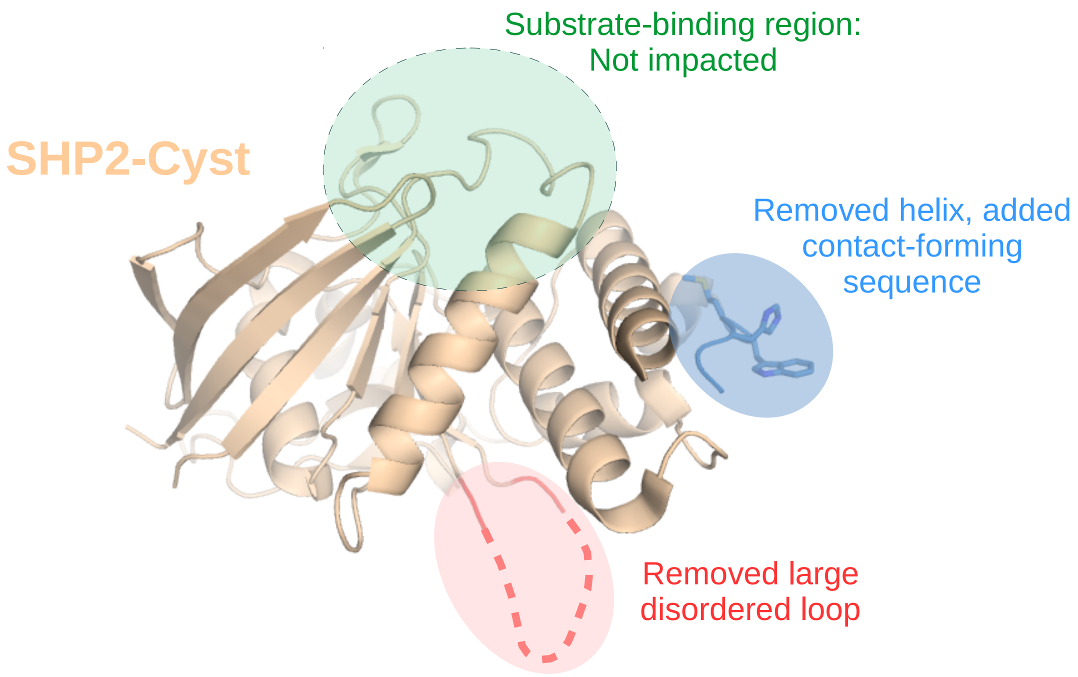

Supplementary figure 16. Design principles of the SHP2 crystallization construct

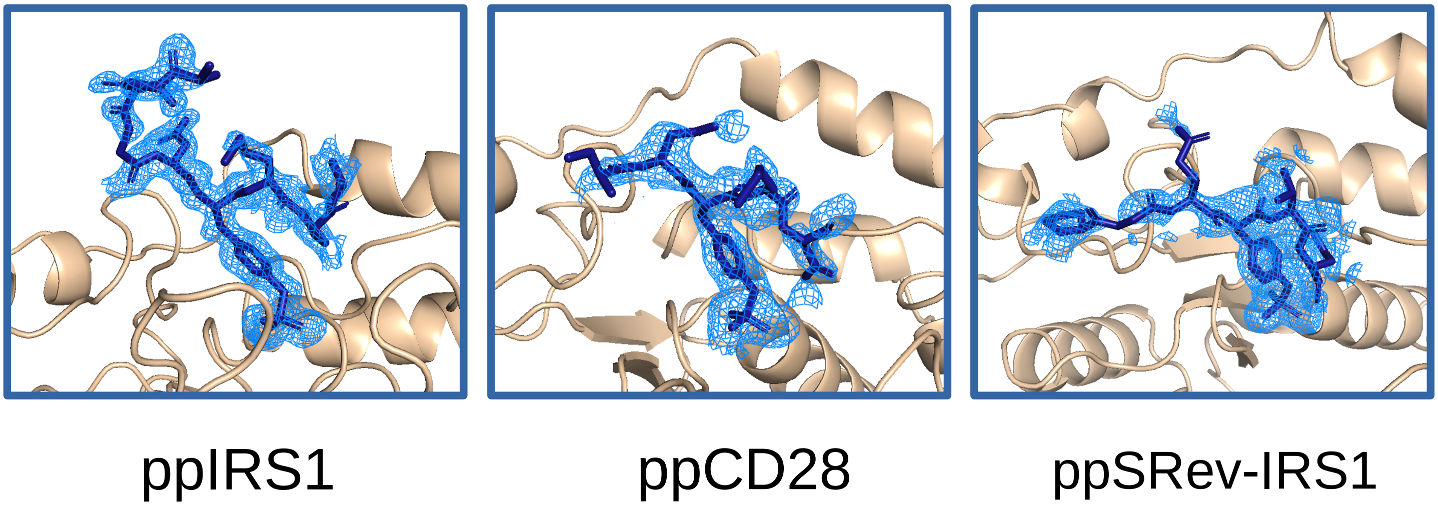

Supplementary figure 17. Fo - Fc omit electron density maps for the doubly phosphorylated peptides binding to SHP2 at 1.5 sigma level.

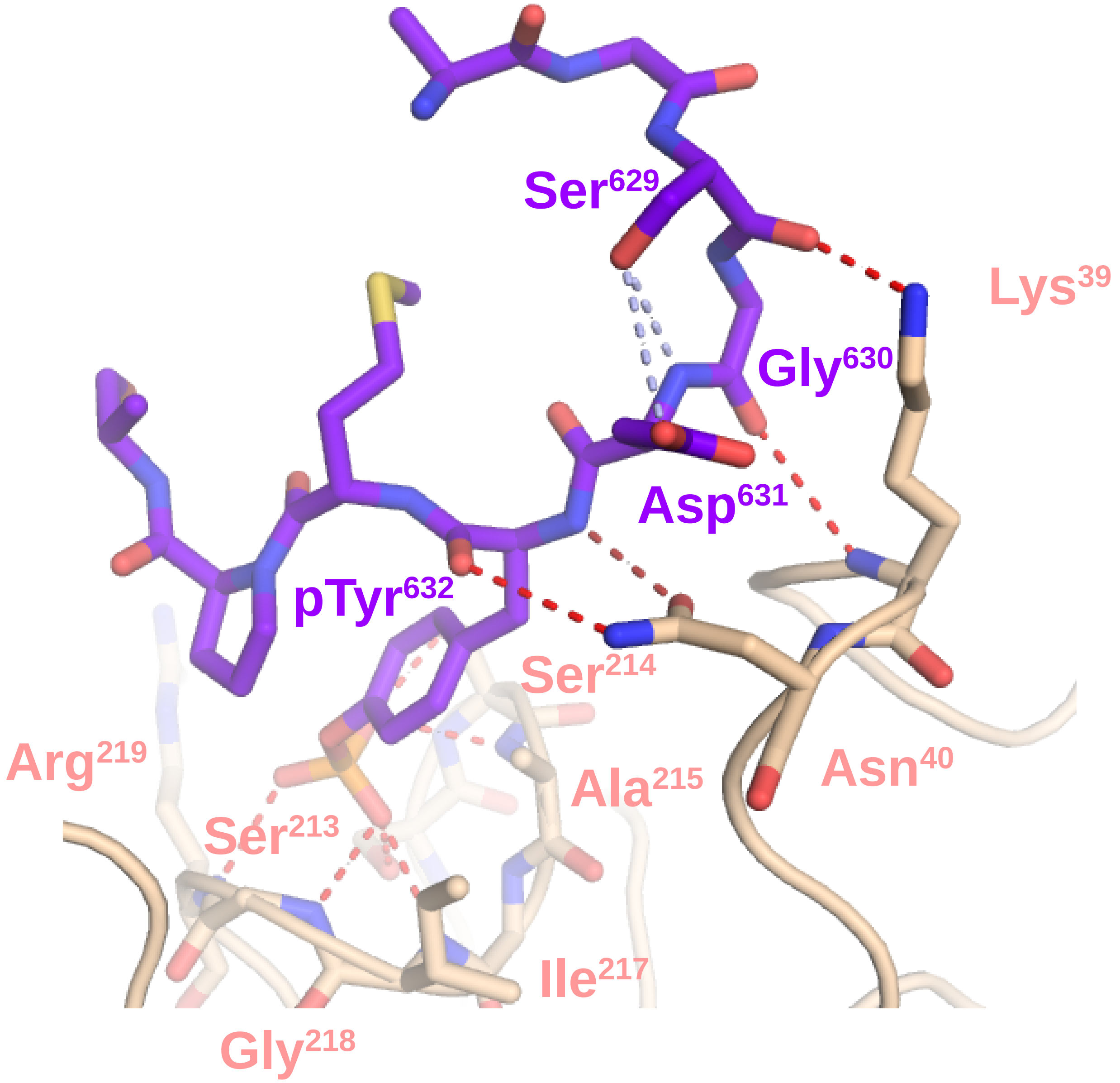

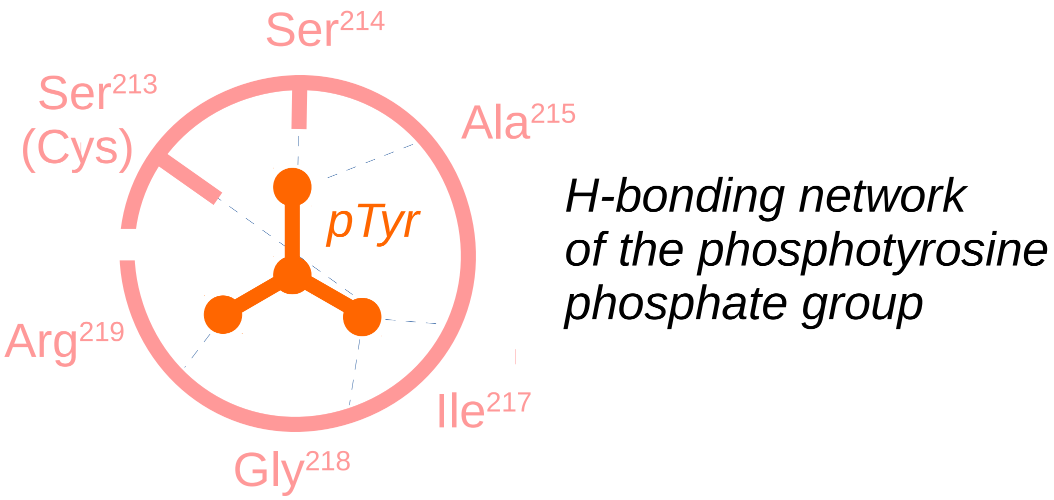

Supplementary figure 18. Inter-chain (red) and intra-chain (blue) polar contacts observed in the SHP2-ppIRS1 structure

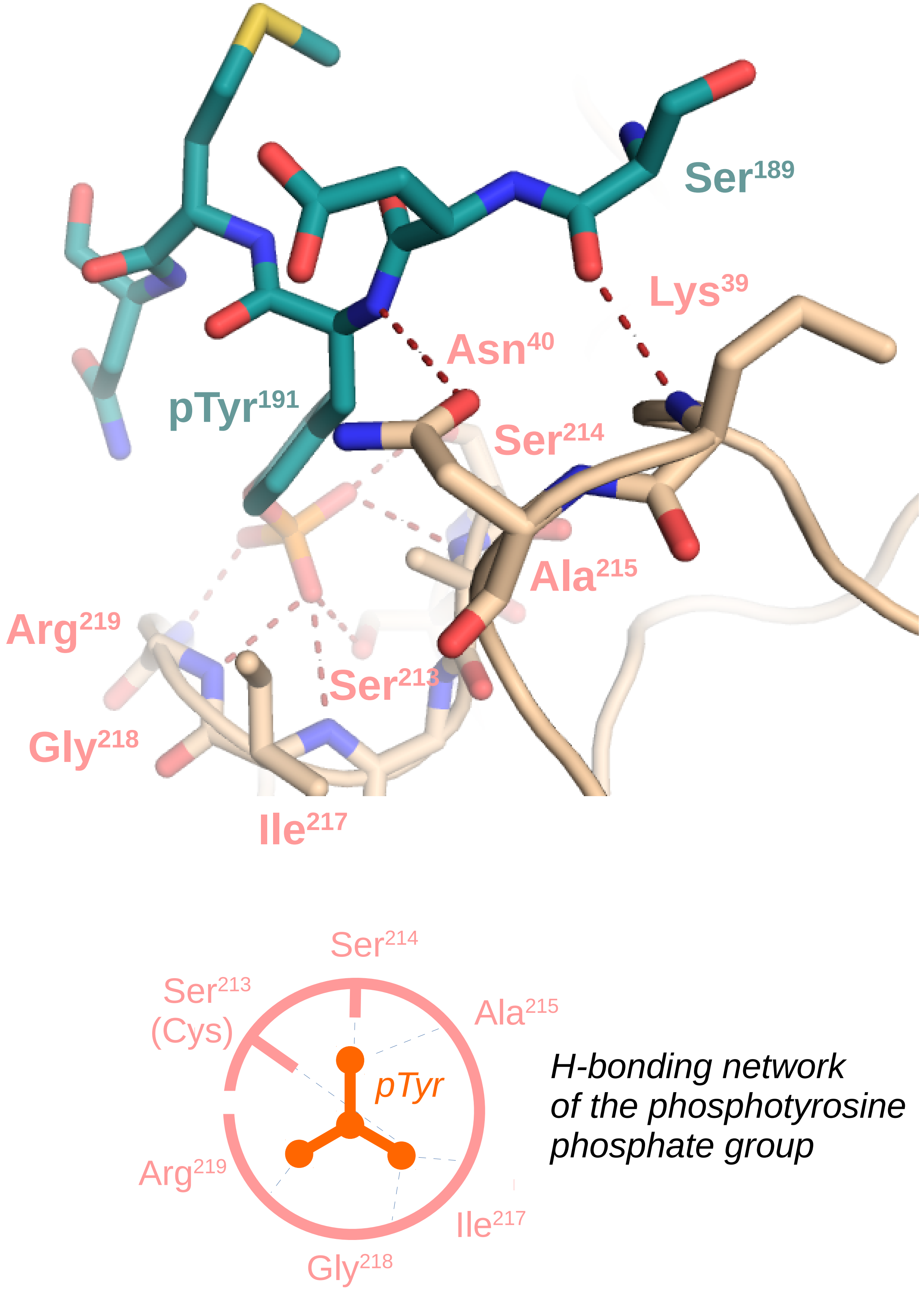

Supplementary figure 19. Inter-chain (red) polar contacts observed in the SHP2-ppCD28 structure

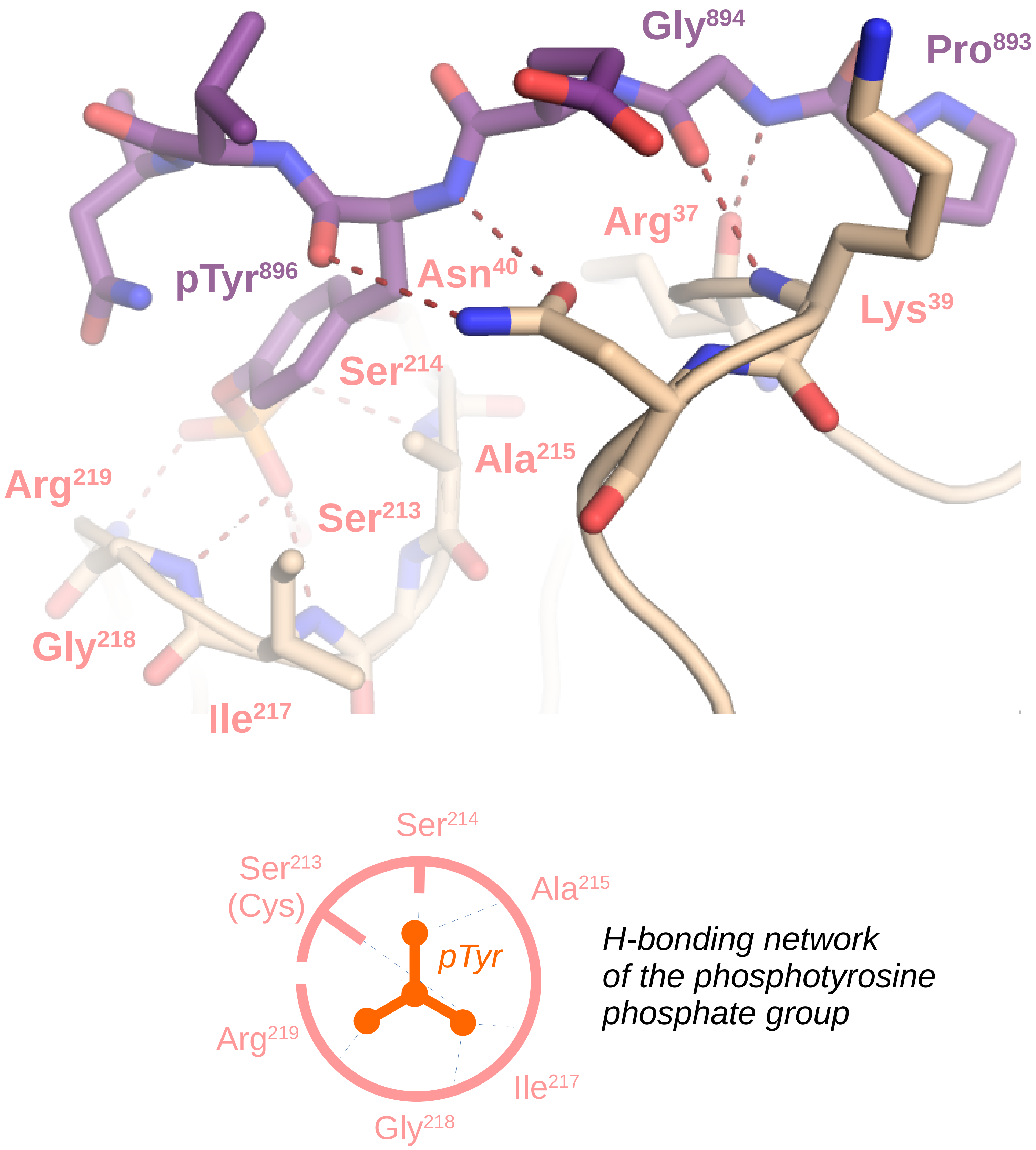

Supplementary figure 20. Inter-chain (red) polar contacts observed in the SHP2-ppSRev-IRS1 structure

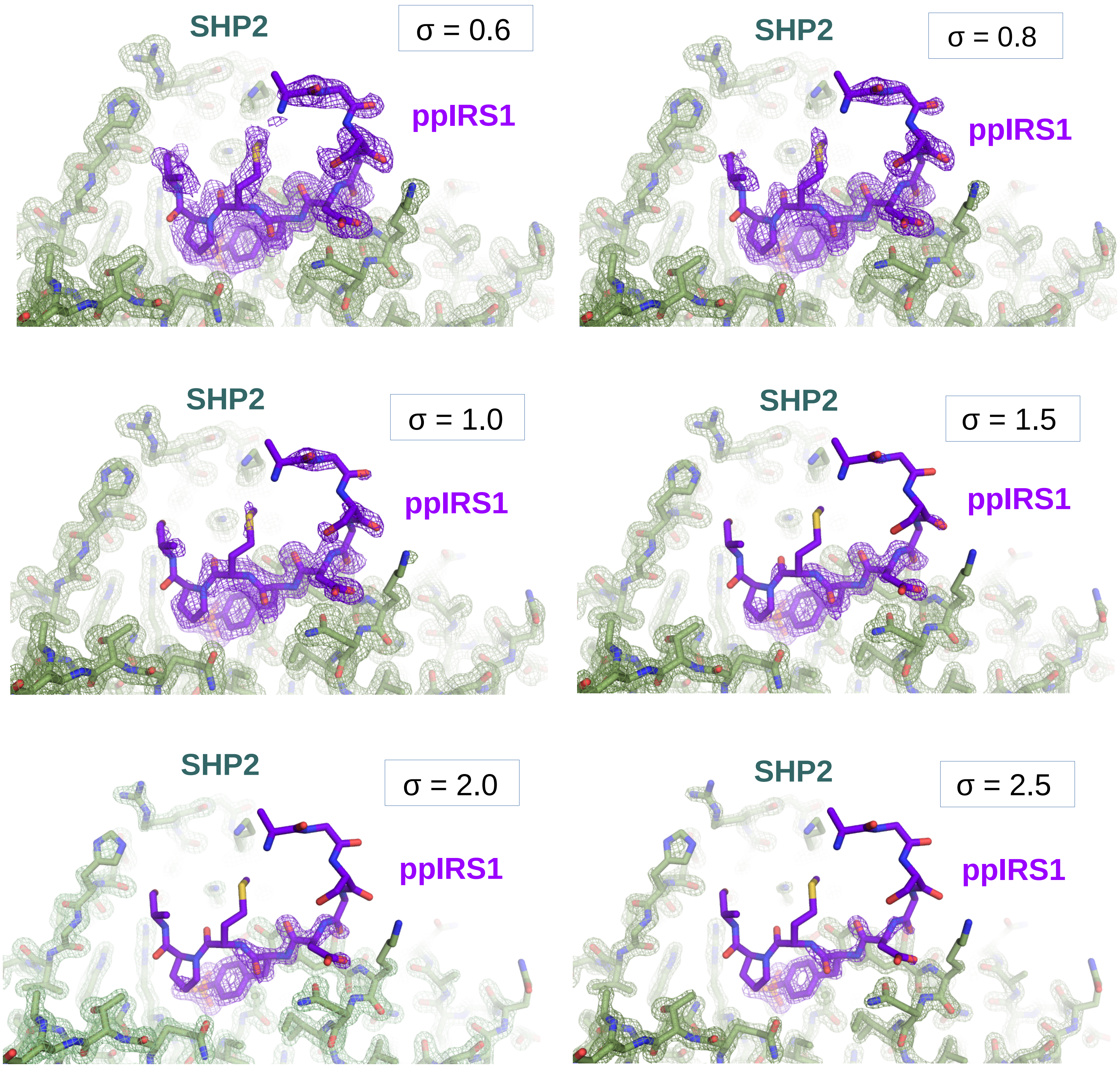

Supplementary figure 21. 2Fo-Fc maps of the ppIRS1-SHP2 complex

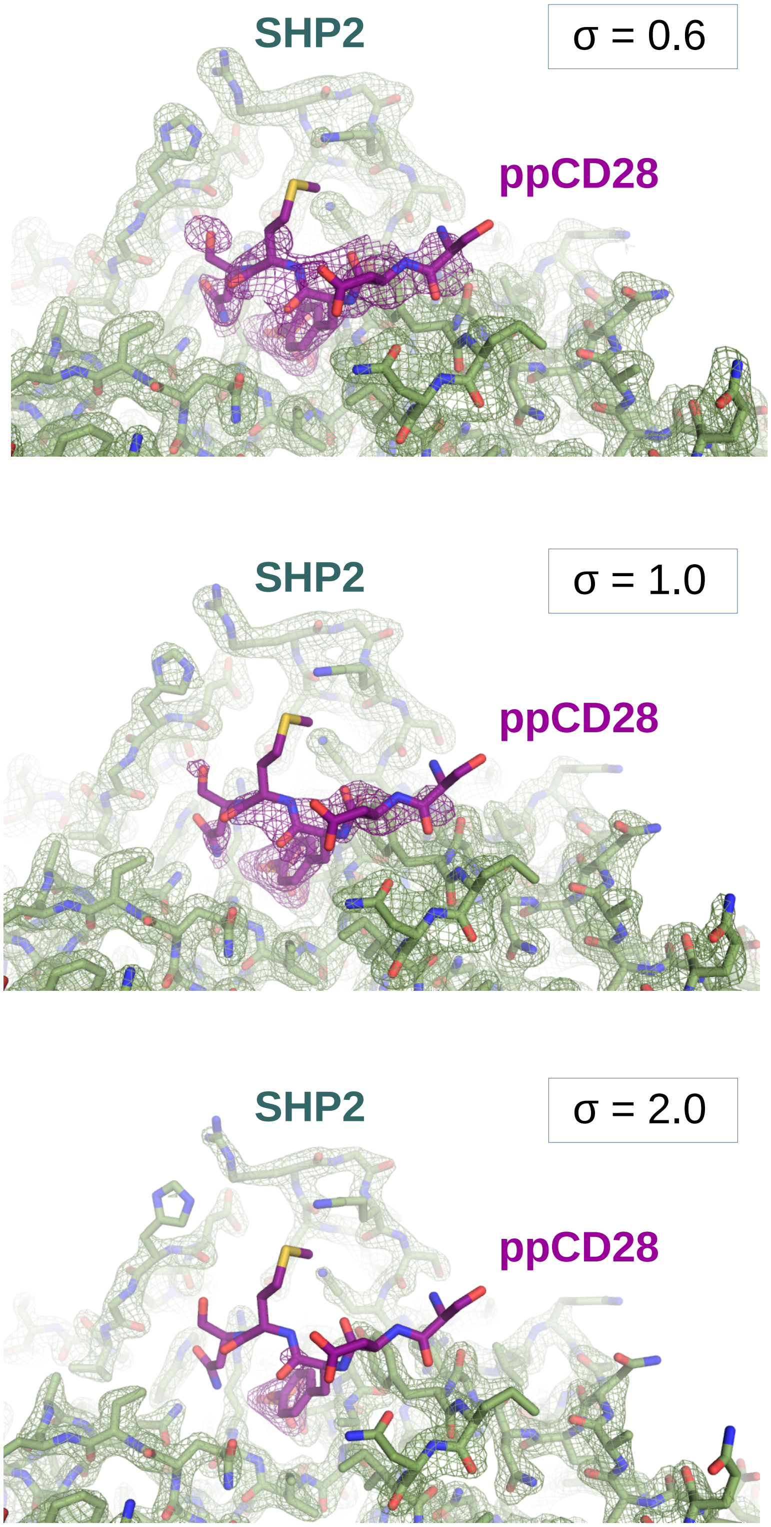

Supplementary figure 22. 2Fo-Fc maps of the ppCD28-SHP2 complex

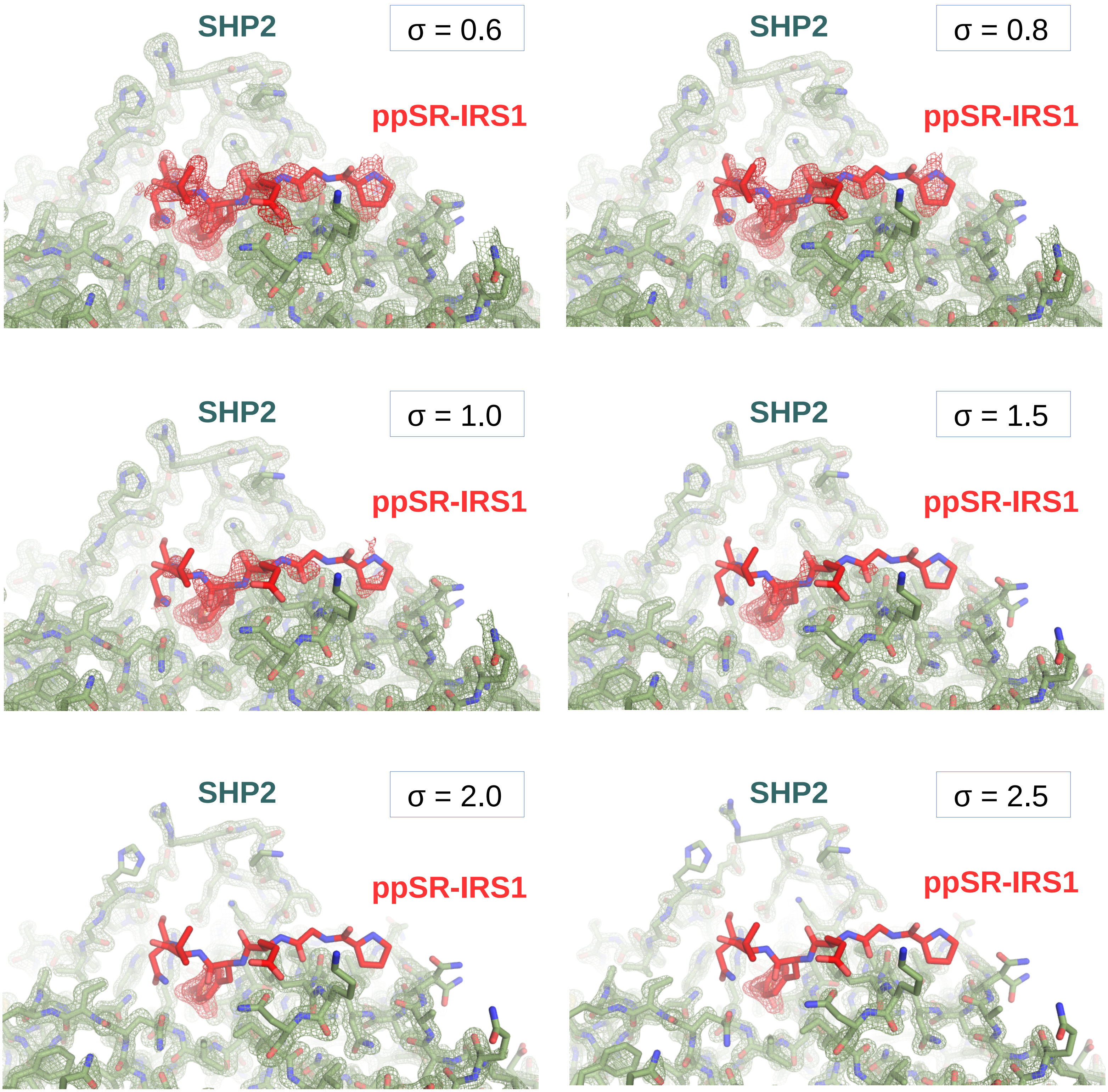

Supplementary figure 23. 2Fo-Fc maps of the ppSR-IRS1-SHP2 complex

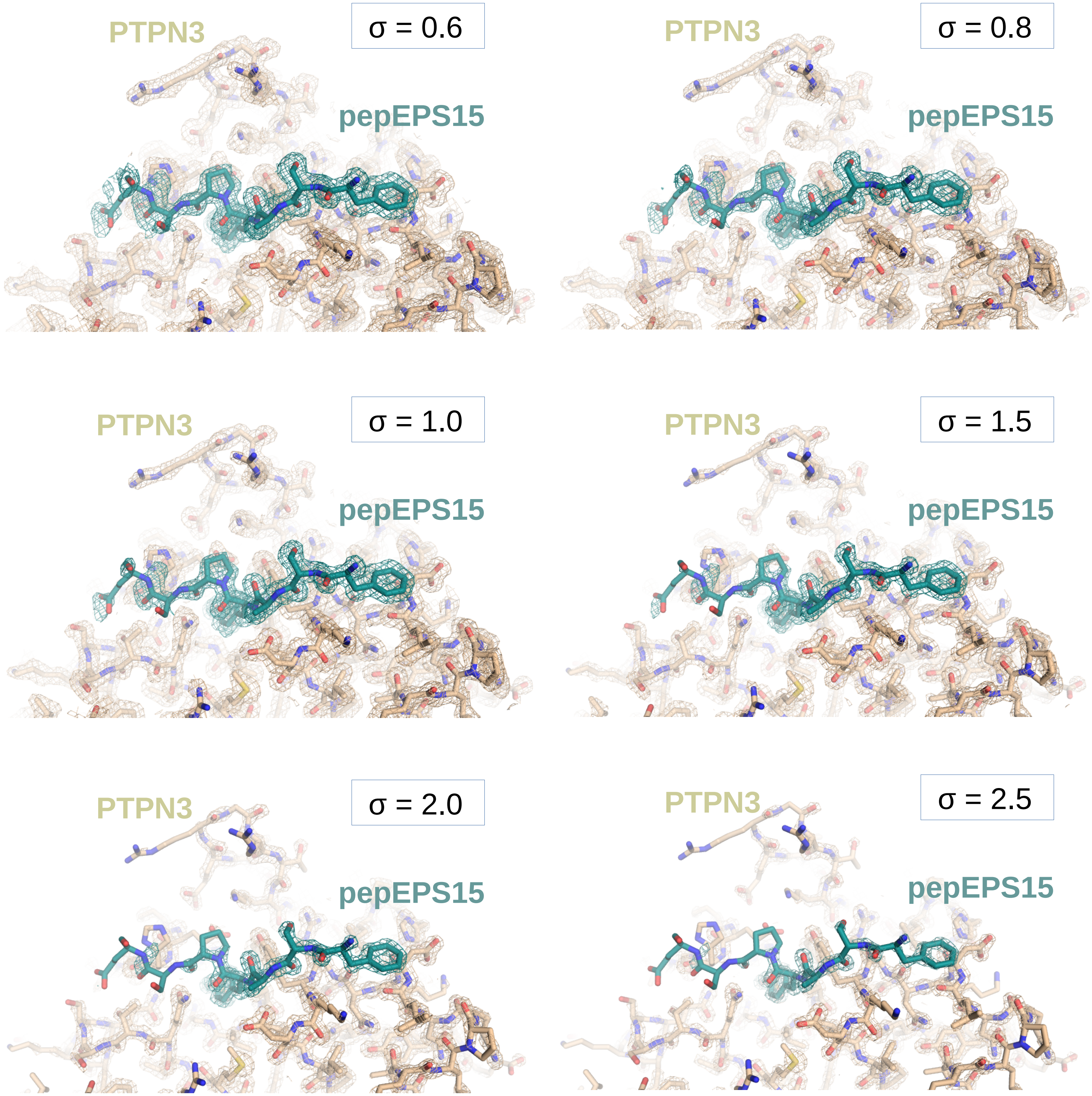

Supplementary figure 24. Comparative electron density maps of the published EPS15-PTPN3 complex PDB: 4RHG [1.58Å]

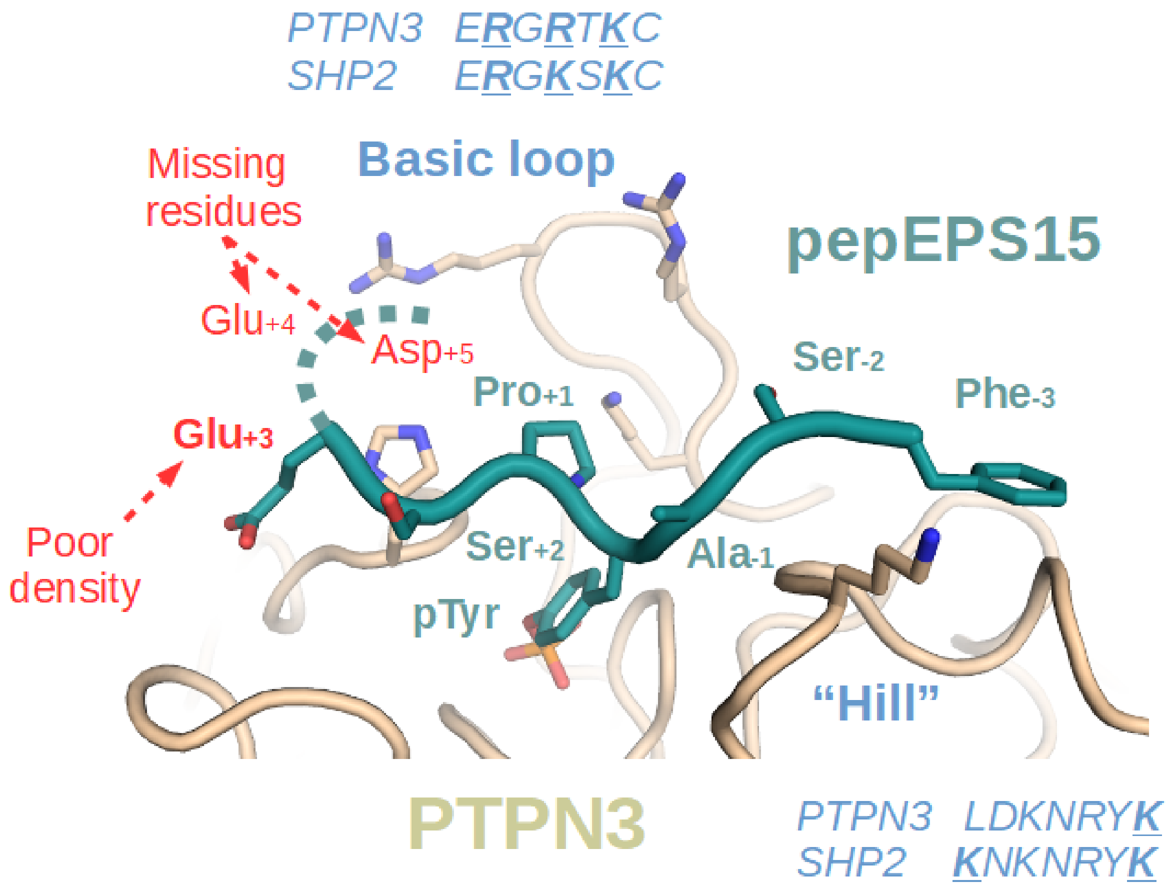

Alignment of vertebrate EPS15 proteins

```
P42566 - Human        NFANFSAYPSEEDMI
P42567 - Mouse        NFANFSAYPSEEDMI
F1NLF0 - Chicken      NFANFSTYSTEEDMI
G1KGR7 - Lizard       NFANFSAYPTEEDMI
A0A1L8GMJ7-Frog       GLNNFSTYQSEDYMI
F1QV21-Zebrafish      RFASFDKYPTEEDMI
A0A4W3IZD1-Shark      NFANFNTYPSEEDQI
```

Supplementary figure 25. Invisible substrate C-terminus in the PTPN3 - EPS15 complex (pdb: 4RHG) analogous to SHP2

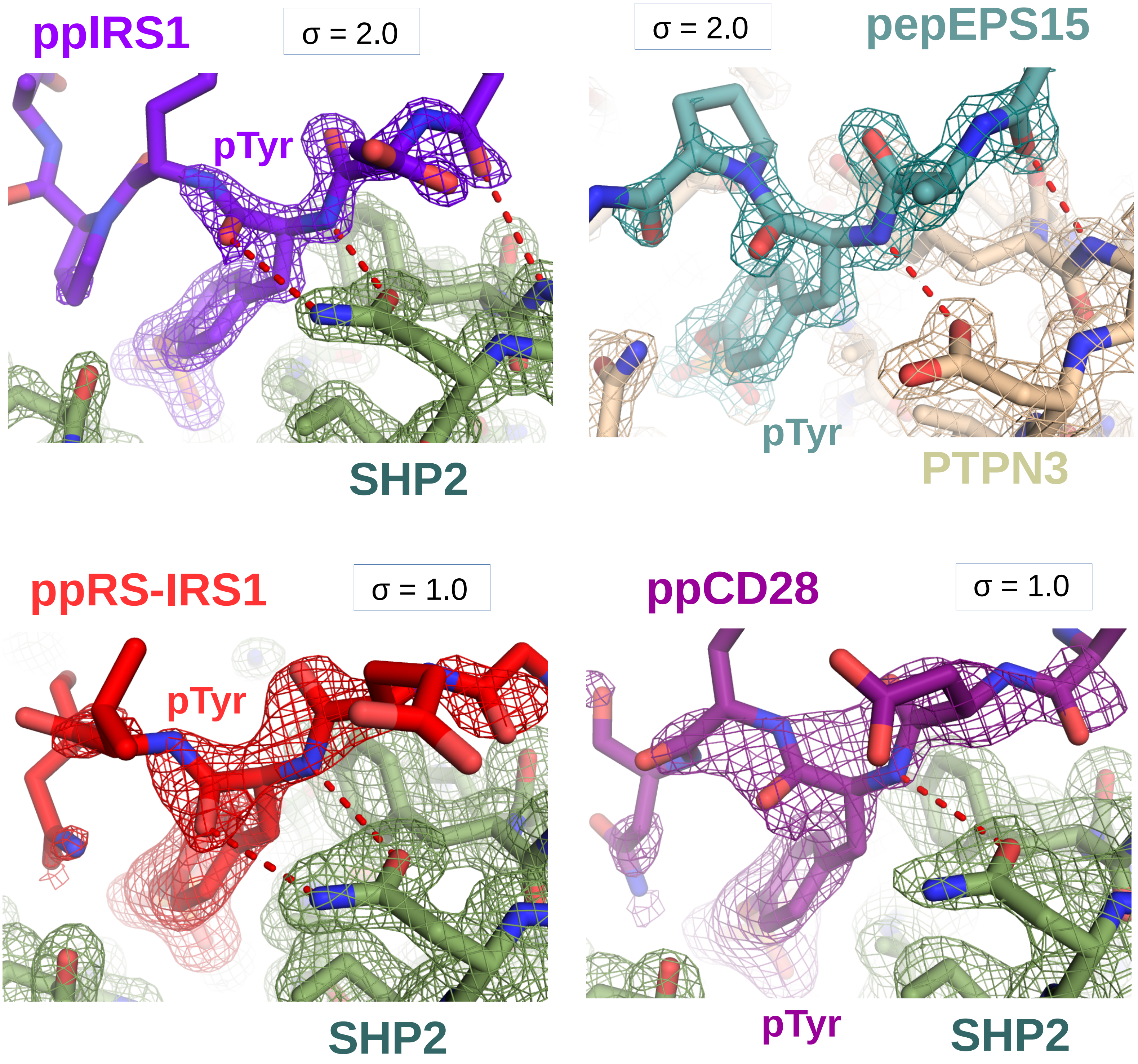

Supplementary figure 26. Density maps surrounding the pTyr residues with H-bonds suggested by structure

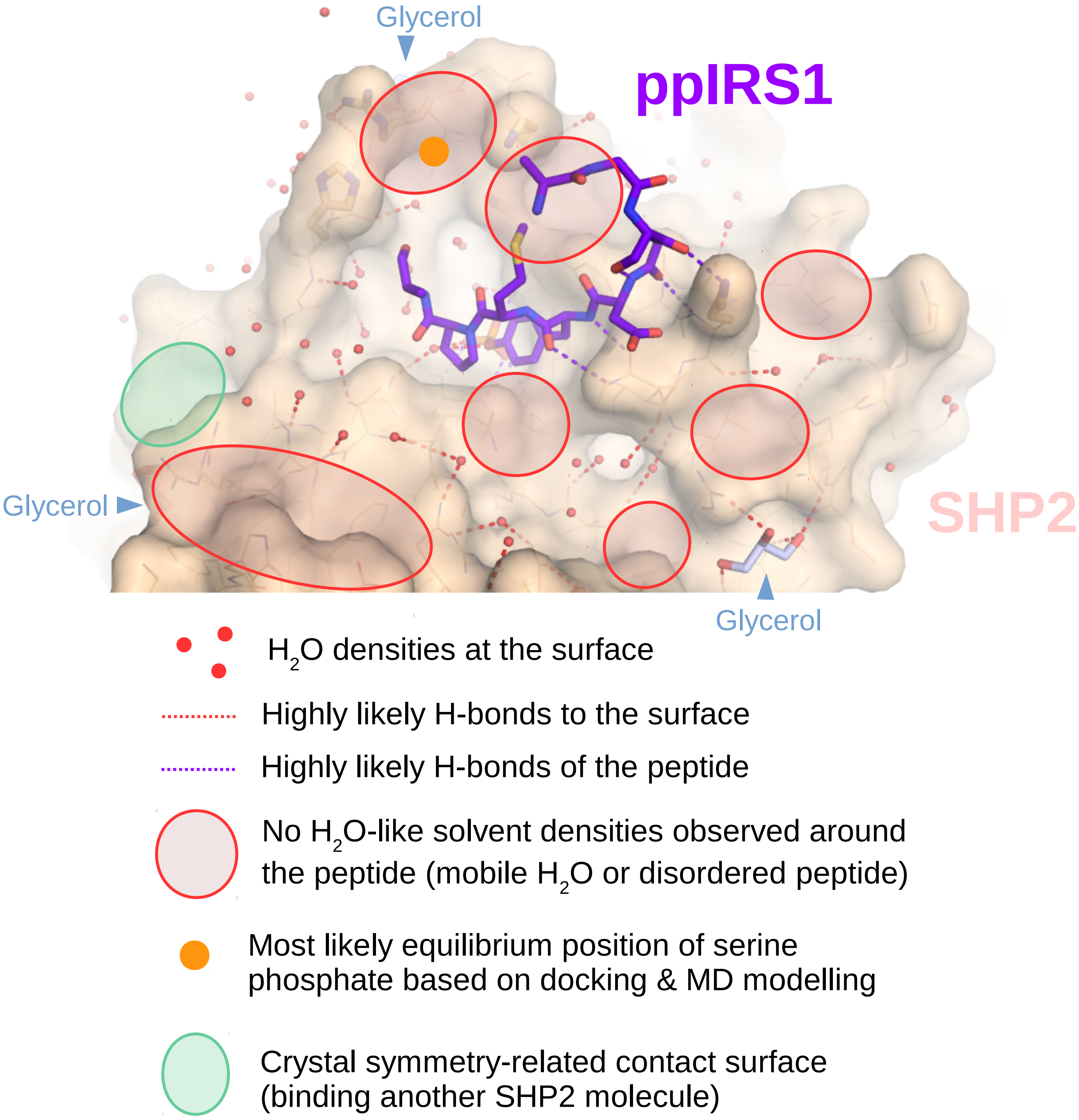

Supplementary figure 27. Surface areas devoid of densities clearly interpretable as bound H_2O molecules in the SHP2-ppIRS1 crystal (after taking symmetry-related molecules into consideration)

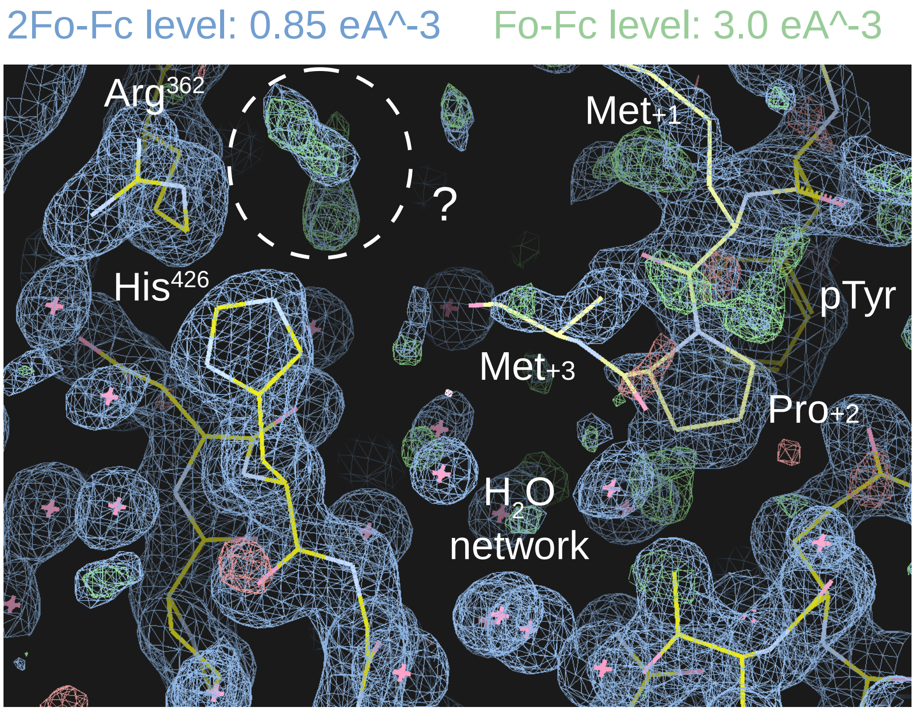

Supplementary figure 28. Lack of a well-defined density for the +4 PO_4 group in the SHP2-ppIRS1 crystal

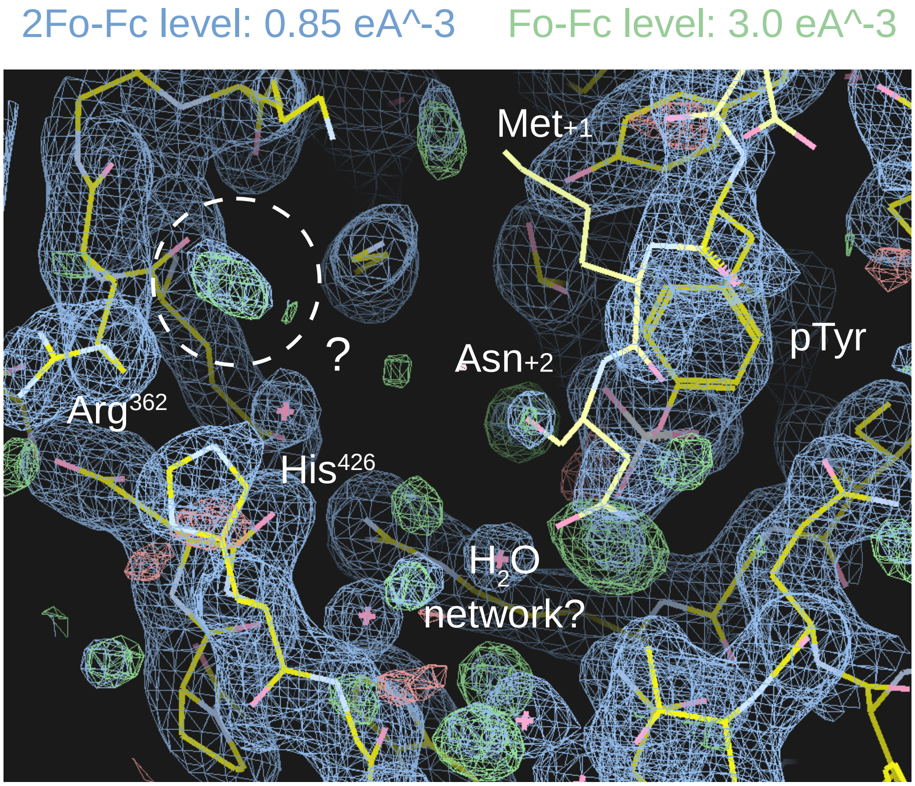

Supplementary figure 29. Lack of a well-defined density for the +4 PO_4 group in the SHP2-ppCD28 crystal

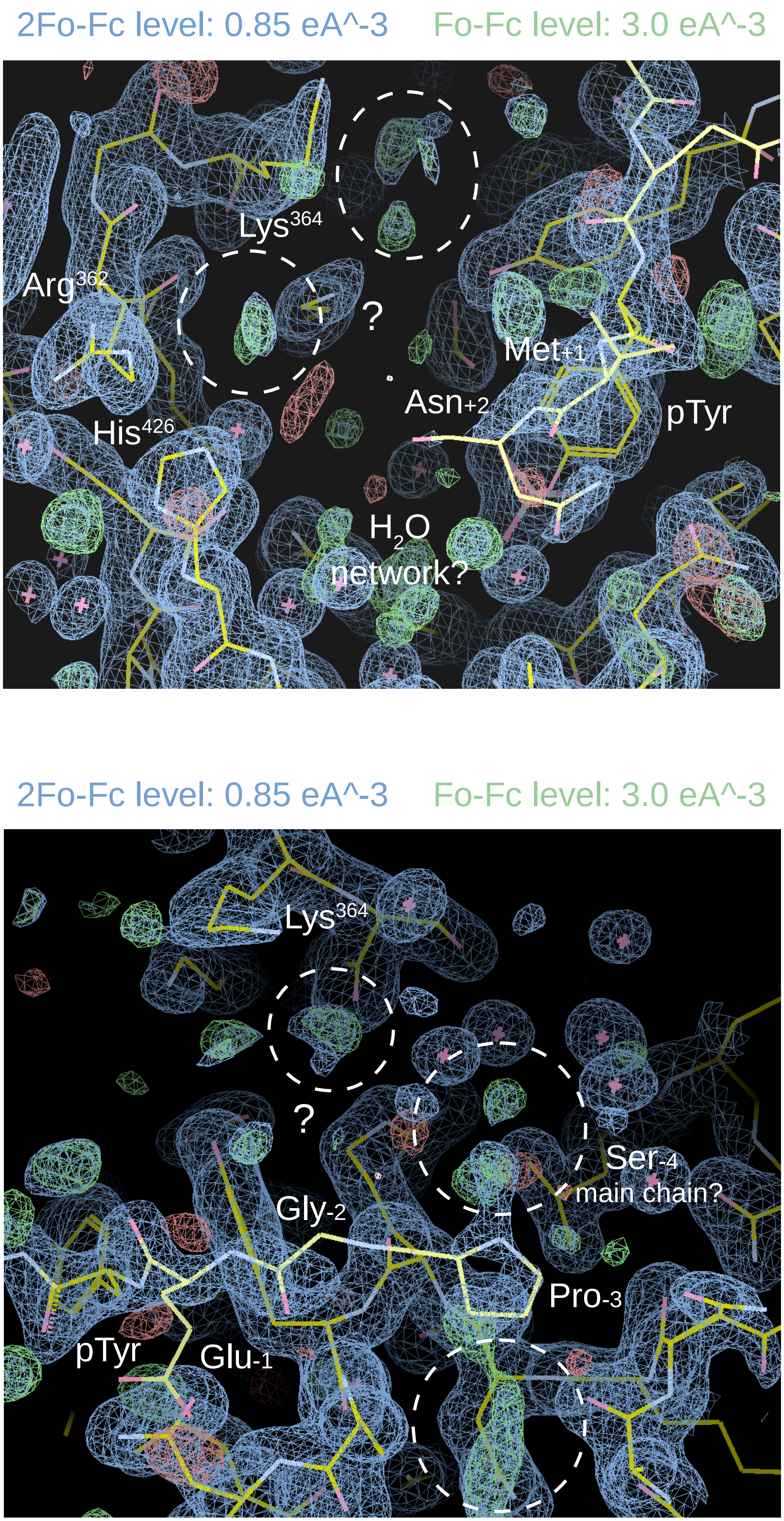

Supplementary figure 30. Lack of a well-defined density for the -4 PO_4 group or +4 Glu in the SHP2-ppSR-IRS1 crystal

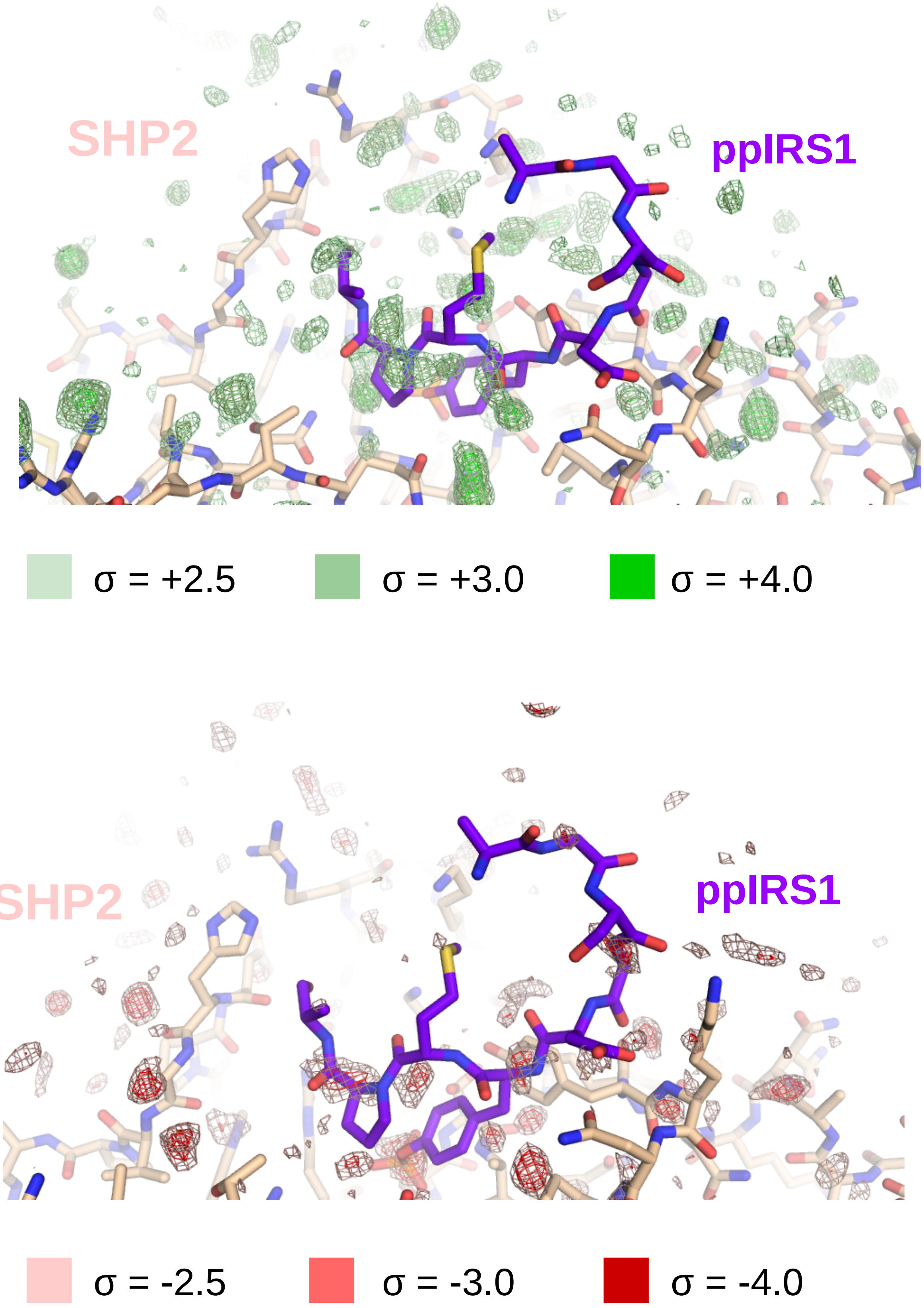

Supplementary figure 31. Positive and negative electron density difference maps (Fo-Fc) in the SHP2-ppIRS1 model

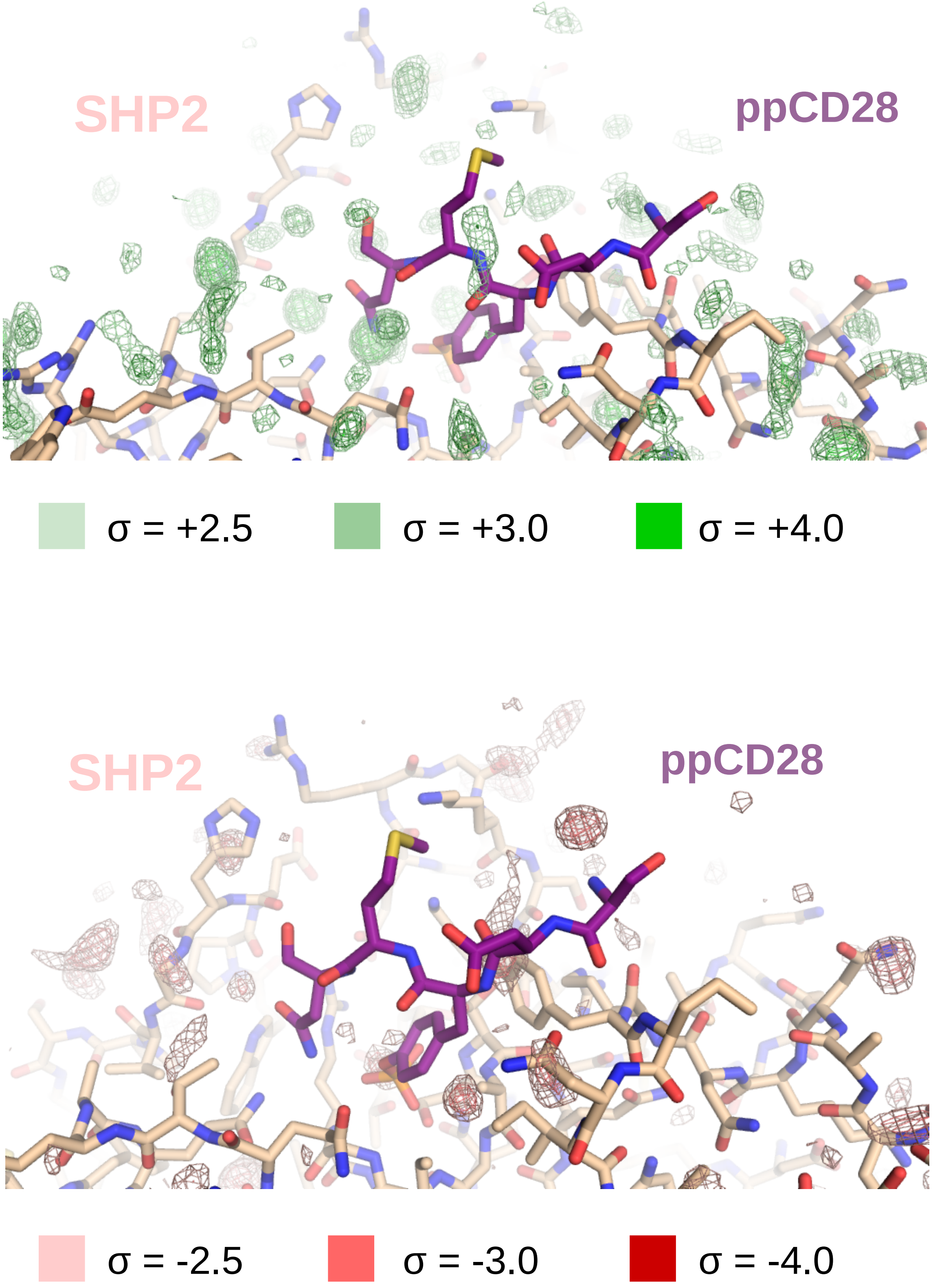

Supplementary figure 32. Positive and negative electron density difference maps (Fo-Fc) in the SHP2-ppCD28 model

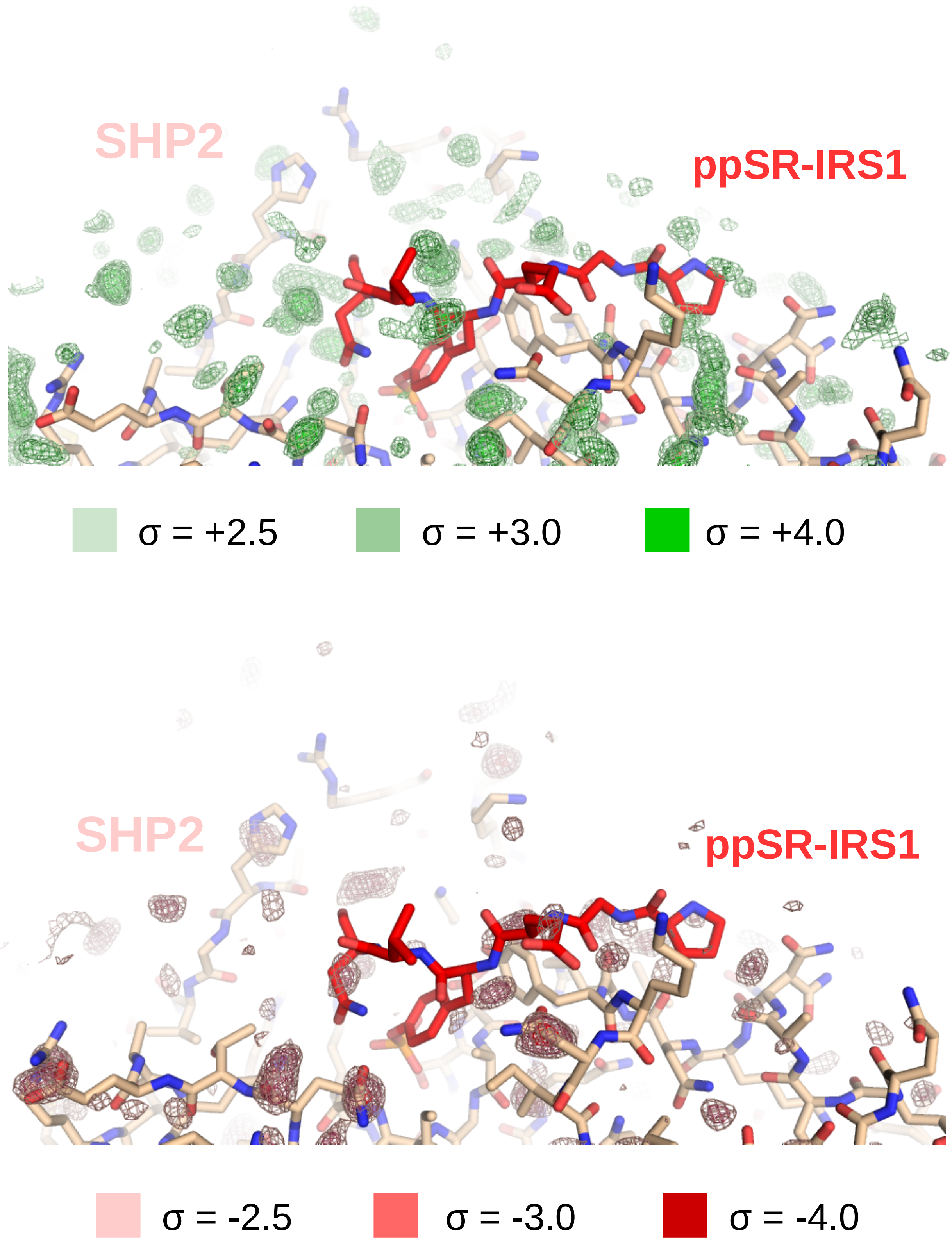

Supplementary figure 33. Positive and negative electron density difference maps (Fo-Fc) in the SHP2-ppSR-IRS1 model

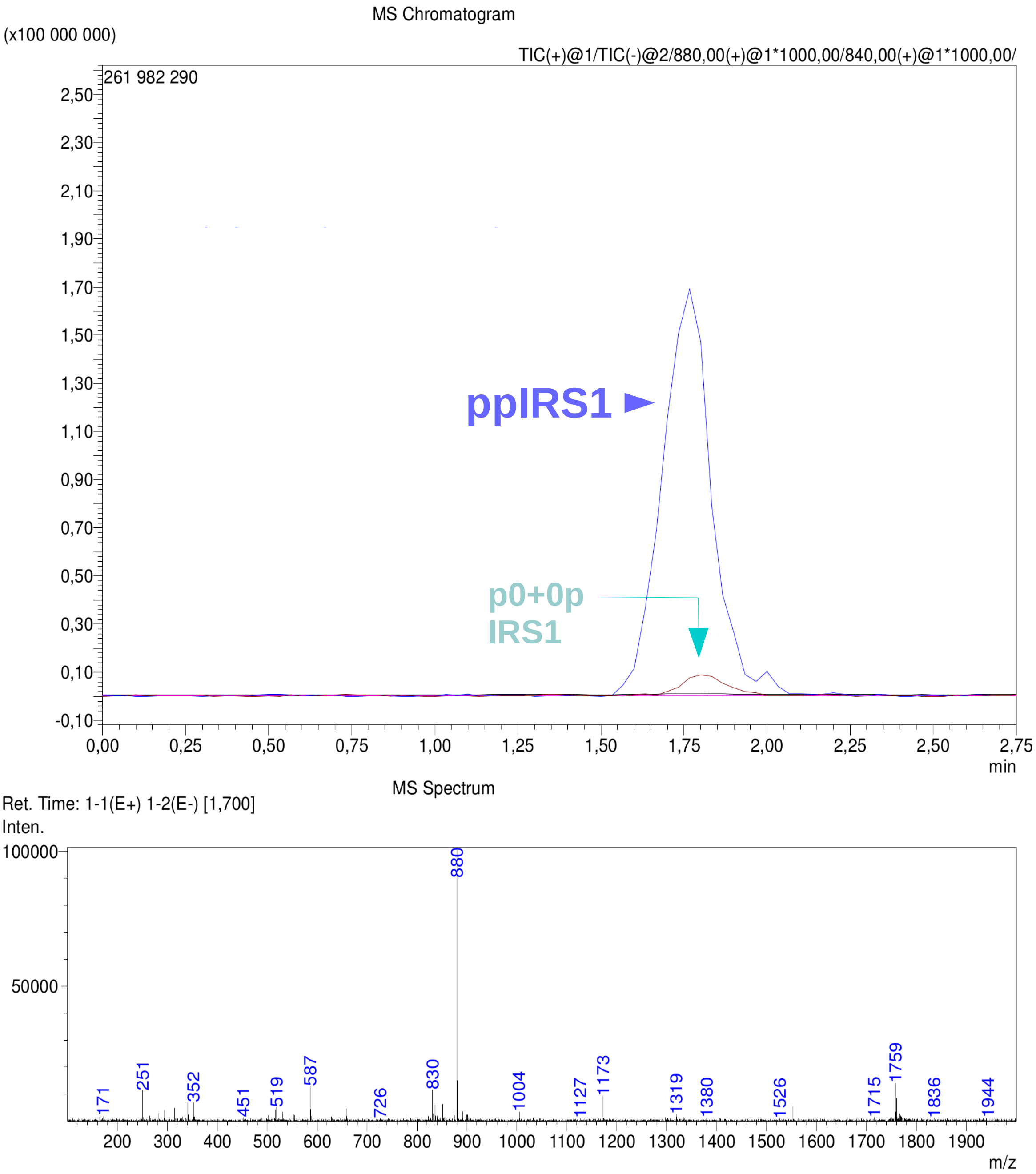

Supplementary figure 34. Intact MS analysis of ppIRS1 peptide: Mass spectrometry (MS) of ppIRS1 phosphopeptide used for crystallization, showing the chromatograms with a dominant doubly-phosphorylated form (blue), and a minute singly-phosphorylated contamination (cyan). Lower inset shows raw MS spectra dominated by $[M+H]^+$ (m/z predicted: 1758.7 found: 1759) and $[M+2H]^{2+}$ (m/z predicted: 879.8, found: 880) peaks of doubly phosphorylated peptide.

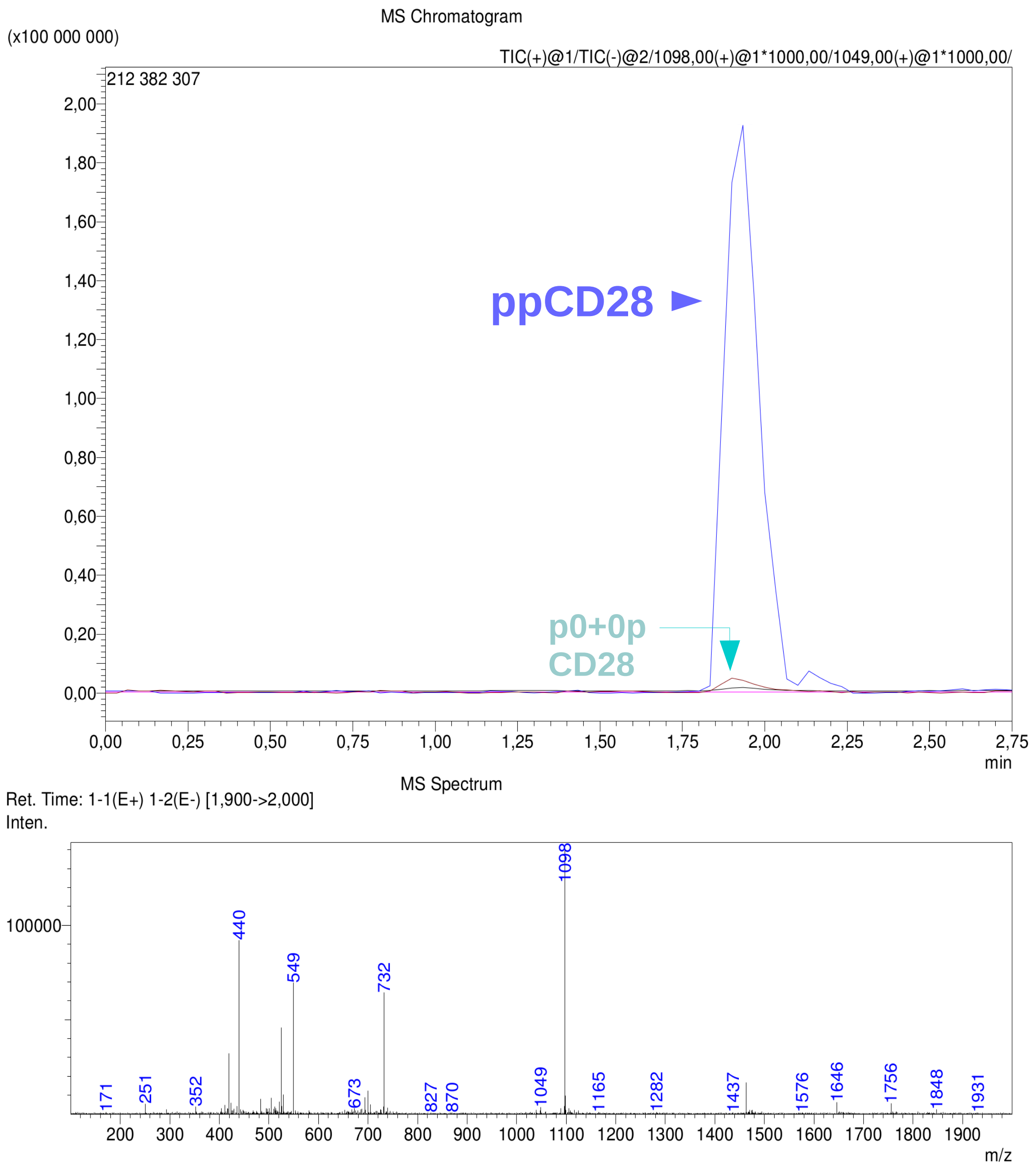

Supplementary figure 35. Intact MS analysis of ppCD28 peptide: Mass spectrometry (MS) of ppCD28 phosphopeptide used for crystallization, showing the chromatograms with a dominant doubly-phosphorylated form (blue), and a minute singly-phosphorylated contamination (cyan). Lower inset shows raw MS spectra dominated by the $[M+2H]^{2+}$ (m/z predicted: 1097.5, found: 1098), $[M+3H]^{3+}$ (m/z predicted: 732.0, found: 732), $[M+4H]^{4+}$ (m/z predicted: 549.2, found: 549) and $[M+5H]^{5+}$ (m/z predicted: 439.6, found: 440) peaks of doubly phosphorylated peptide.

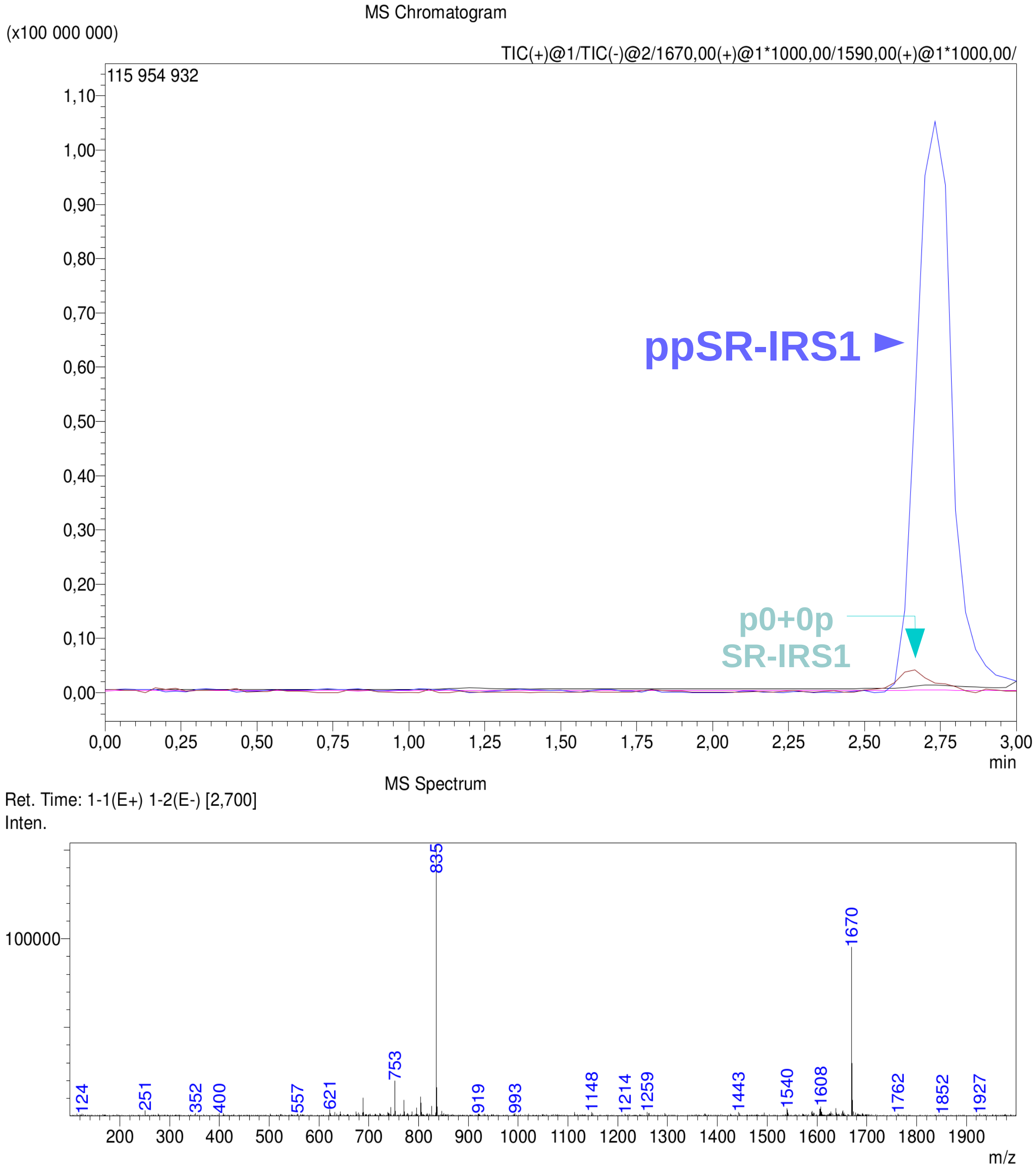

Supplementary figure 36. Intact MS analysis of ppSR-IRS1 peptide: Mass spectrometry (MS) of ppSR-IRS1 phosphopeptide used for crystallization, showing the chromatograms with a dominant doubly-phosphorylated form (blue), and a minute singly-phosphorylated contamination (cyan). Lower inset shows raw MS spectra dominated by $[M+H]^+$ (m/z predicted: 1669.7, found: 1670) and $[M+2H]^{2+}$ (m/z predicted: 835.3, found: 835) peaks of doubly phosphorylated peptide.

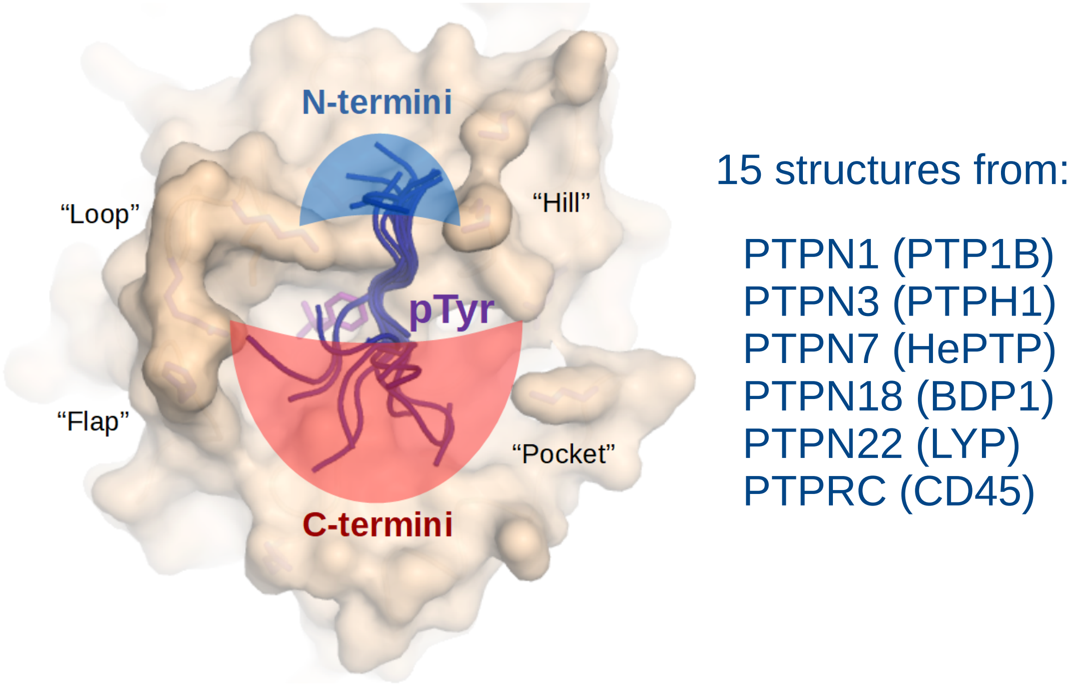

Supplementary figure 37. Phosphatase substrate N- and C-terminal segments fanning out at localized regions (blue and red) in known structures of enzyme-substrate complexes superimposed over SHP2

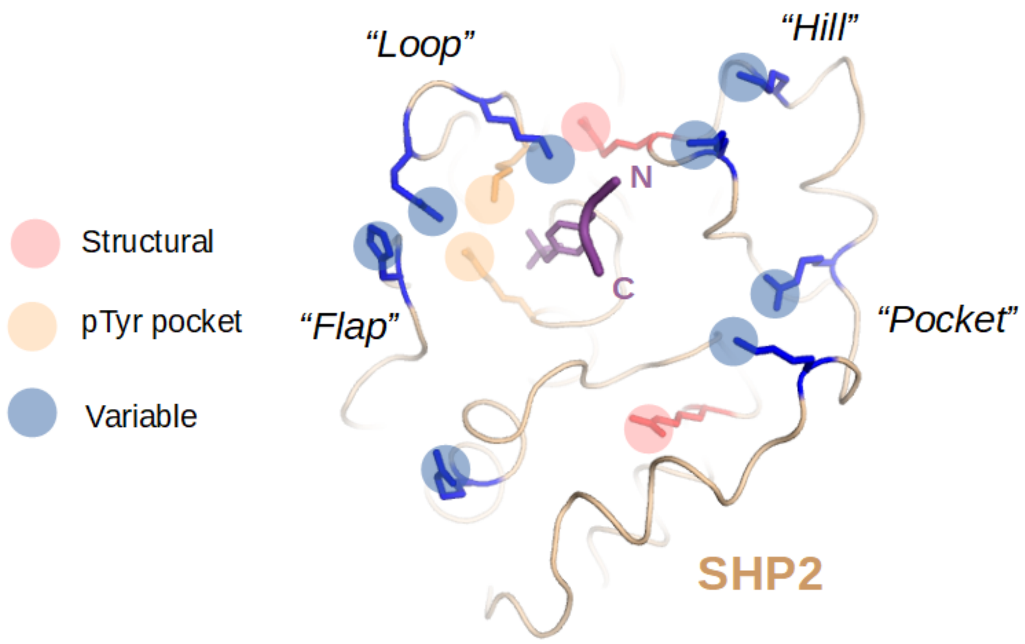

Supplementary figure 38. Surface charge distribution of SHP2 phosphatase with variable and invariant basic amino acids across PTPs

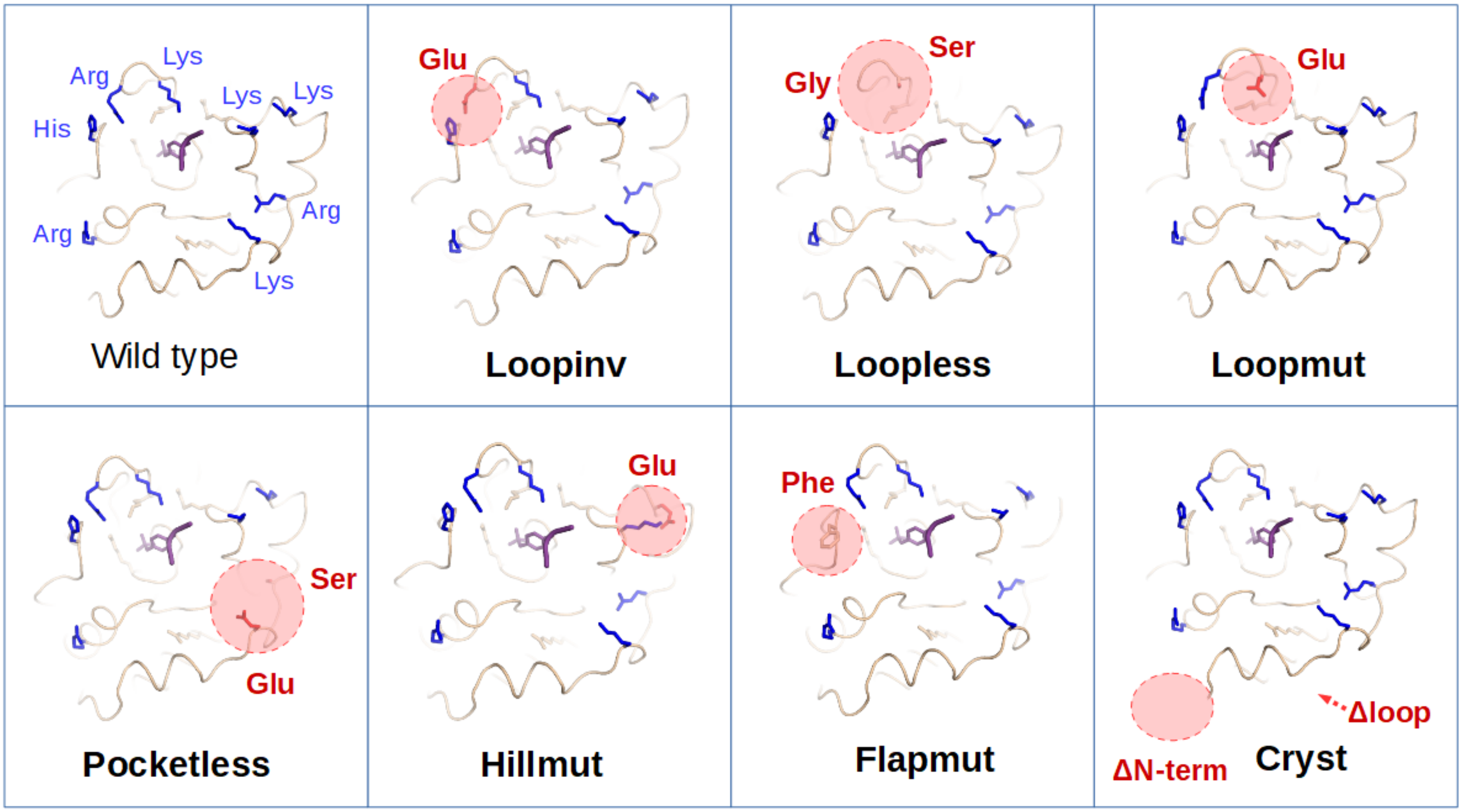

Supplementary figure 39. All SHP2 surface mutants designed and used for validation of peptide coordination models.

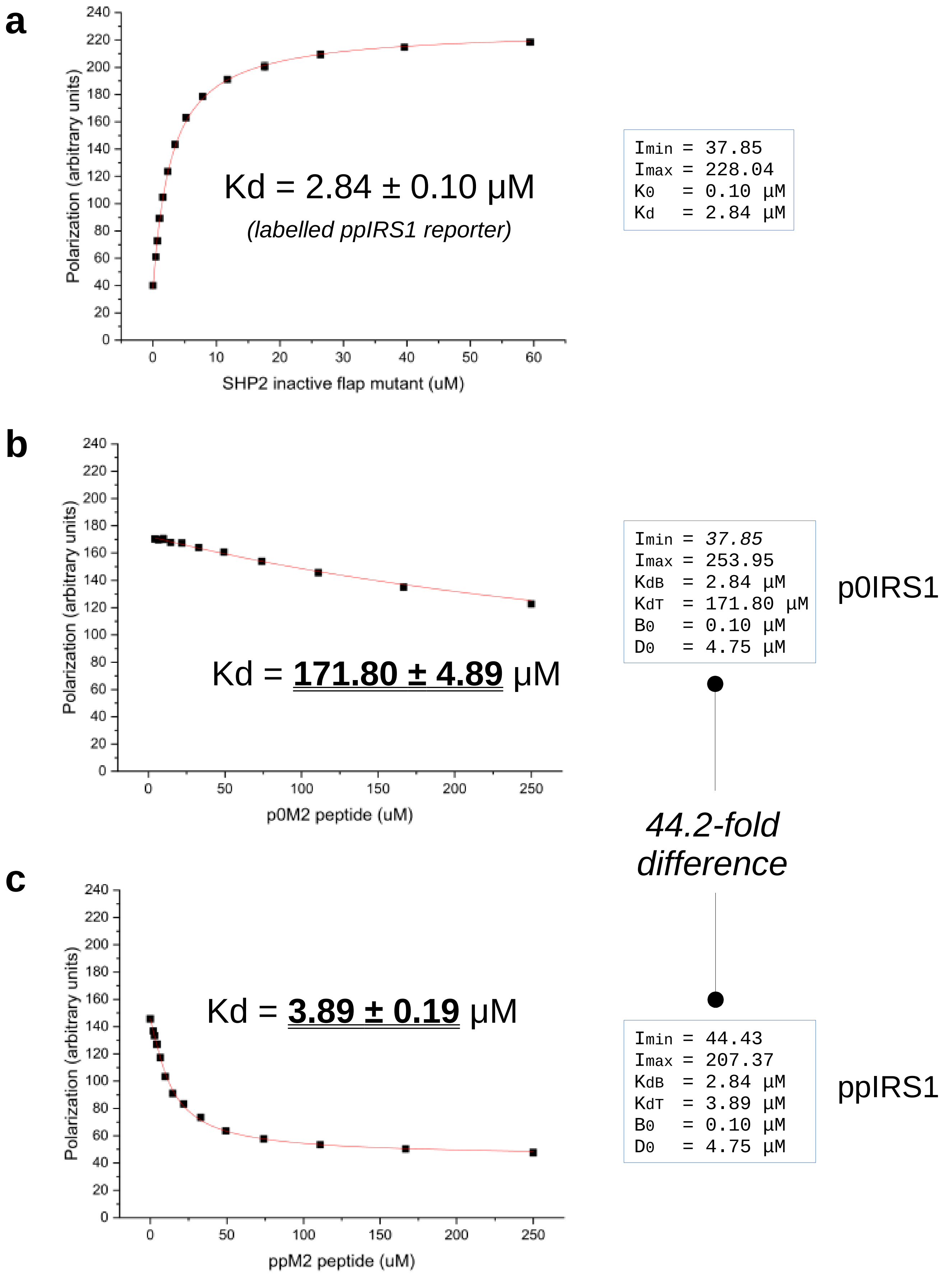

Supplementary figure 40. Flap mutant (H430F) inactive SHP2 direct (a) and competitive (b,c) fluorescence polarization titrations (p0M2 = p0IRS1 vs. ppM2 = ppIRS1) (n=3 technical replicates, error bars show ±SD for each point). Source data are provided as a Source Data file.

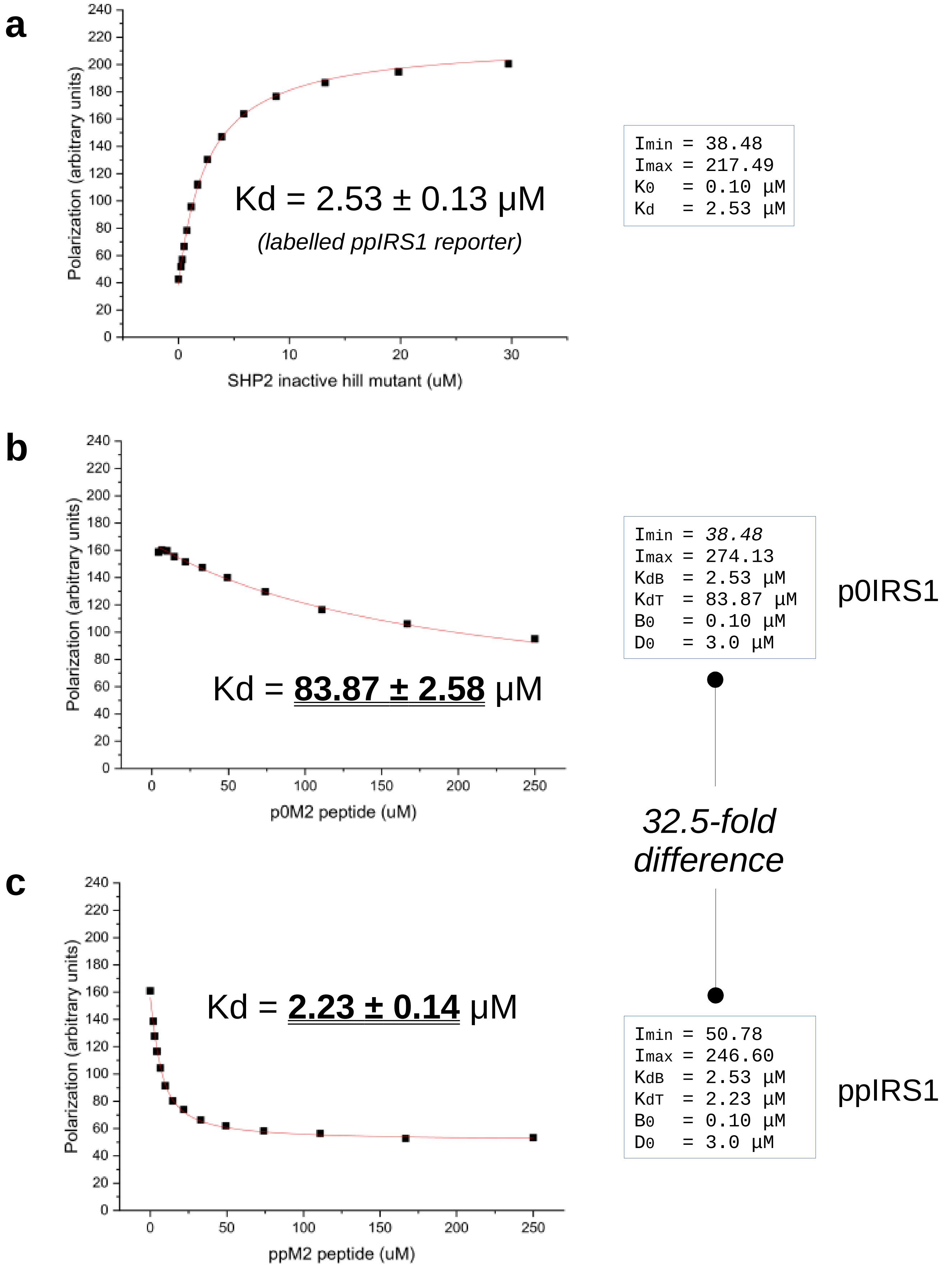

Supplementary figure 41. Hill mutant (K274E) inactive SHP2 direct (a) and competitive (b,c) fluorescence polarization titrations (p0M2 = p0IRS1 vs. ppM2 = ppIRS1) (n=3 technical replicates, error bars show ±SD for each point). Source data are provided as a Source Data file.

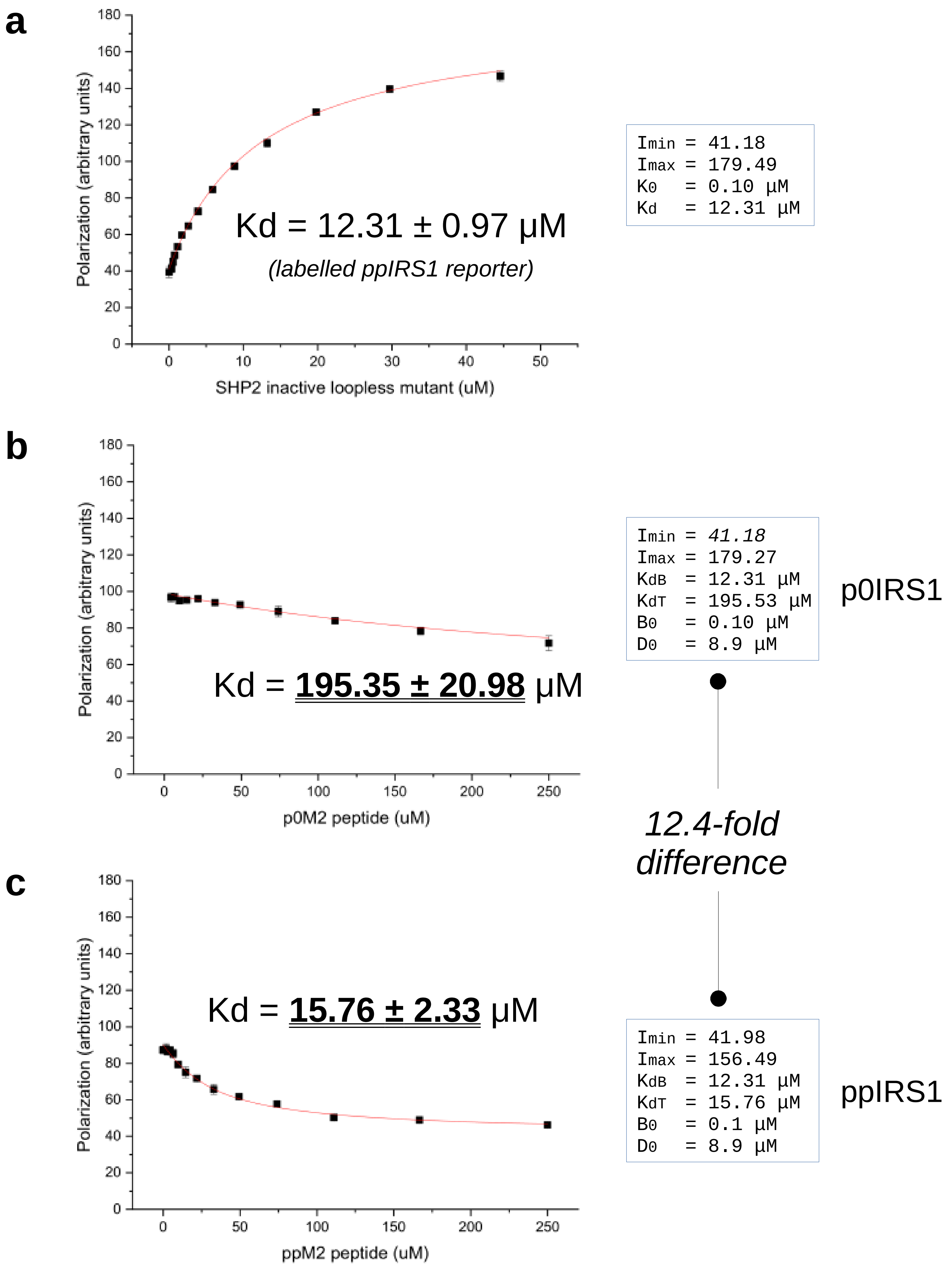

Supplementary figure 42. Loopless (R362G+K364S) inactive SHP2 direct (a) and competitive (b,c) fluorescence polarization titrations (p0M2 = p0IRS1 vs. ppM2 = ppIRS1) (n=3 technical replicates, error bars show ±SD for each point). Source data are provided as a Source Data file.

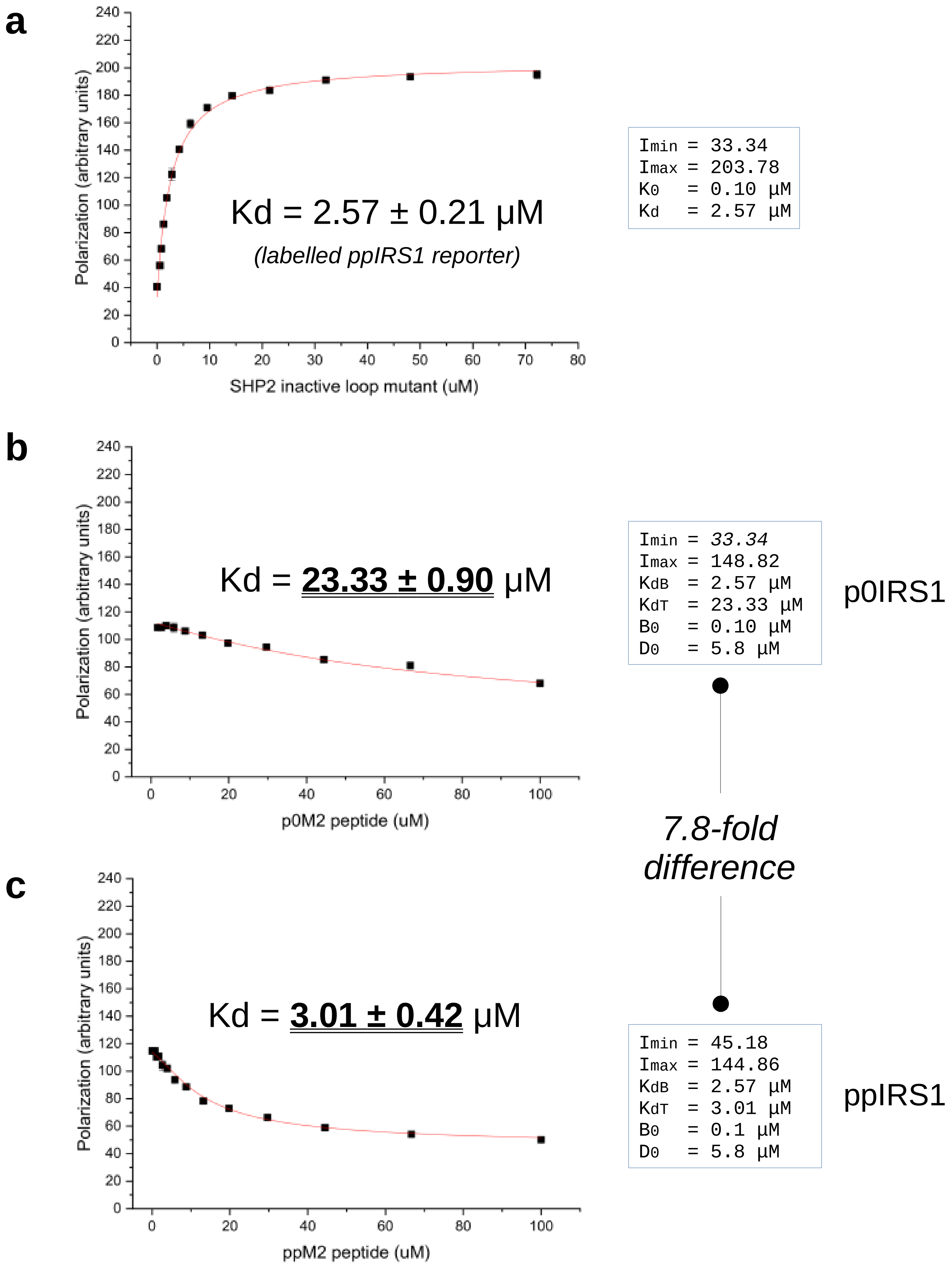

Supplementary figure 43. Loop mutant (K364E) inactive SHP2 direct (a) and competitive (b,c) fluorescence polarization titrations (p0M2 = p0IRS1 vs. ppM2 = ppIRS1) (n=3 technical replicates, error bars show ±SD for each point). Source data are provided as a Source Data file.

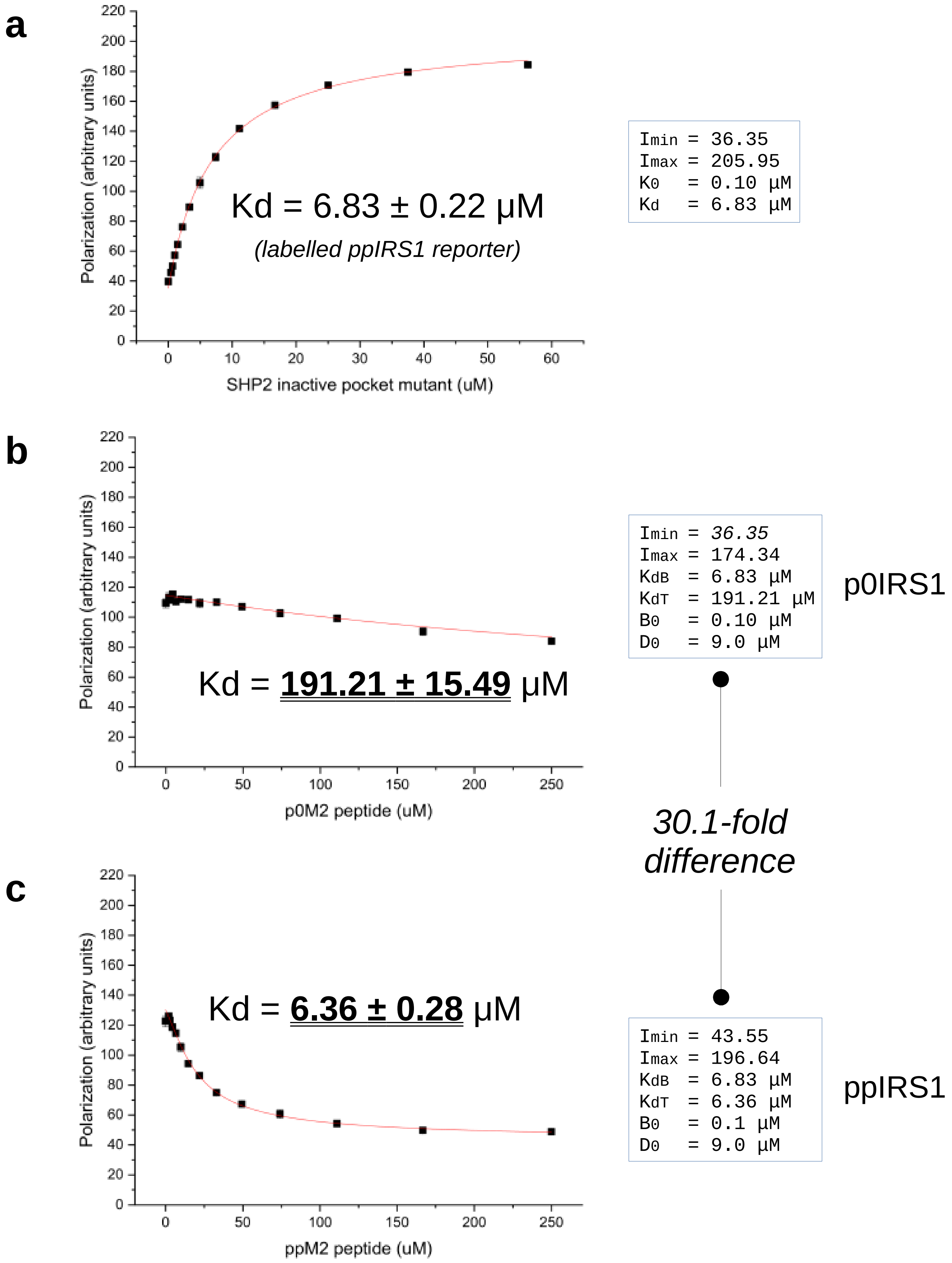

Supplementary figure 44. Pocketless (K260E+R265S) inactive SHP2 direct (a) and competitive (b,c) fluorescence polarization titrations (p0M2 = p0IRS1 vs. ppM2 = ppIRS1) (n=3 technical replicates, error bars show ±SD for each point). Source data are provided as a Source Data file.

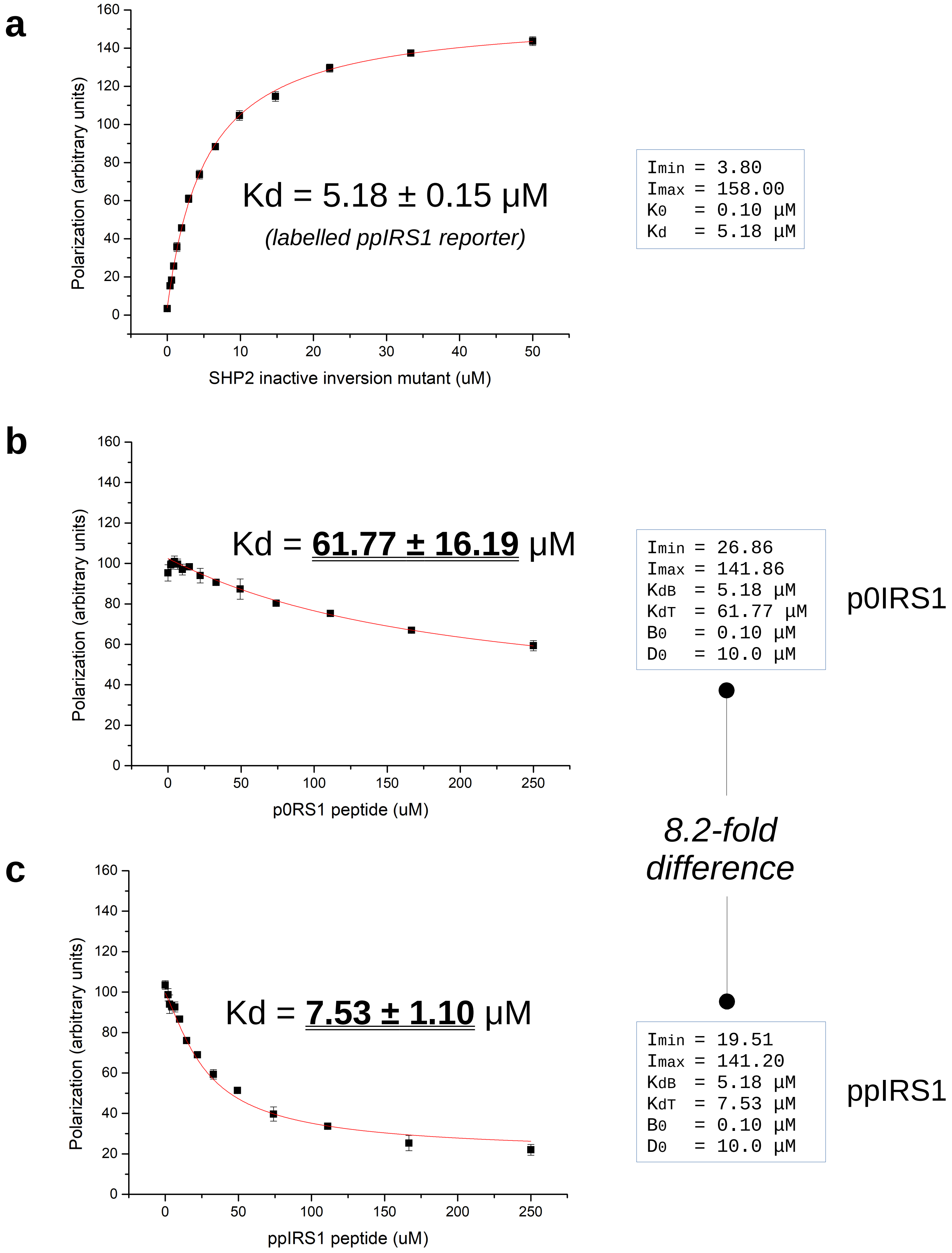

Supplementary figure 45. Loop inversion mutant (R362E) inactive SHP2 direct (a) and competitive (b,c) fluorescence polarization titrations (p0IRS1 vs. ppIRS1) (n=3 technical replicates, error bars show ±SD for each point).
Source data are provided as a Source Data file.

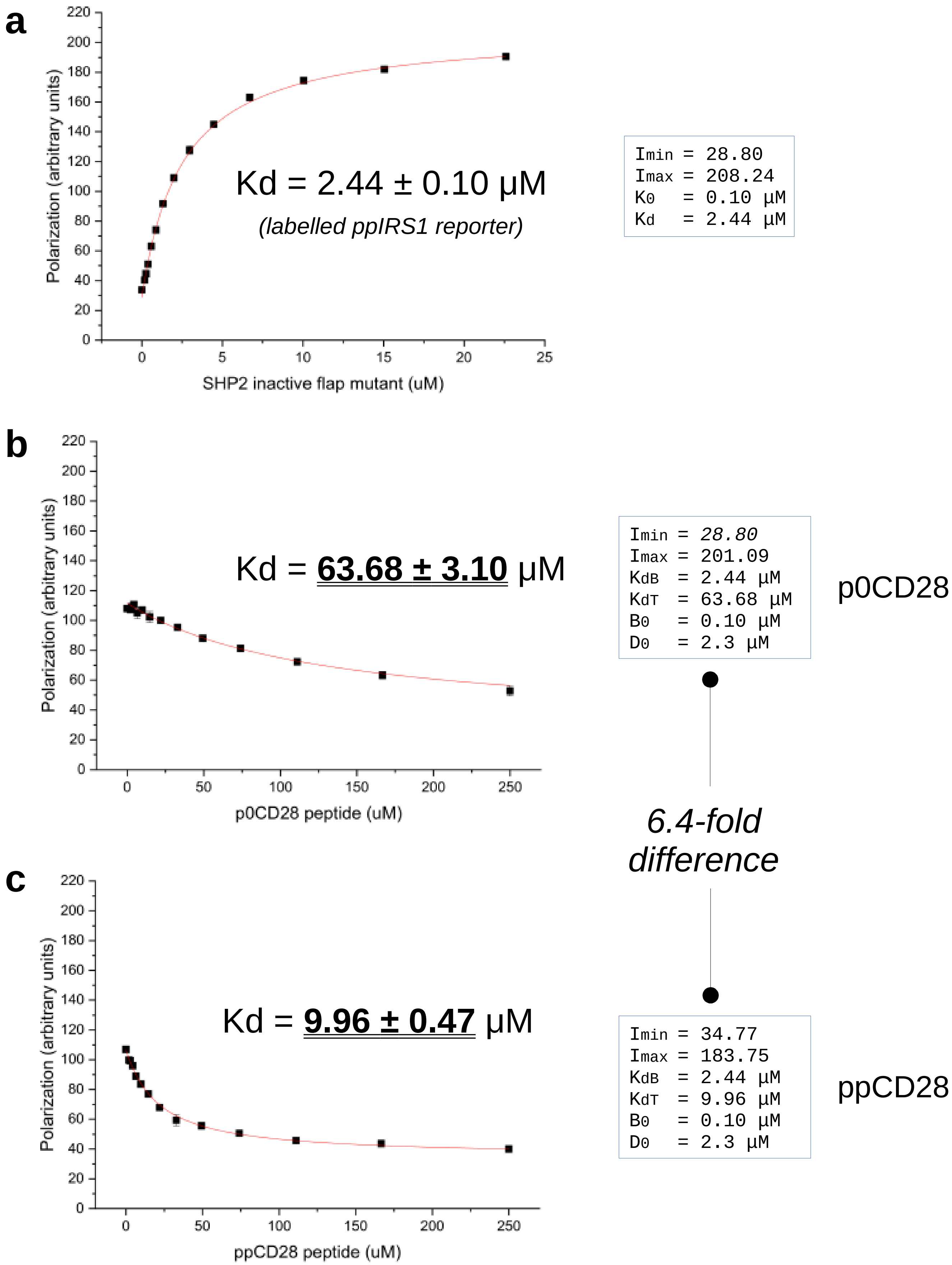

Supplementary figure 46. Flap mutant (H430F) inactive SHP2 direct (a) and competitive (b,c) fluorescence polarization titrations (p0CD28 vs. ppCD28) (n=3 technical replicates, error bars show ±SD for each point).
Source data are provided as a Source Data file.

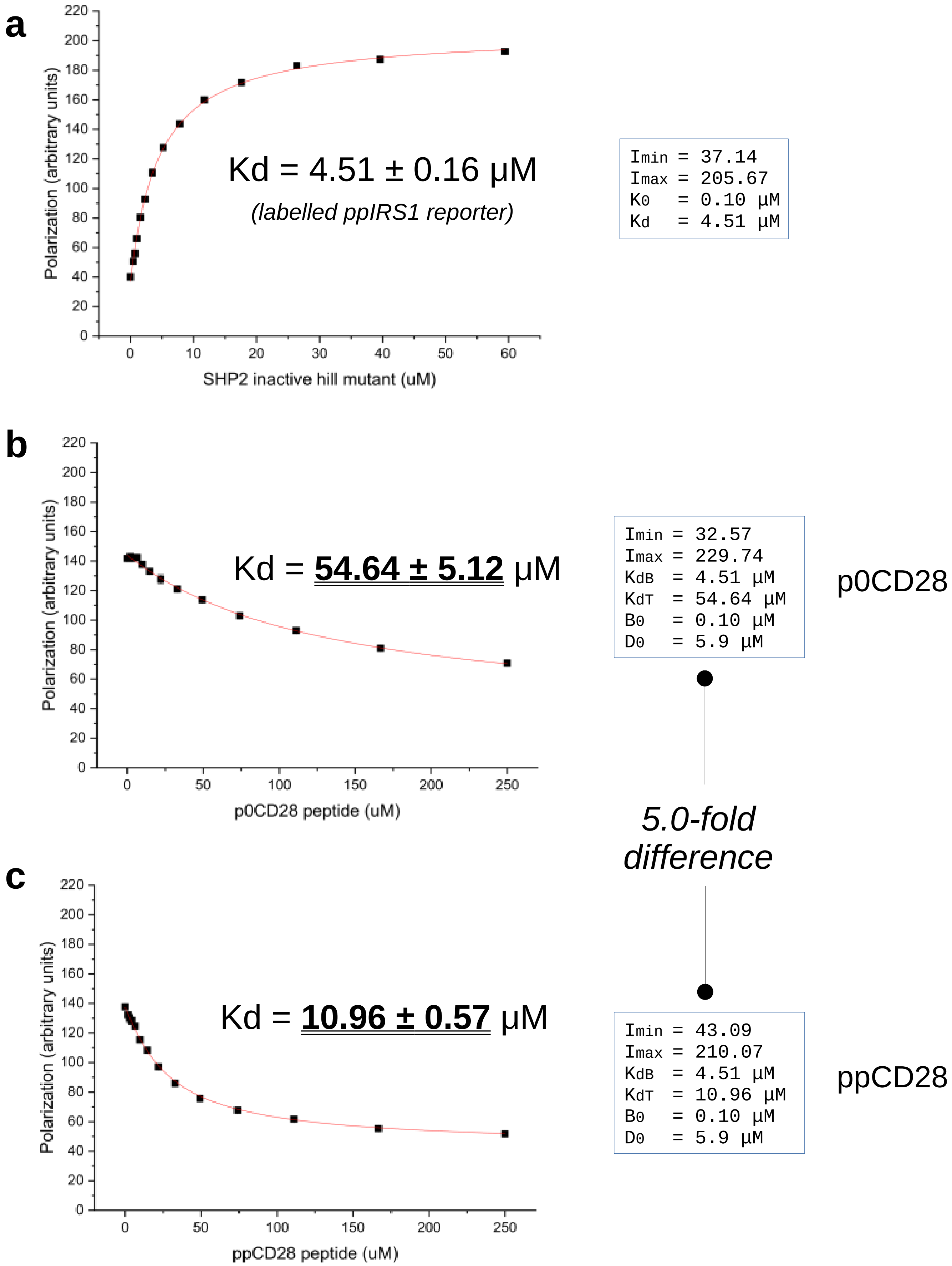

Supplementary figure 47. Hill mutant (K274E) inactive SHP2 direct (a) and competitive (b,c) fluorescence polarization titrations (p0CD28 vs. ppCD28) (n=3 technical replicates, error bars show ±SD for each point).
Source data are provided as a Source Data file.

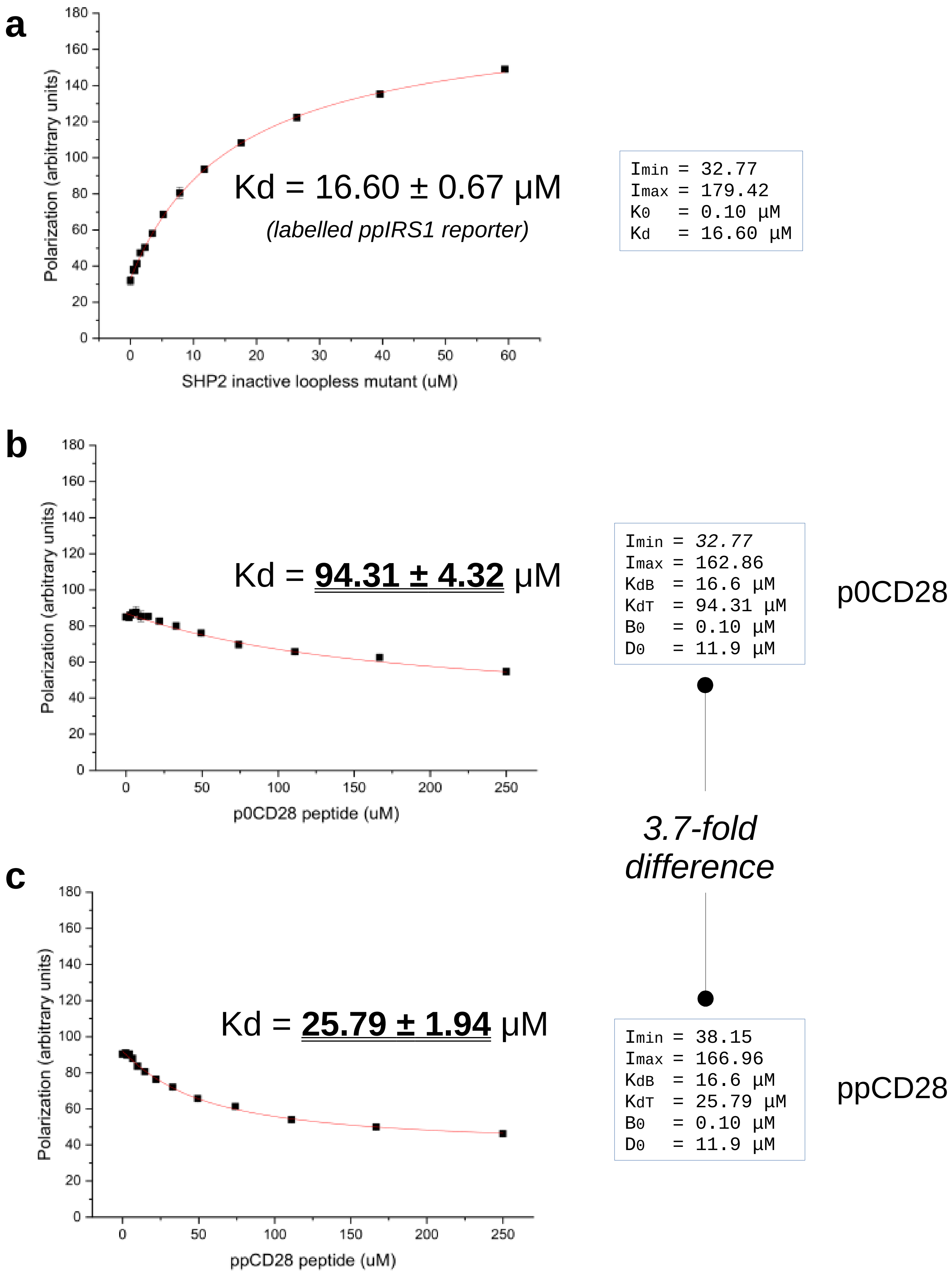

Supplementary figure 48. Loopless (R362G+K364S) inactive SHP2 direct (a) and competitive (b,c,) fluorescence polarization titrations (p0CD28 vs. ppCD28) (n=3 technical replicates, error bars show ±SD for each point). Source data are provided as a Source Data file.

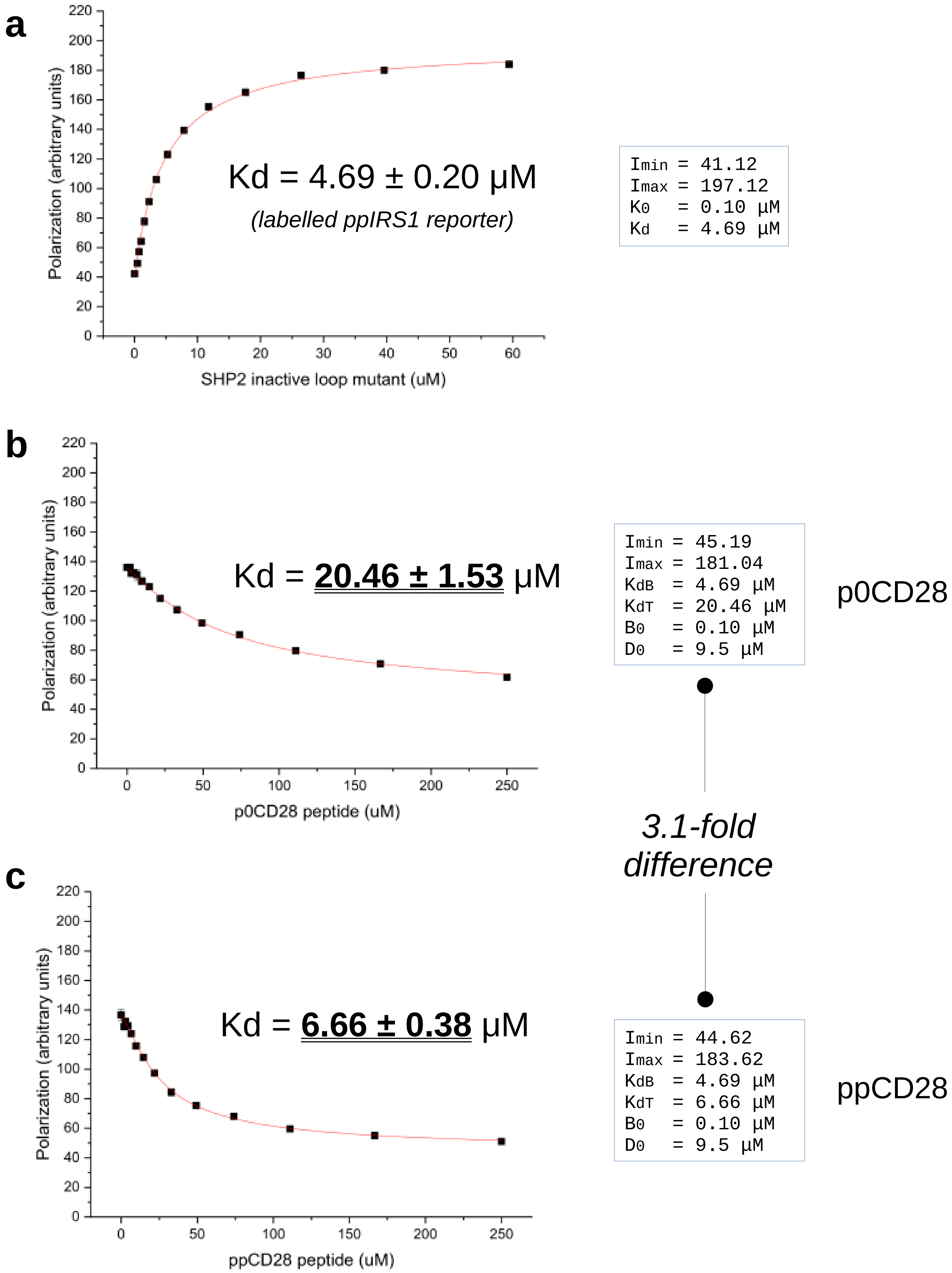

Supplementary figure 49. Loop mutant (K364E) inactive SHP2 direct (a) and competitive (b,c) fluorescence polarization titrations (p0CD28 vs. ppCD28) (n=3 technical replicates, error bars show ±SD for each point).
Source data are provided as a Source Data file.

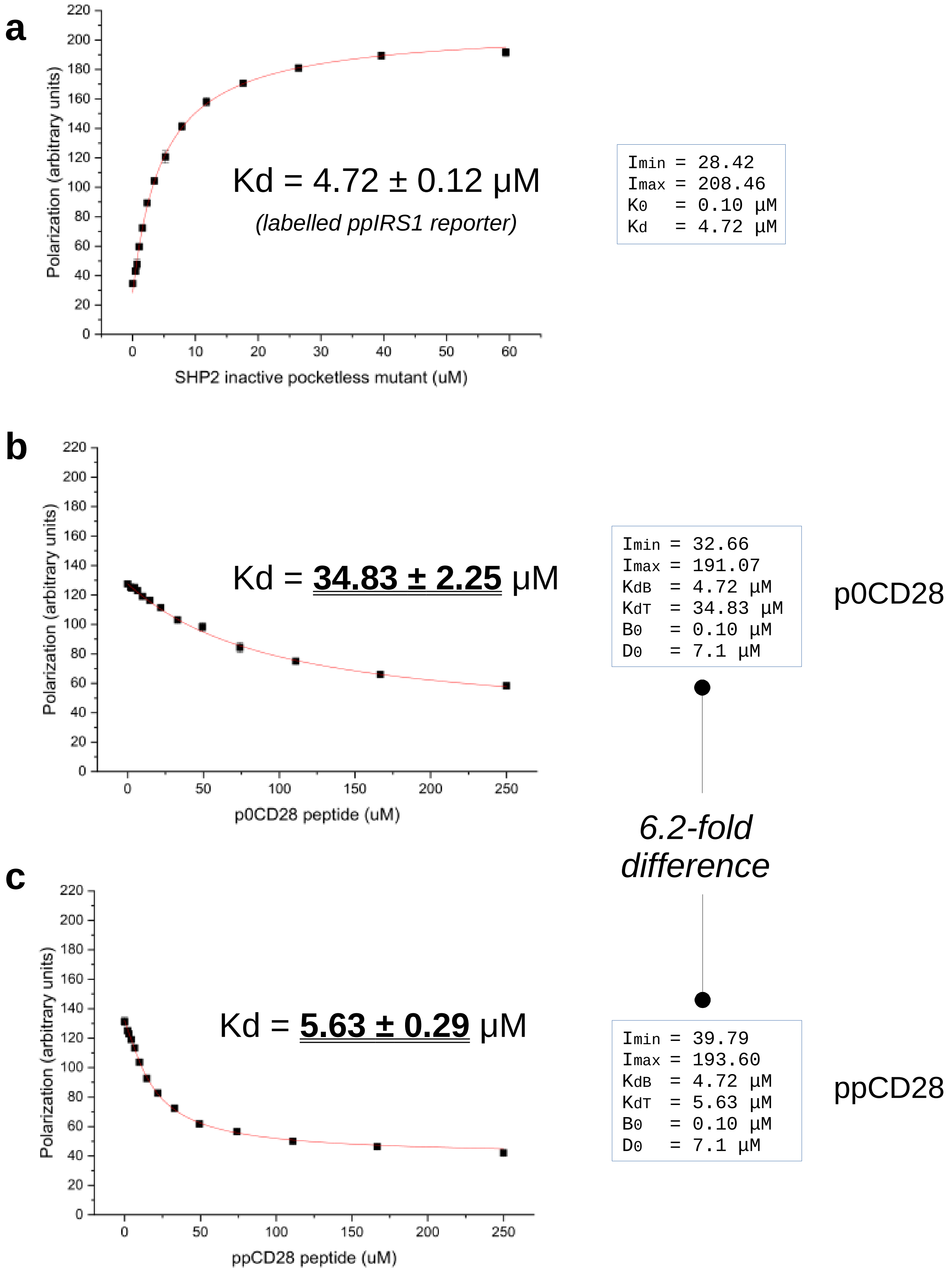

Supplementary figure 50. Pocketless (K260E+R265S) inactive SHP2 direct (a) and competitive (b,c) fluorescence polarization titrations (p0CD28 vs. ppCD28) (n=3 technical replicates, error bars show ±SD for each point). Source data are provided as a Source Data file.

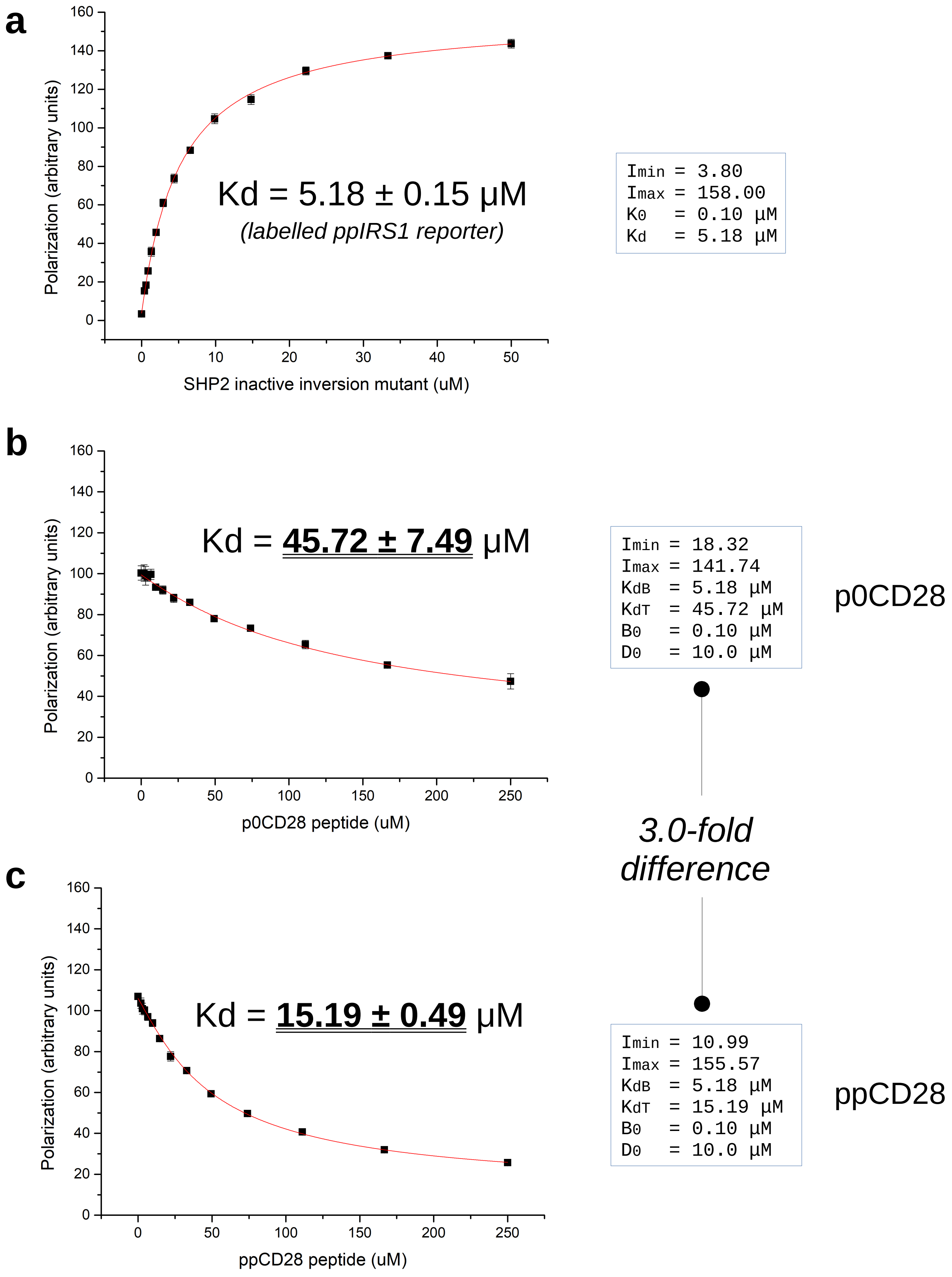

Supplementary figure 51. Loop inversion (R362E) mutant inactive SHP2 direct (a) and competitive (b,c) fluorescence polarization titrations (p0CD28 vs. ppCD28) (n=3 technical replicates, error bars show ±SD for each point). Source data are provided as a Source Data file.

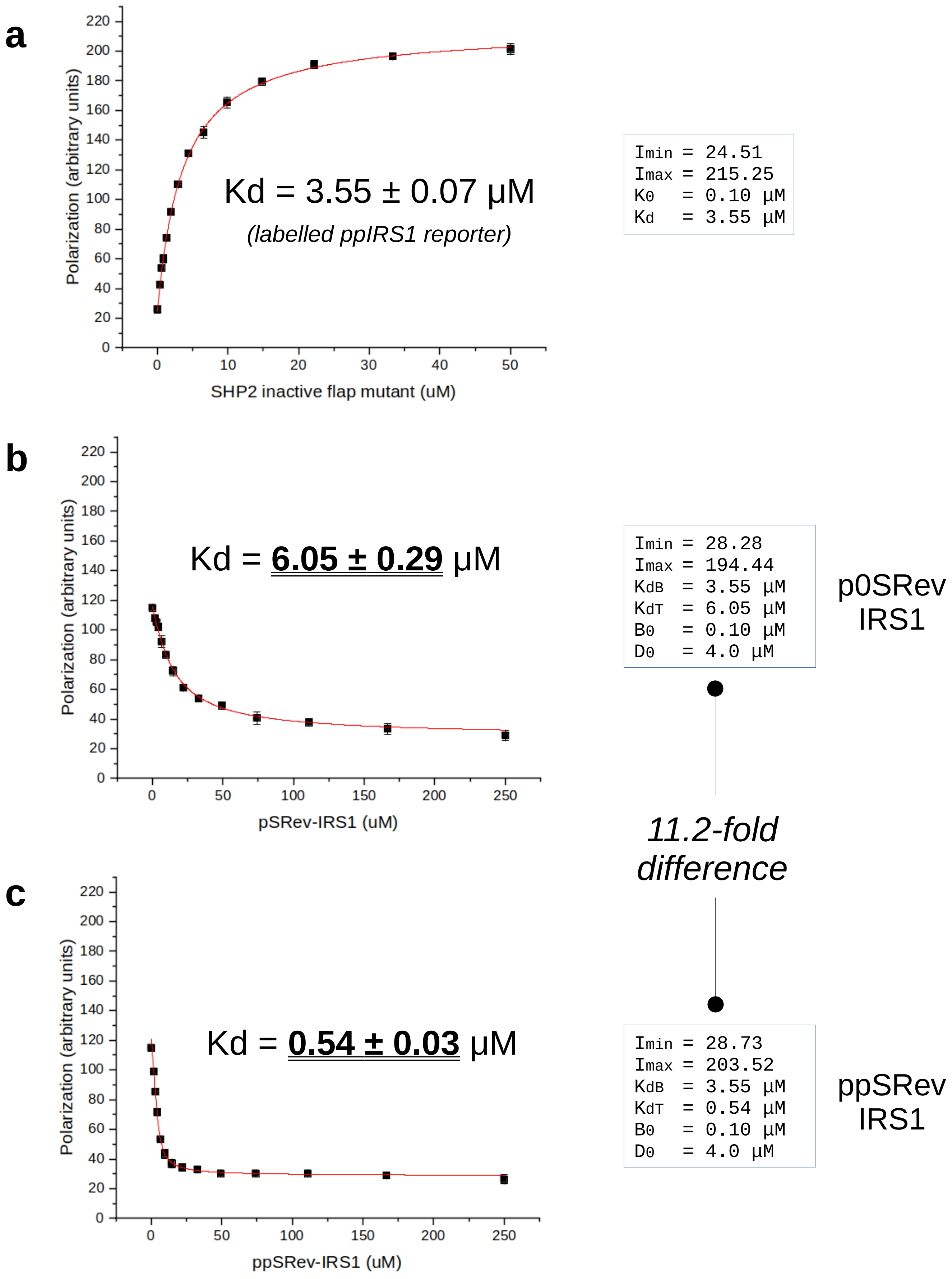

Supplementary figure 52. Flap mutant (H430F) inactive SHP2 direct (a) and competitive (b,c) fluorescence polarization titrations (p0SRev-IRS1 vs. ppSRev-IRS1) (n=3 technical replicates, error bars show ±SD for each point). Source data are provided as a Source Data file.

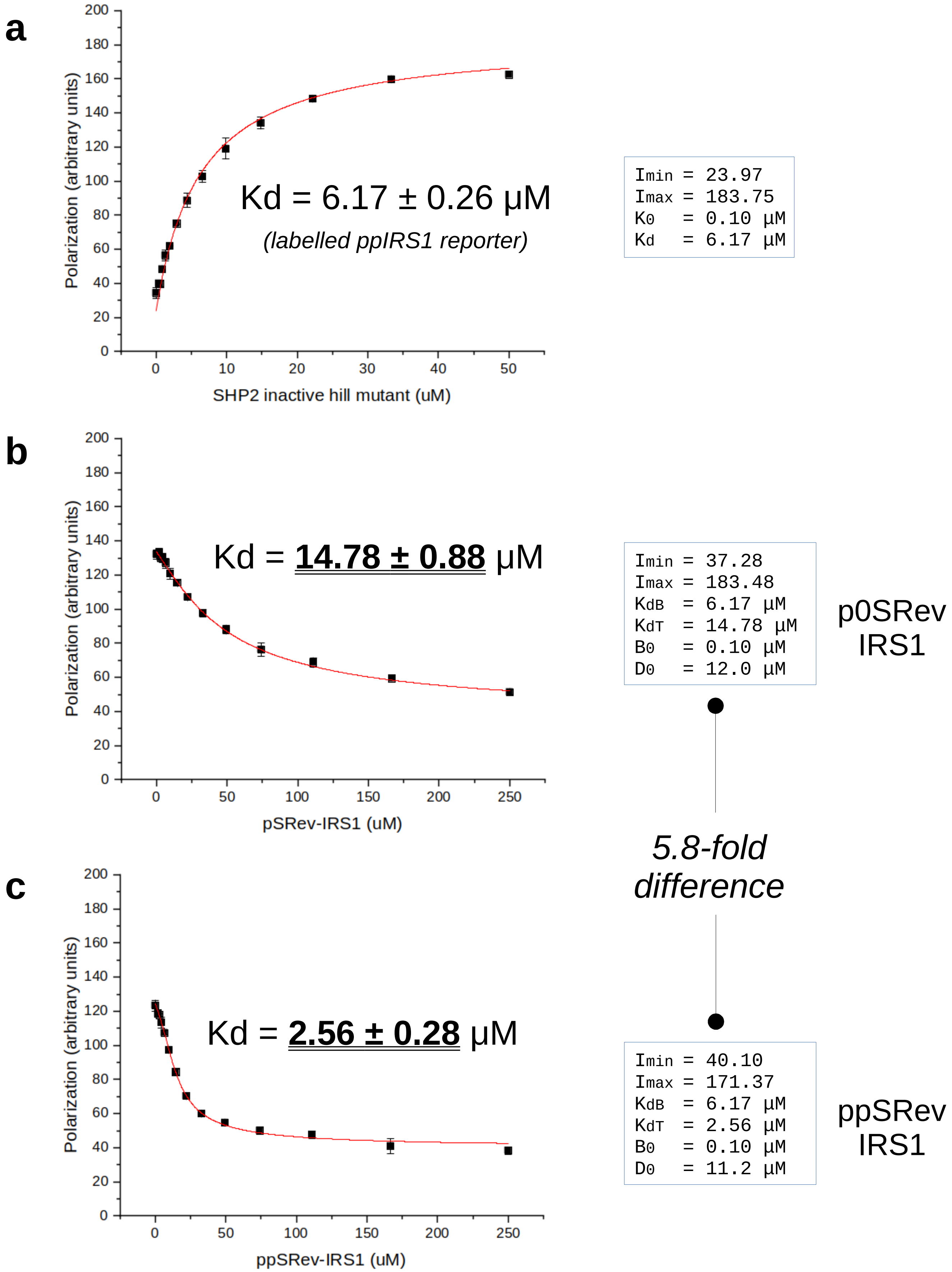

Supplementary figure 53. Hill mutant (K274E) inactive SHP2 direct (a) and competitive (b,c) fluorescence polarization titrations (p0SRev-IRS1 vs. ppSRev-IRS1) (n=3 technical replicates, error bars show ±SD for each point). Source data are provided as a Source Data file.

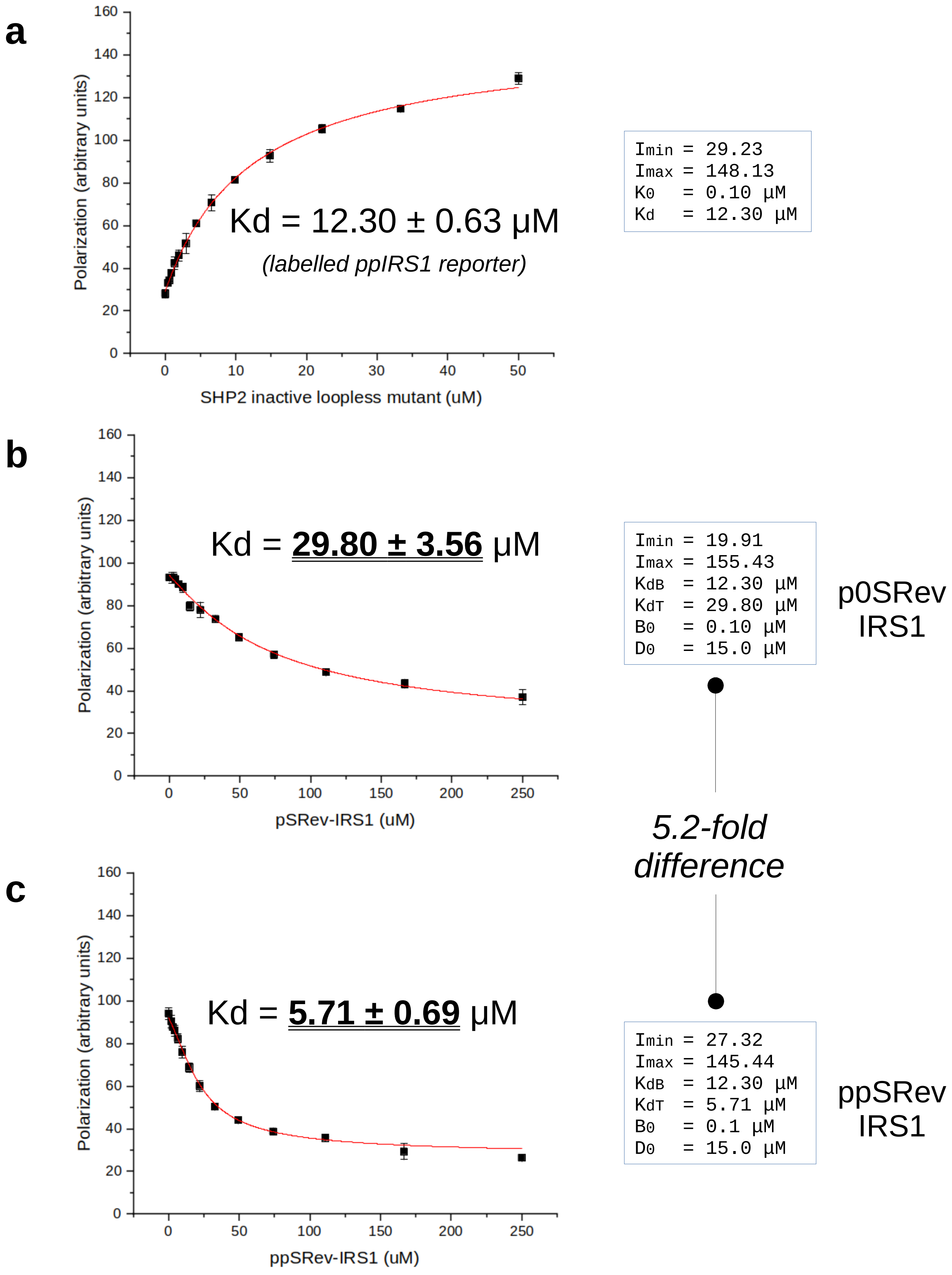

Supplementary figure 54. Loopless (R362G+K364S) inactive SHP2 direct (a) and competitive (b,c) fluorescence polarization titrations (p0SRev-IRS1 vs. ppSRev-IRS1) (n=3 technical replicates, error bars show ±SD for each point). Source data are provided as a Source Data file.

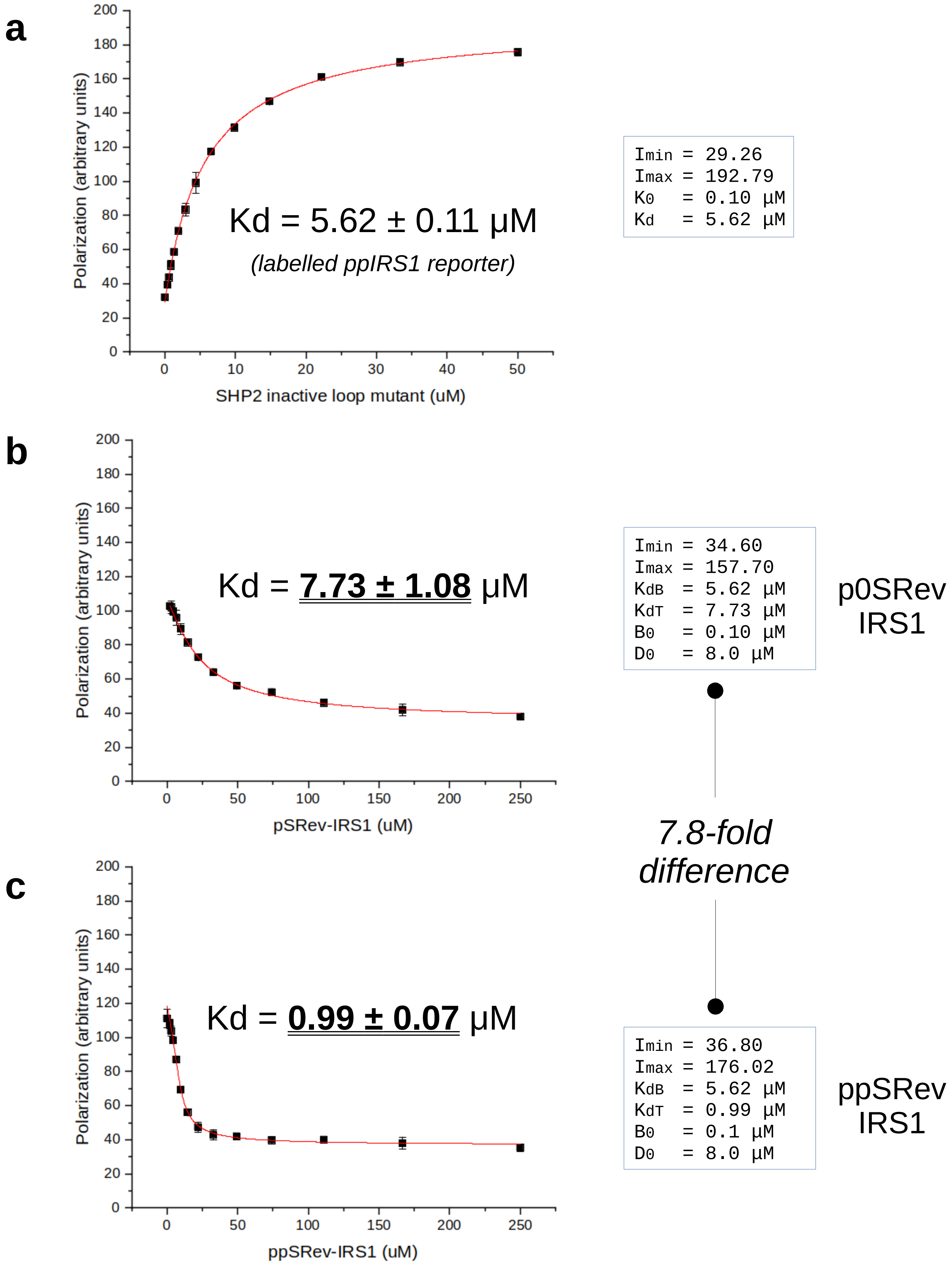

Supplementary figure 55. Loop mutant (K364E) inactive SHP2 direct (a) and competitive (b,c) fluorescence polarization titrations (p0SRev-IRS1 vs. ppSRev-IRS1) (n=3 technical replicates, error bars show ±SD for each point). Source data are provided as a Source Data file.

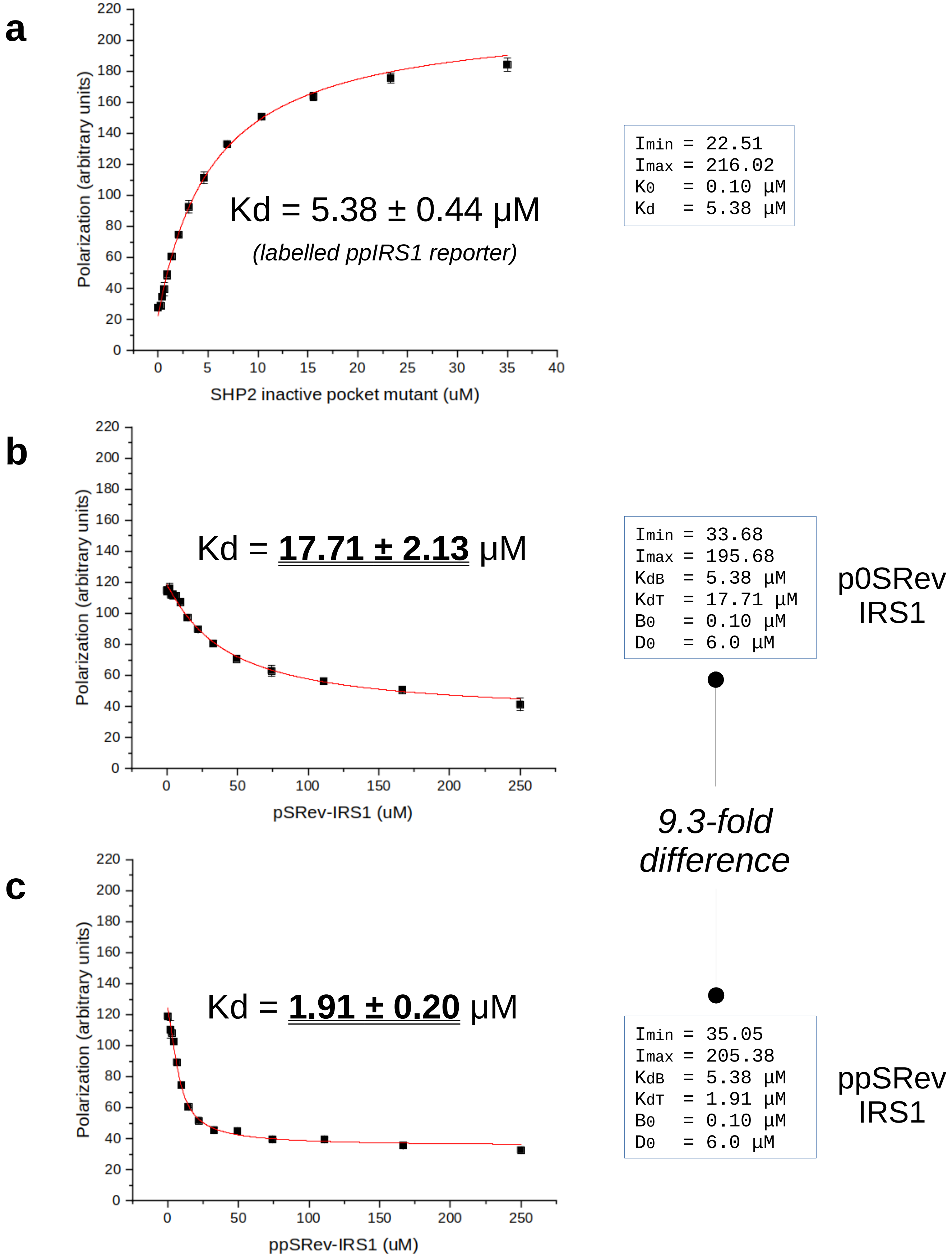

Supplementary figure 56. Pocketless (K260E+R265S) inactive SHP2 direct (a) and competitive (b,c) fluorescence polarization titrations (p0SRev-IRS1 vs. ppSRev-IRS1) (n=3 technical replicates, error bars show ±SD for each point). Source data are provided as a Source Data file.

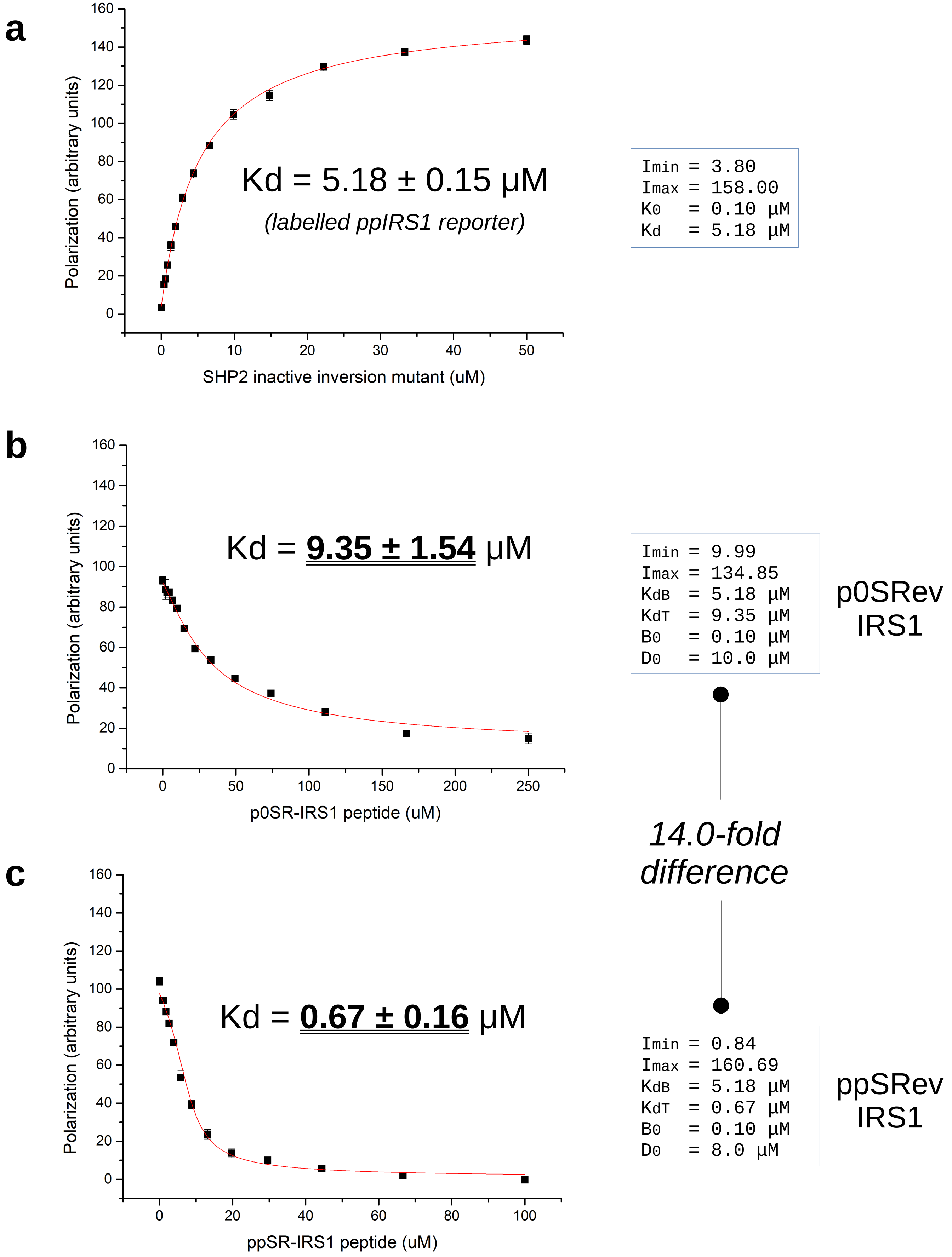

Supplementary figure 57. Loop inversion (R362E) mutant inactive SHP2 direct (a) and competitive (b,c) fluorescence polarization titrations (p0SRev-IRS1 vs. ppSRev-IRS1) (n=3 technical replicates, error bars show ±SD for each point). Source data are provided as a Source Data file.

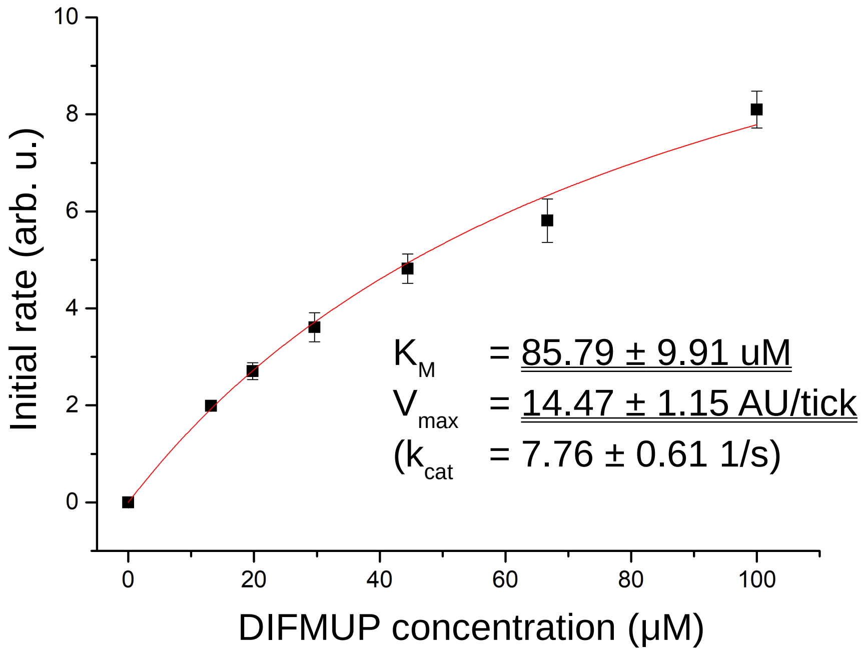

Supplementary figure 58. Wild-type SHP2 catalytic domain kinetics with DIFMUP (n=3 technical repeats, 2.5 nM enzyme, fitted to 60 points [10 min], showing ±SD). Source data are provided as a Source Data file.

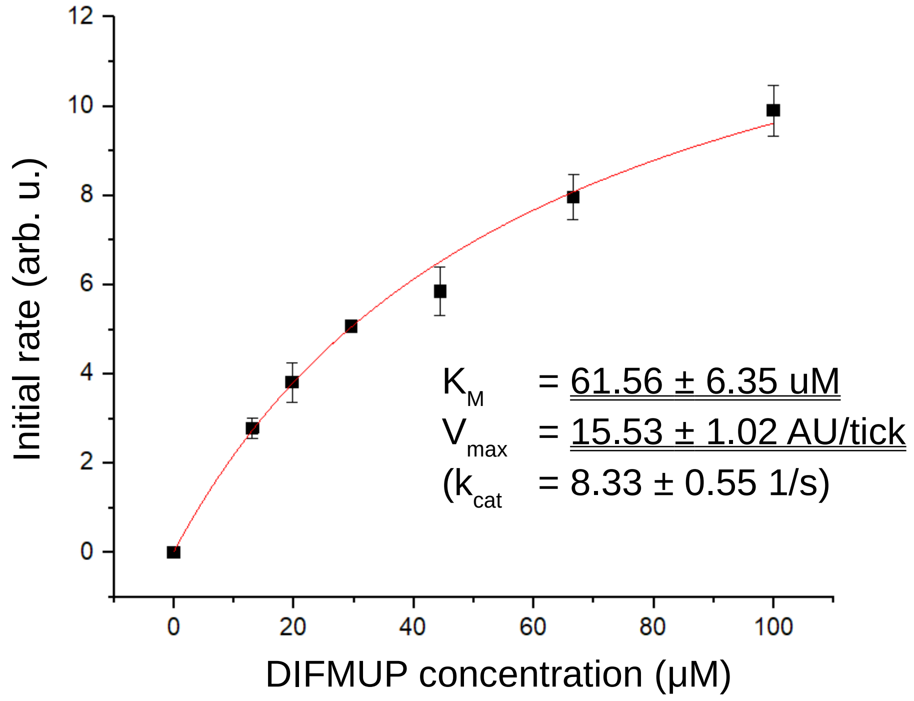

Supplementary figure 59. Active SHP2 crystallization construct (Δ(219-245)-Δ(315-323)+GSSG) kinetics with DIFMUP (n=3 technical repeats, 2.5 nM enzyme, fitted to 60 points [10 min], showing ±SD). Source data are provided as a Source Data file.

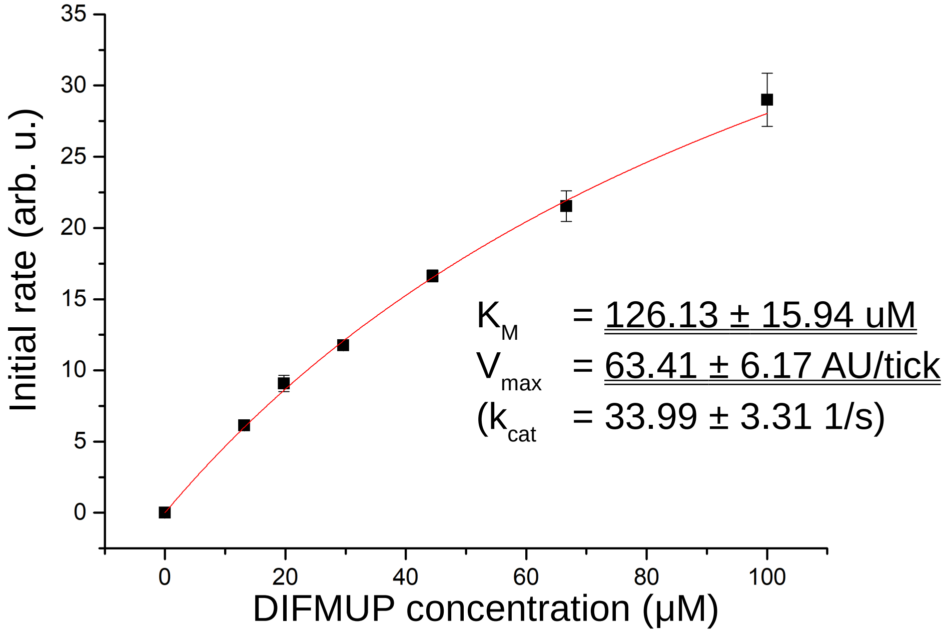

Supplementary figure 60. Active SHP2 loop mutant (K364E) kinetics with DIFMUP (n=3 technical repeats, 2.5 nM enzyme, fitted to 30 points [5 min], showing ±SD). Source data are provided as a Source Data file.

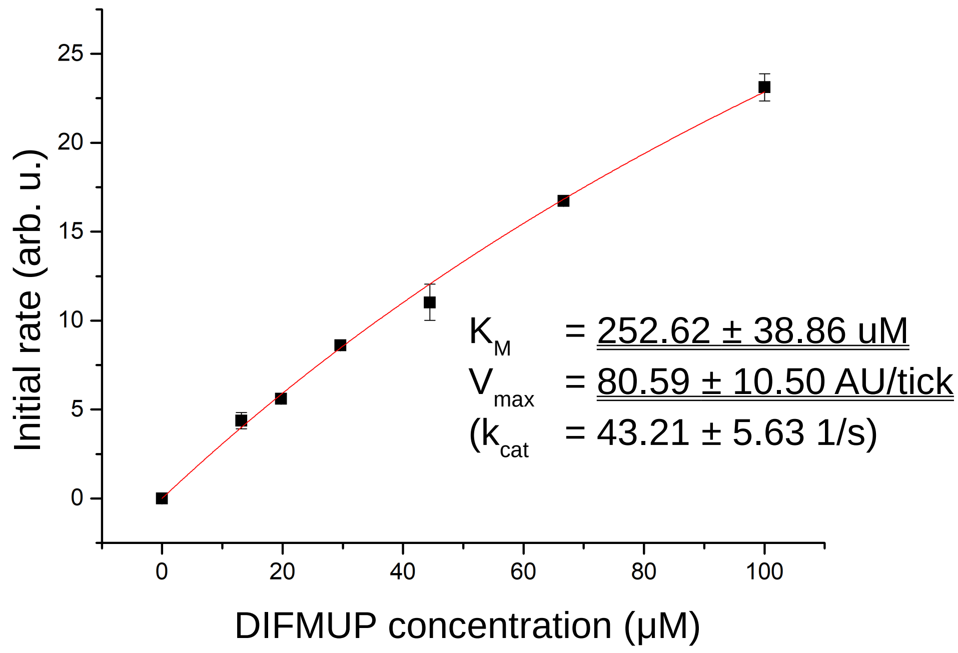

Supplementary figure 61. Active SHP2 loopless (R362G+K364S) kinetics with DIFMUP (n=3 technical repeats, 2.5 nM enzyme, fitted to 30 points [5 min], showing ±SD). Source data are provided as a Source Data file.

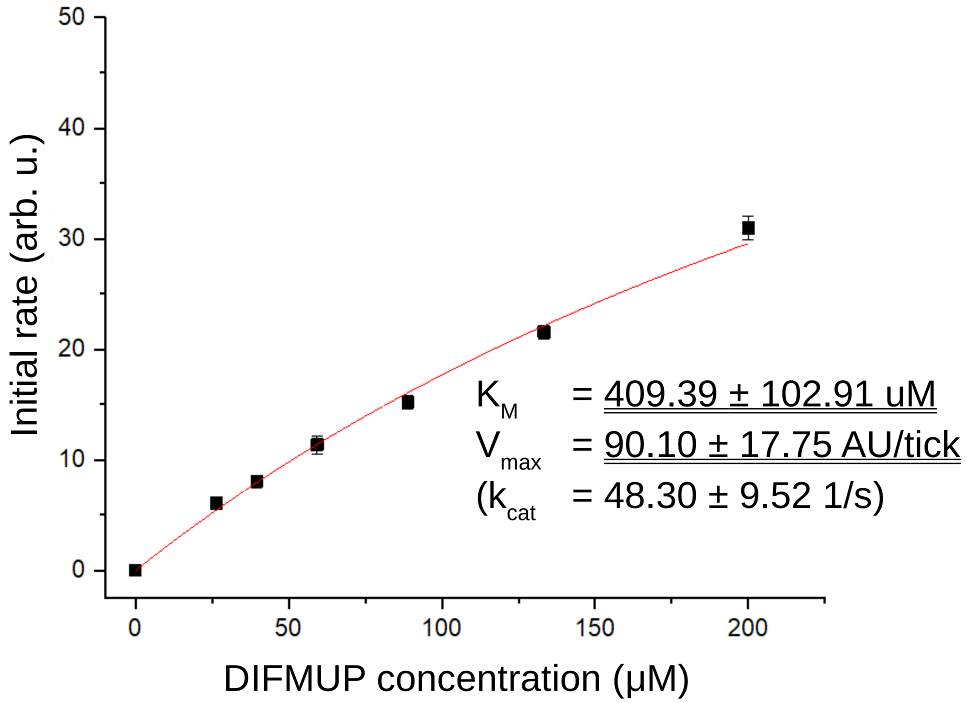

Supplementary figure 62. Active SHP2 loop inversion (R362E) kinetics with DIFMUP (n=3 technical repeats, 2.5 nM enzyme, fitted to 30 points [5 min], showing ±SD). Source data are provided as a Source Data file.

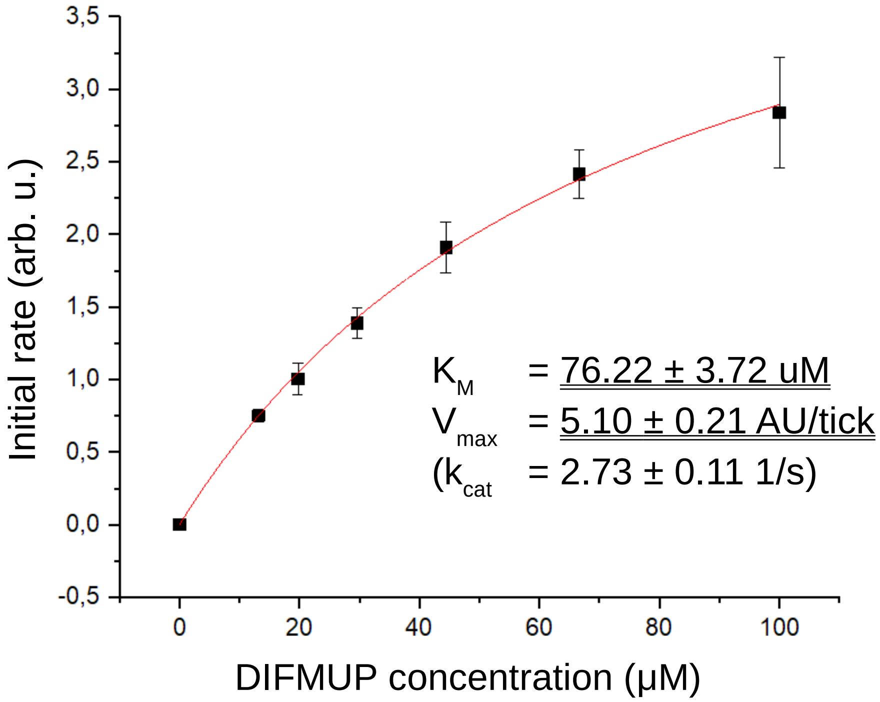

Supplementary figure 63. H426A catalytic mutant SHP2 kinetics with DIFMUP (n=3 technical repeats, 2.5 nM enzyme, fitted to 60 points [10 min], showing ±SD). Source data are provided as a Source Data file.

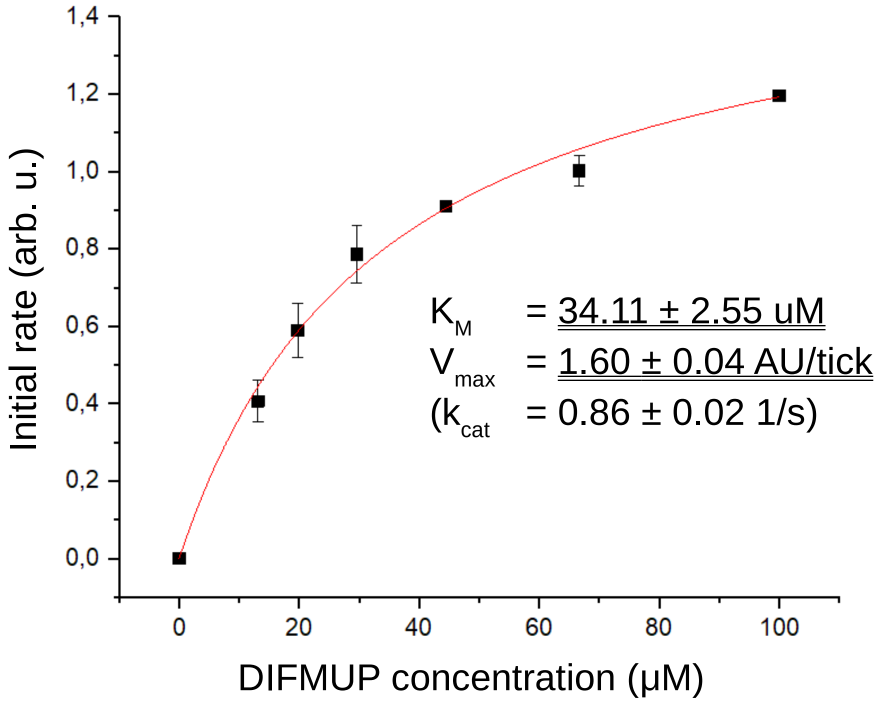

Supplementary figure 64. D425A catalytic mutant SHP2 kinetics with DIFMUP (n=3 technical repeats, 12.5 nM enzyme, fitted to 30 points [5 min] , showing ±SD). Initial rates were adjusted by 1/5 as if 2.5 nM enzyme were used). Source data are provided as a Source Data file.

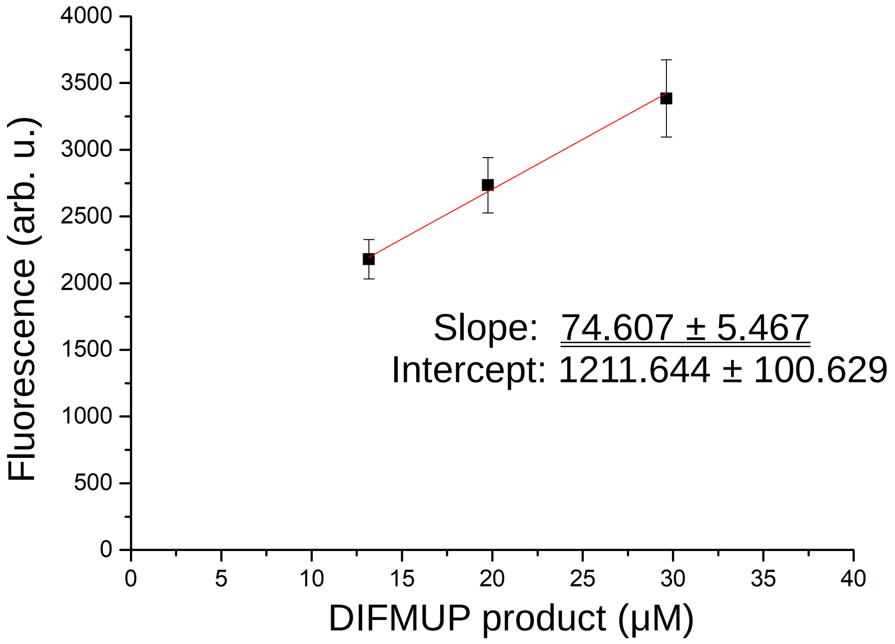

k_{cat} [1/s] calculation: V_{max} [AU/tick] * 1 / 74.61[AU/uM] * 1 / 10[s/tick] * 1 / 0.0025[uM enzyme]

Supplementary figure 65. DIFMUP product fluorescence calibration (linear fit to the lowest calibration points). From a total of n=10 independent experiments (derived from n=3 technical repats each, error bars show ±SD) for a total of 30 points. Reaction was at endpoint after 10 mins after applying undiluted (1mM+) enzyme. Source data are provided as a Source Data file.

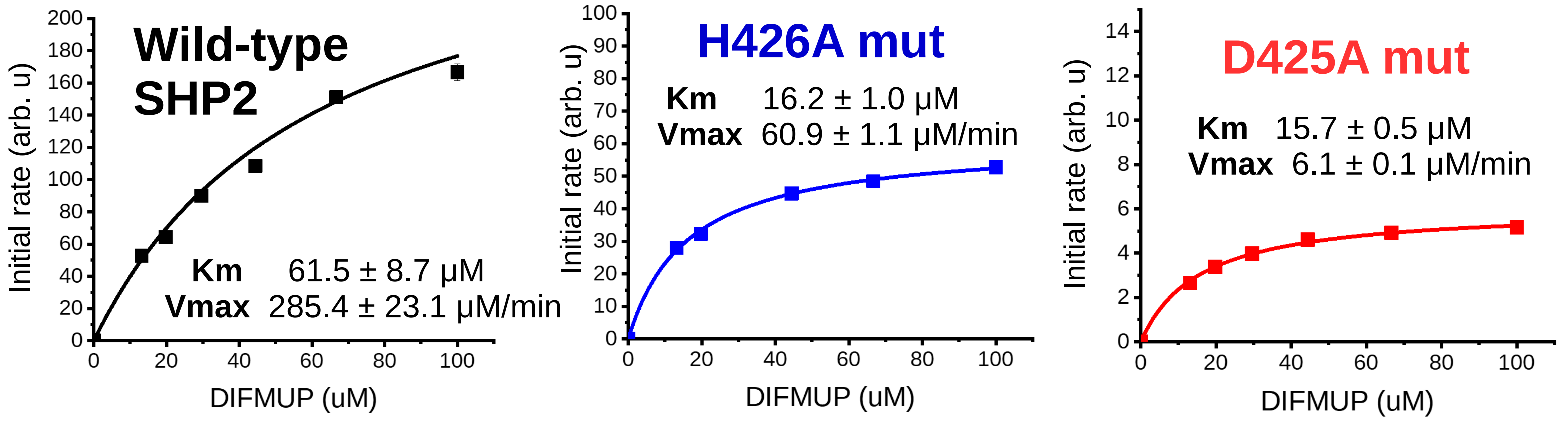

Supplementary figure 66. Additional Michaelis-menten kinetics of SHP2 surface mutants on the small molecule substrate DIFMUP, presented for the reproducibility of results. These curves were fitted to n=3 technical replicates, using a different protocol (1 min read intervals, slope fitting to 3 initial points) and instrument (BioTek plate reader), than for curves presented on Figure #7. Source data are provided as a Source Data file.

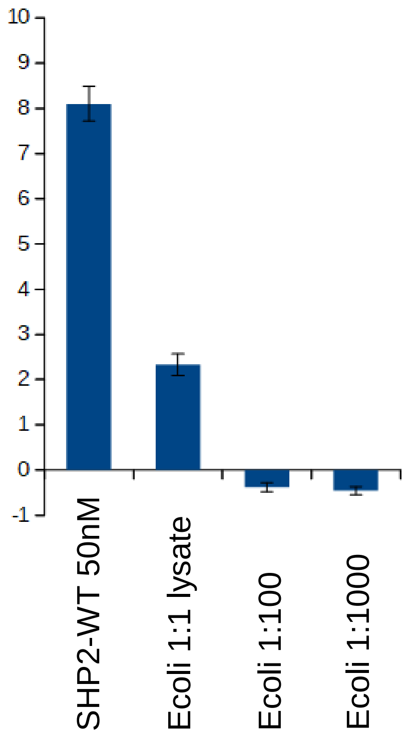

Activity @ 100 uM DIFMUP concentration:
(2 μl sample to 40 μl reaction mix)

Undiluted lysate: 2.33 ± 0.24 units / 10 sec
1/100 dilution: -0.38 ± 0.10 units / 10 sec
1/1000 dilution: -0.46 ± 0.09 units / 10 sec

WT SHP2 50nM: 8.10 ± 0.38 units / 10 sec
(diluted over 1/1000 from protein stock)

Supplementary figure 67. Background phosphatase activity of E. coli lysates. Initial rates were fitted to 30 data points with n=3 technical repeats each (error bars show ±SD). Source data are provided as a Source Data file.

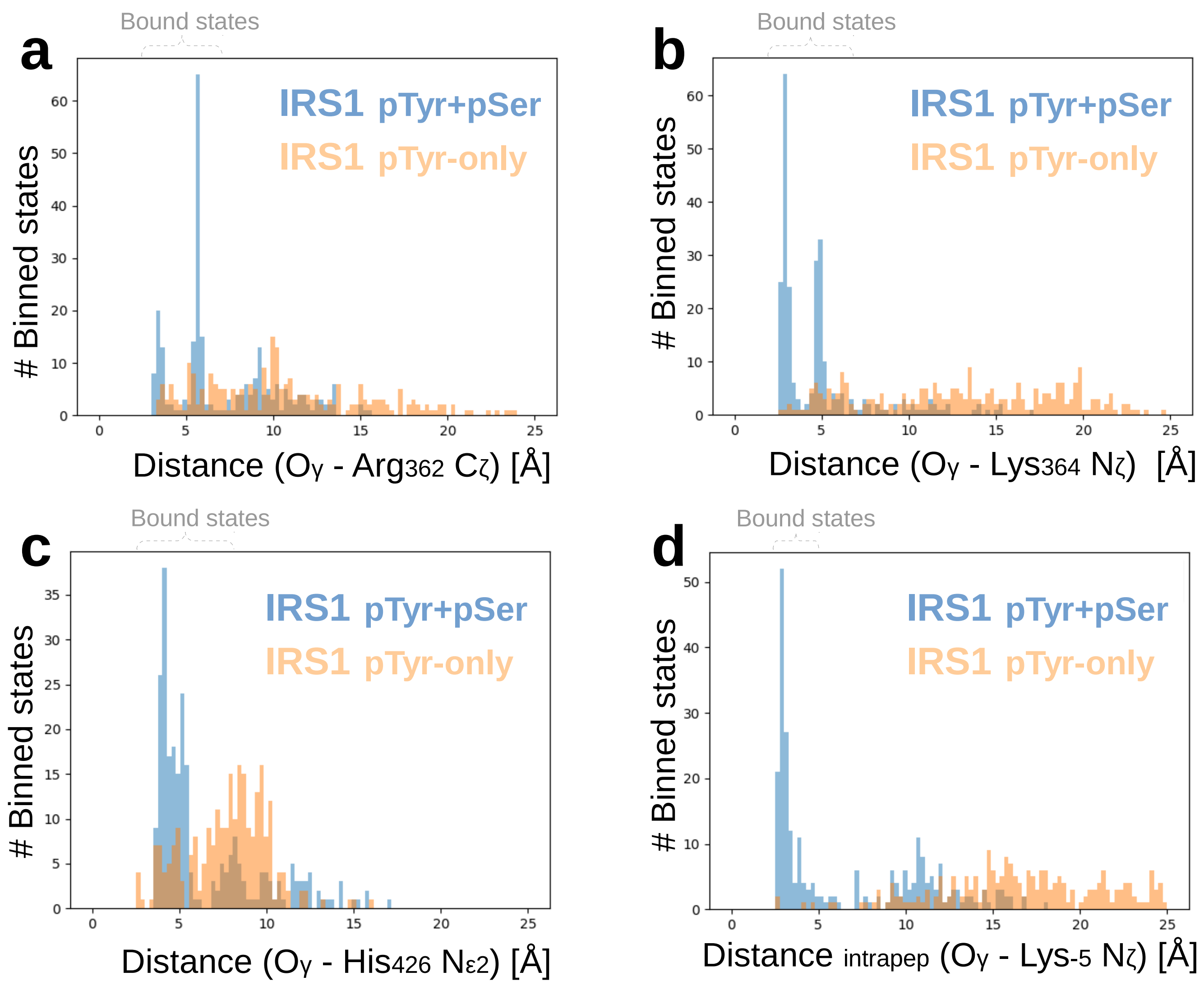

Supplementary Figure 68. Characteristic substrate-enzyme (a,b,c) and intra-substrate (d) distances between the phosphorylated side chain (oxygen atom) and neighbouring atoms, observed during molecular dynamics (MD) simulations. A total of 5 independent simulations were performed for each complex with GROMACS (ppIRS1, p0IRS1, ppCD28, p0CD28, ppRev-IRS1, p0Rev-IRS1), using slightly different input models for each run, with a t=10 ns time (after equlibration) and explicit waters. Distances in the resulting smoothened models (n=51 states for each model) were then plotted as histograms. Although no dissociation events were observed during MD, from these analyses, it is obvious that the unmodified termini of substrate peptides are much more mobile than phosphorylated ones. The pSer/pThr in the ppIRS1 peptide tends to bind to one region of SHP2. Source data are provided as a Source Data file.

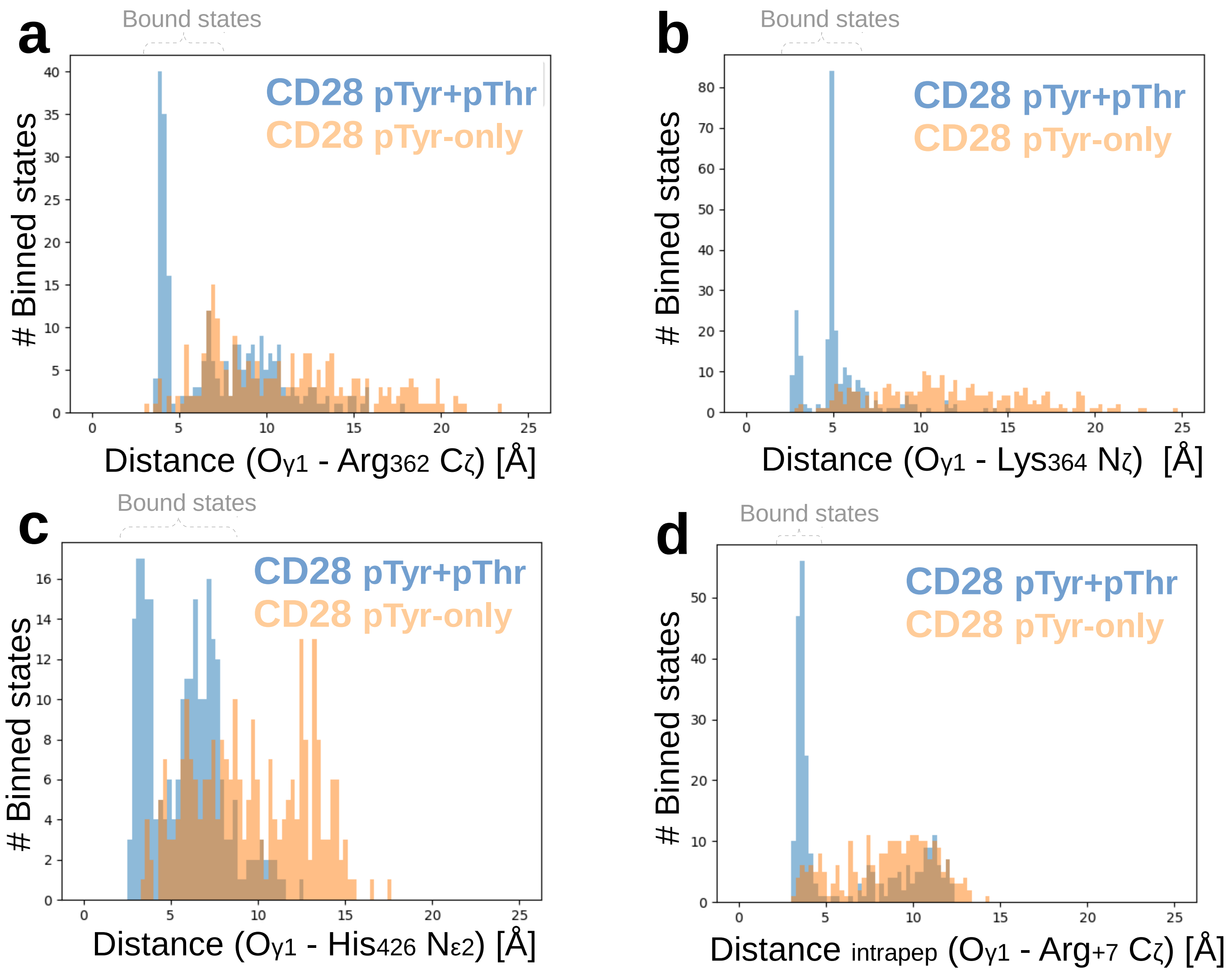

Supplementary Figure 69. Characteristic substrate-enzyme (a,b,c) and intra-substrate (d) distances between the phosphorylated side chain (oxygen atom) and neighbouring atoms, observed during molecular dynamics (MD) simulations. A total of 5 independent simulations were performed for each complex with GROMACS (ppIRS1, p0IRS1, ppCD28, p0CD28, ppRev-IRS1, p0Rev-IRS1), using slightly different input models for each run, with a t=10 ns time (after equlibration) and explicit waters. Distances in the resulting smoothened models (n=51 states for each model) were then plotted as histograms. Although no dissociation events were observed during MD, from these analyses, it is obvious that the unmodified termini of substrate peptides are much more mobile than phosphorylated ones. The pSer/pThr in the ppCD28 peptide tends to bind to one region of SHP2. Source data are provided as a Source Data file.

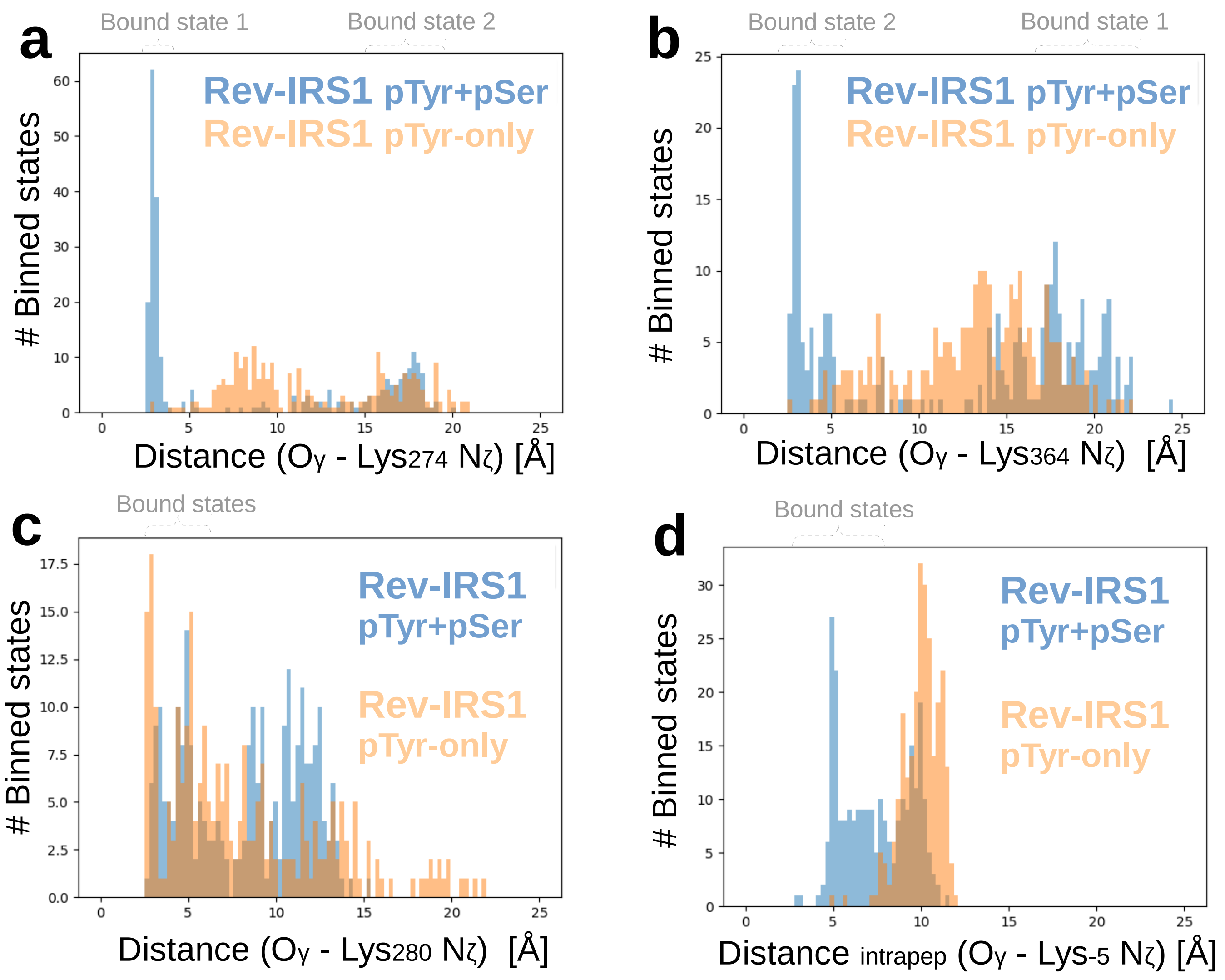

Supplementary Figure 70. Characteristic substrate-enzyme (a,b,c) and intra-substrate (d) distances between the phosphorylated side chain (oxygen atom) and neighbouring atoms, observed during molecular dynamics (MD) simulations. A total of 5 independent simulations were performed for each complex with GROMACS (ppIRS1, p0IRS1, ppCD28, p0CD28, ppRev-IRS1, p0Rev-IRS1), using slightly different input models for each run, with a t=10 ns time (after equlibration) and explicit waters. Distances in the resulting smoothened models (n=51 states for each model) were then plotted as histograms. Although no dissociation events were observed during MD, from these analyses, it is obvious that the unmodified termini of substrate peptides are much more mobile than phosphorylated ones. The pSer in the ppRev-IRS1 peptide alternates between two, equally possible binding sites. Source data are provided as a Source Data file.

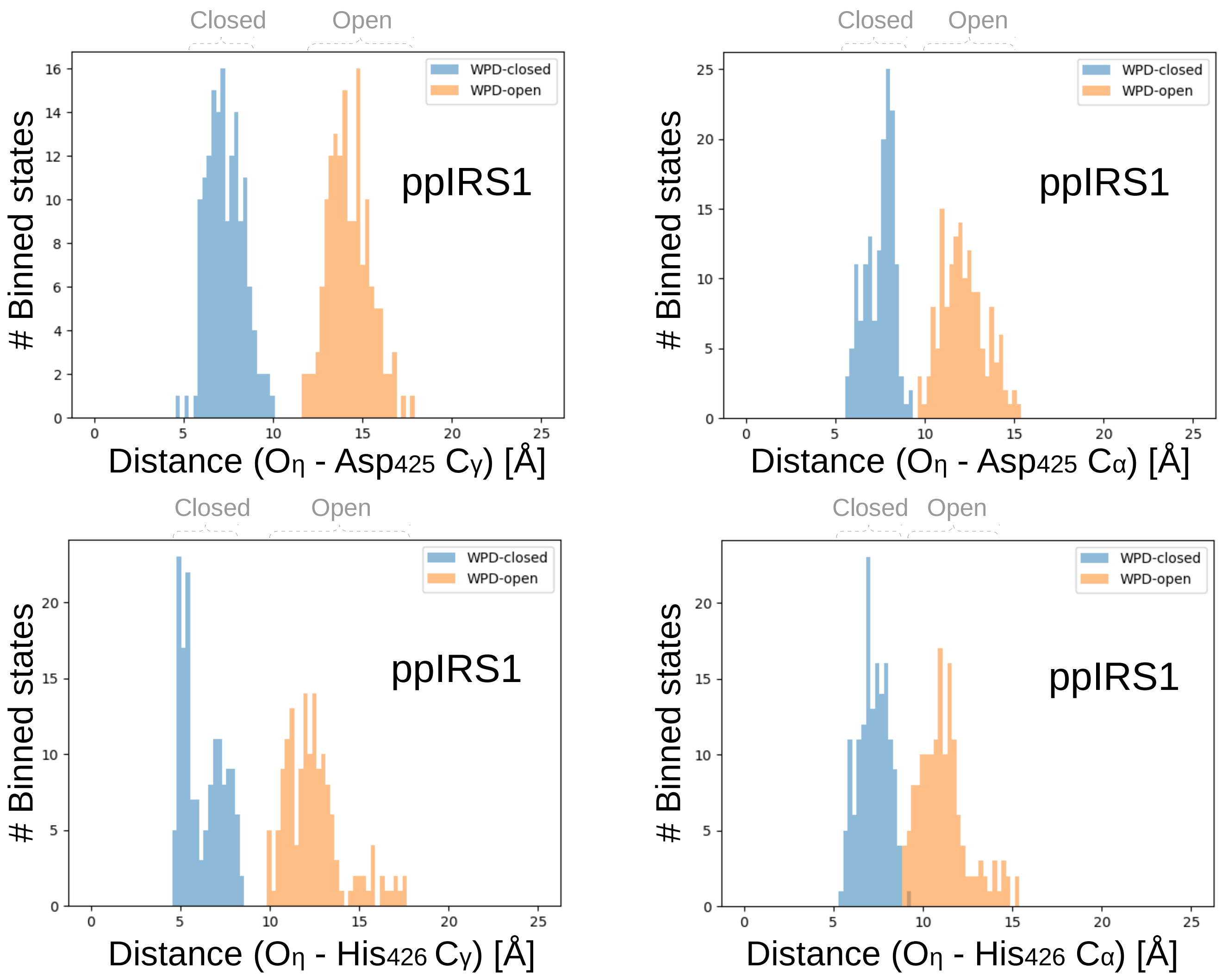

Supplementary Figure 71. Comparison of characteristic distances in WPD-closed vs. WPD-open (regular) SHP2-substrate complex models. To track the movement of WPD loops, we selected the Cα and Cγ atoms of Asp425 and His426, and calculated their distance from the η oxygen atom of tyrosine. Although these distances fluctuate in both closed and open models, they are typically present in a characteristic distribution. Since we had 5 models available for the WPD-open conformation, and only 3 for WPD-closed, we applied a random sampling procedure of the former states to equalize histograms for proper comparison (see Supplementary note 3). Source data are provided as a Source Data file.

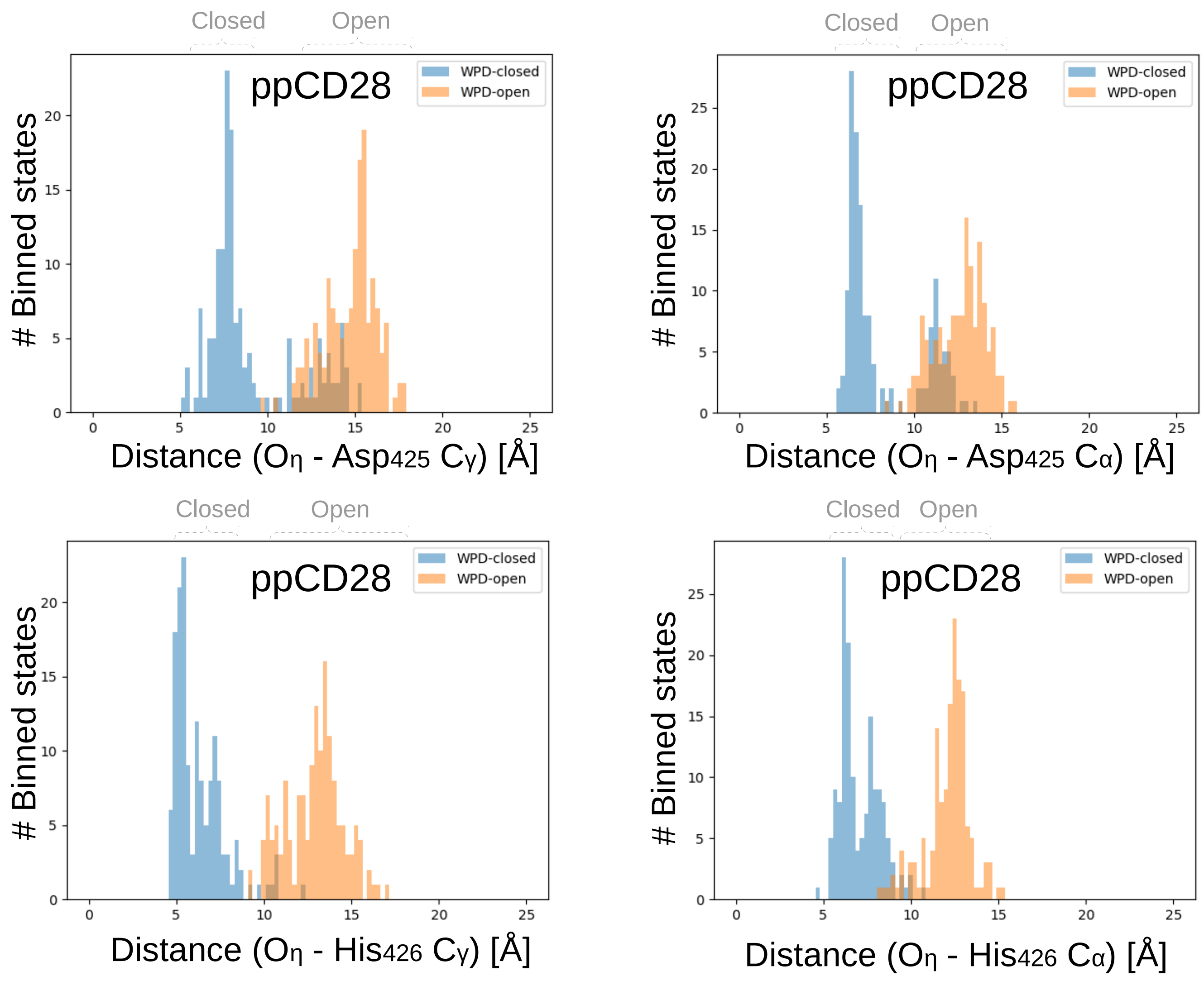

Supplementary Figure 72. Comparison of characteristic distances in WPD-closed vs. WPD-open (regular) SHP2-substrate complex models. To track the movement of WPD loops, we selected the Cα and Cγ atoms of Asp425 and His426, and calculated their distance from the η oxygen atom of tyrosine. Although these distances fluctuate in both closed and open models, they are typically present in a characteristic distribution. Observing a number of points during WPD-closed simulations in the WPD-open distribution suggests that this loop (temporarily) opens up in some of the runs. Since we had 5 models available for the WPD-open conformation, and only 3 for WPD-closed, we applied a random sampling procedure of the former states to equalize histograms for proper comparison (see Supplementary note 3). Source data are provided as a Source Data file.

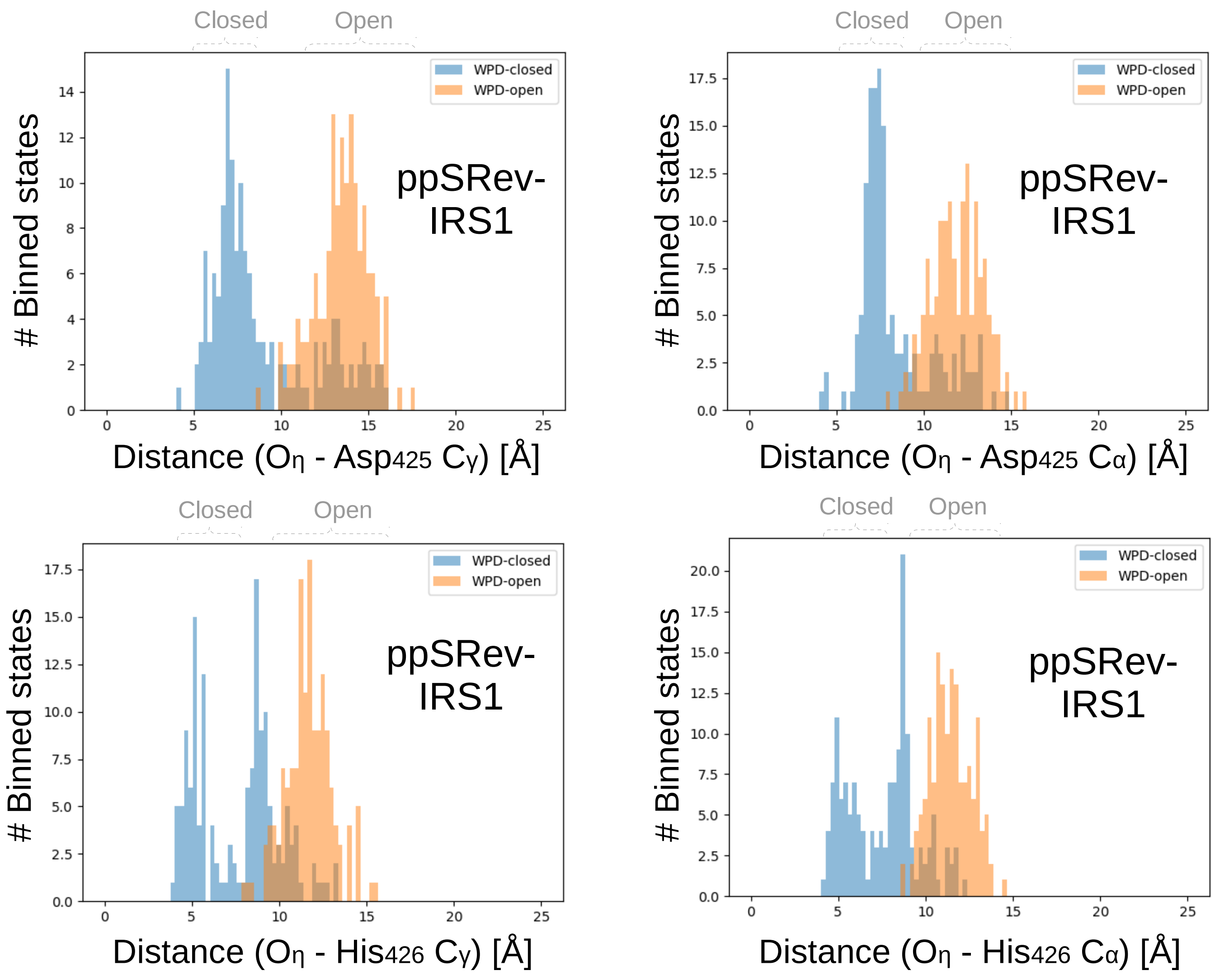

Supplementary figure 73. Comparison of characteristic distances in WPD-closed vs. WPD-open (regular) SHP2-substrate complex models. To track the movement of WPD loops, we selected the Cα and Cγ atoms of Asp425 and His426, and calculated their distance from the η oxygen atom of tyrosine. Although these distances fluctuate in both closed and open models, they are typically present in a characteristic distribution. Observing a number of points during WPD-closed simulations in the WPD-open distribution suggests that this loop (temporarily) opens up in some of the runs. Since we had 5 models available for the WPD-open conformation, and only 3 for WPD-closed, we applied a random sampling procedure of the former states to equalize histograms for proper comparison (Supplementary note 3). Source data are provided as a Source Data file.

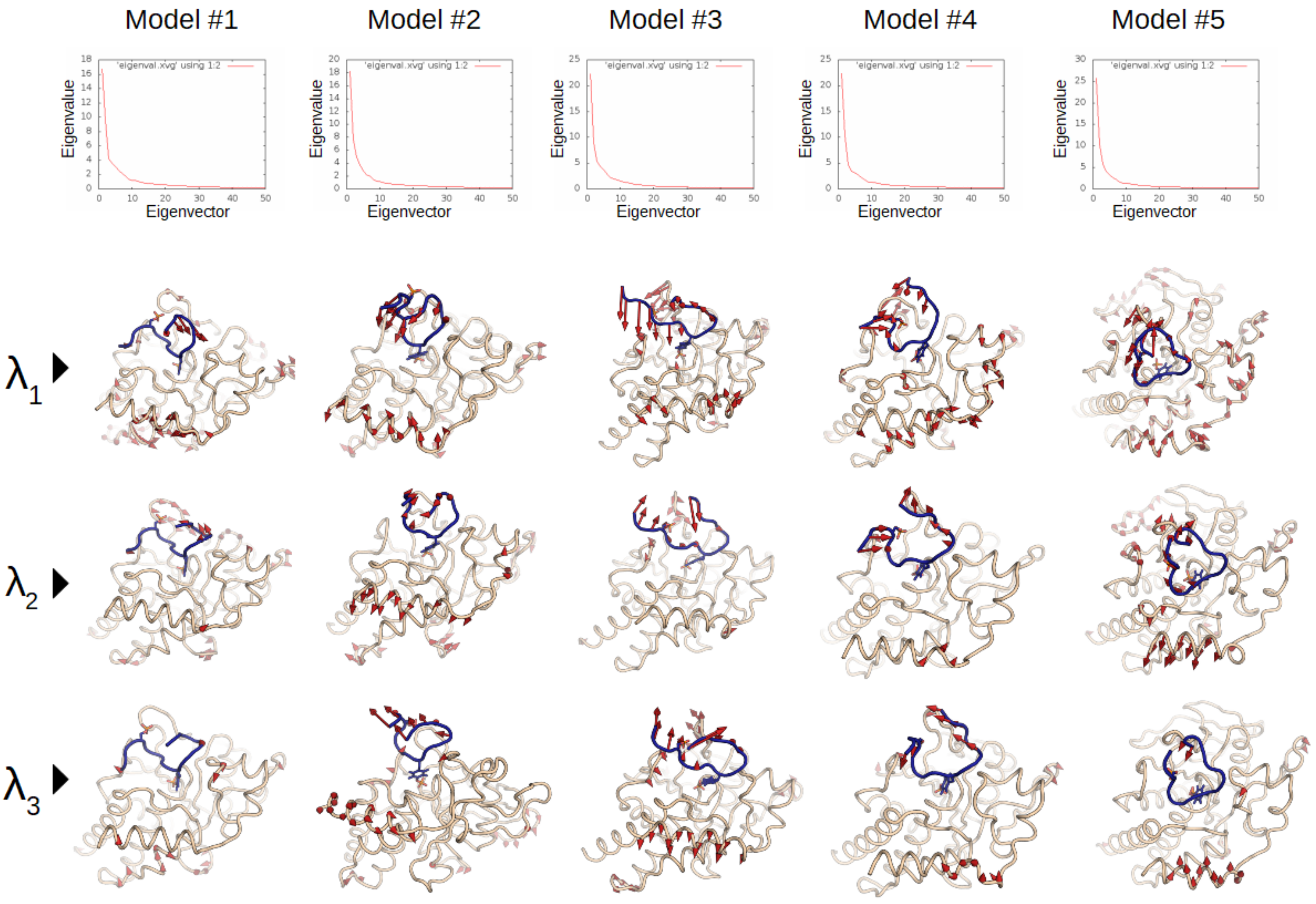

Supplementary figure 74. Principal component analysis for SHP2-ppIRS1 substrate-peptide complex molecular dynamics simulations. As they account for most mobility, only the movements for the 3 largest eigenvalues are shown for each simulation. Vectors were drawn using the “modevectors” PyMol extension.

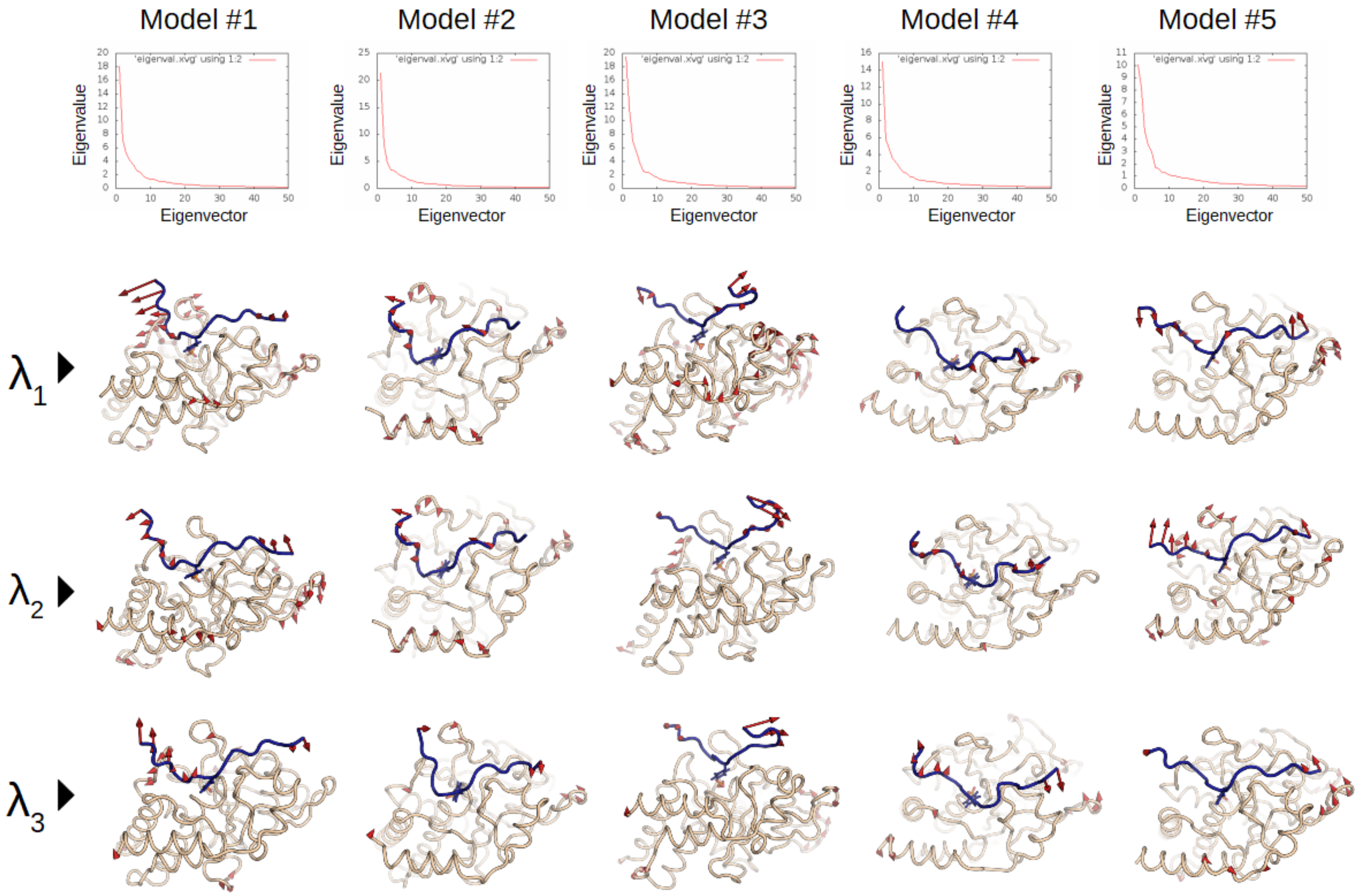

Supplementary figure 75. Principal component analysis for SHP2-ppCD28 substrate-peptide complex molecular dynamics simulations. As they account for most mobility, only the movements for the 3 largest eigenvalues are shown for each simulation. Vectors were drawn using the “modevectors” PyMol extension.

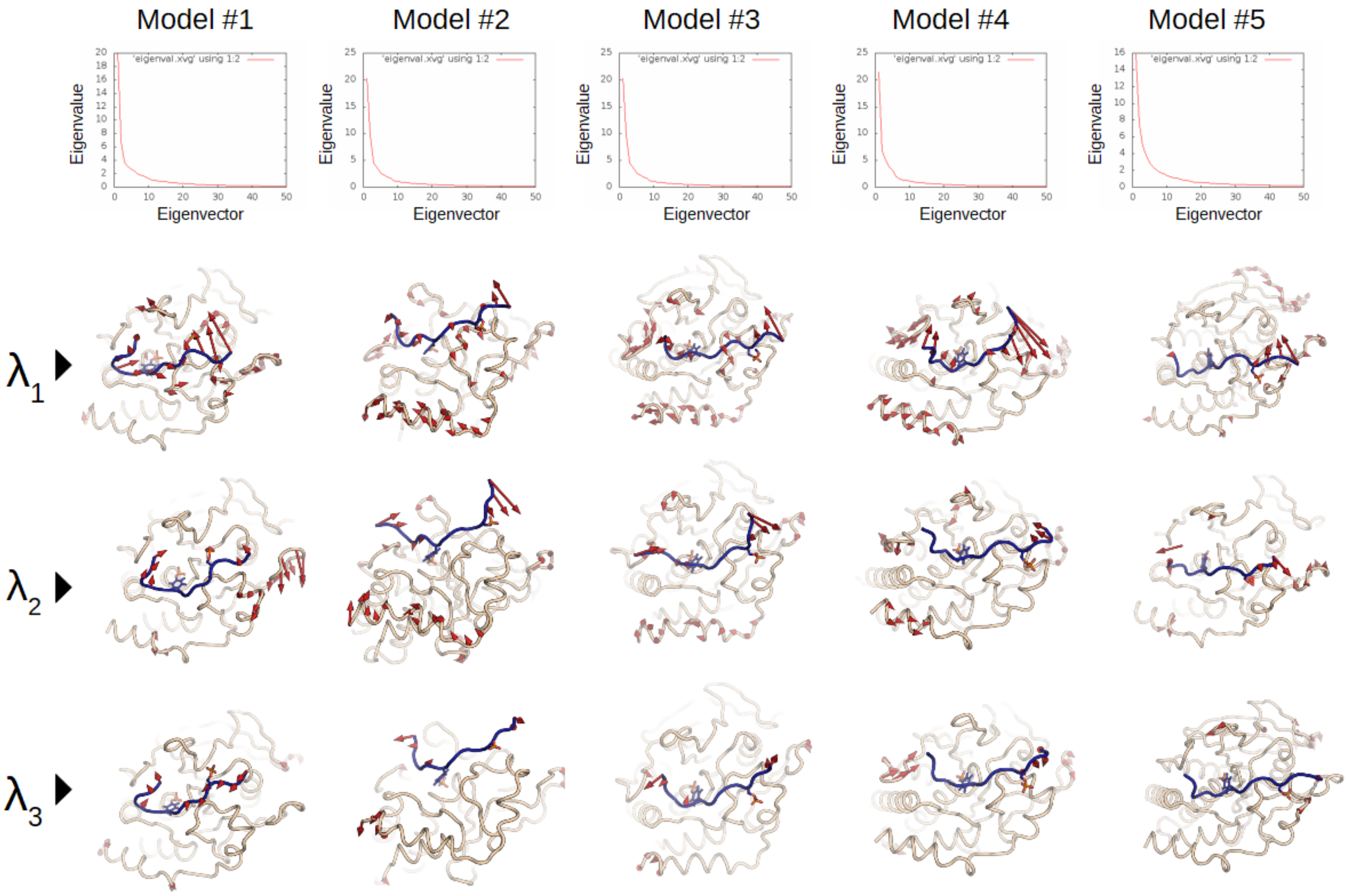

Supplementary figure 76. Principal component analysis for SHP2-ppSRev-IRS1 substrate-peptide complex molecular dynamics simulations. As they account for most mobility, only the movements for the 3 largest eigenvalues are shown for each simulation. Vectors were drawn using the “modevectors” PyMol extension.

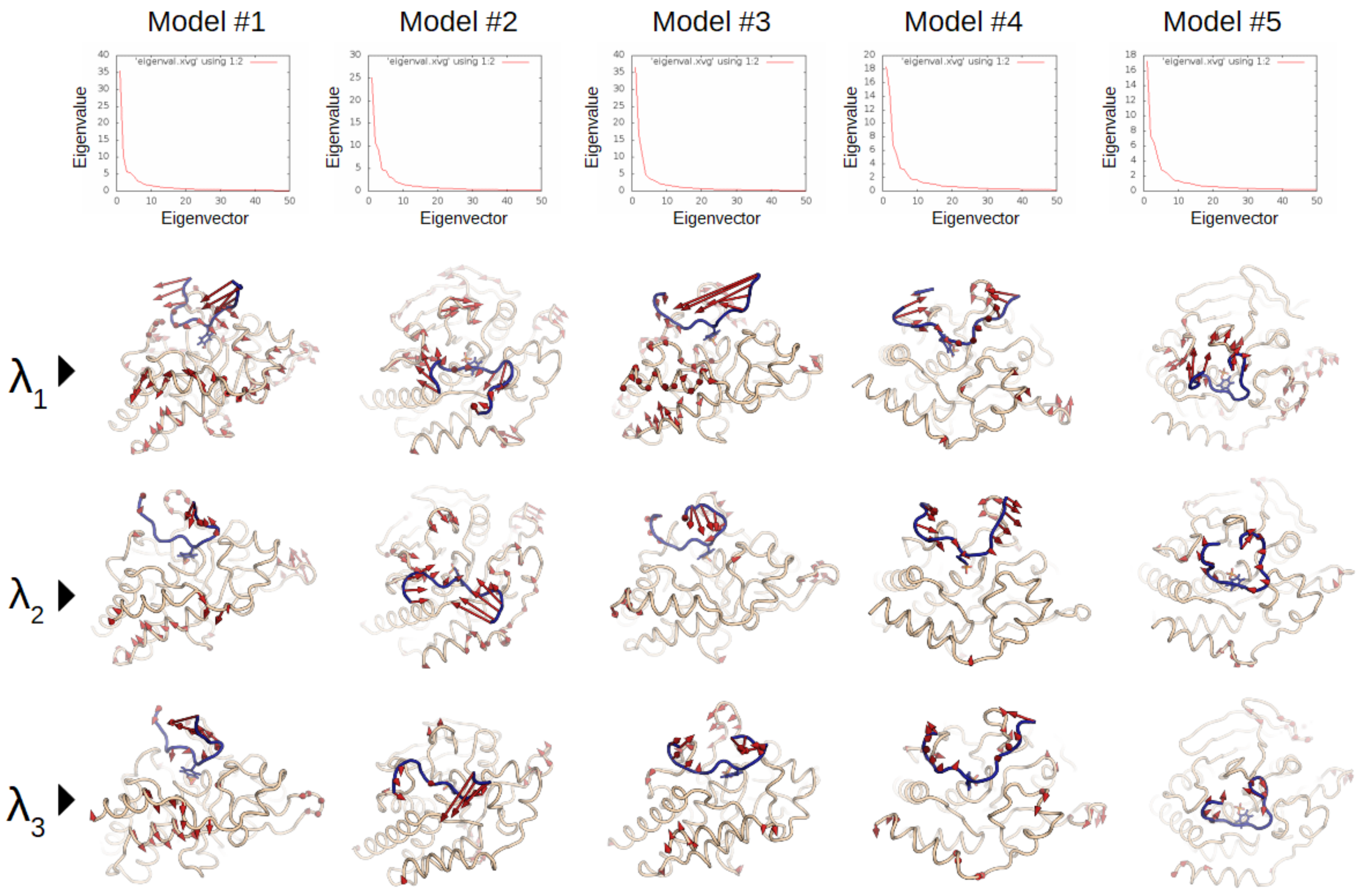

Supplementary figure 77. Principal component analysis for SHP2-p0IRS1 substrate-peptide complex molecular dynamics simulations. As they account for most mobility, only the movements for the 3 largest eigenvalues are shown for each simulation. Vectors were drawn using the “modevectors” PyMol extension.

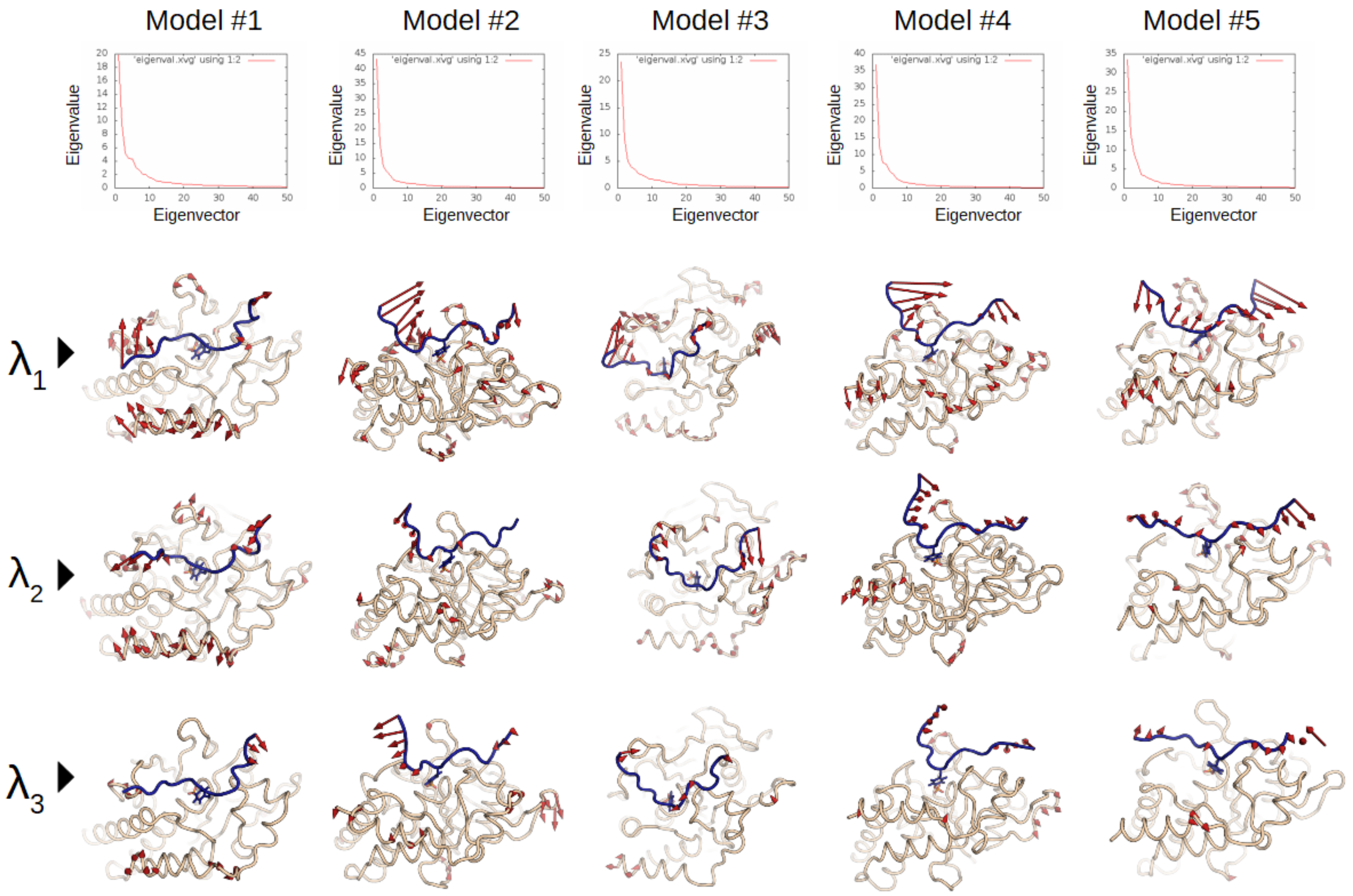

Supplementary figure 78. Principal component analysis for SHP2-p0CD28 substrate-peptide complex molecular dynamics simulations. As they account for most mobility, only the movements for the 3 largest eigenvalues are shown for each simulation. Vectors were drawn using the “modevectors” PyMol extension.

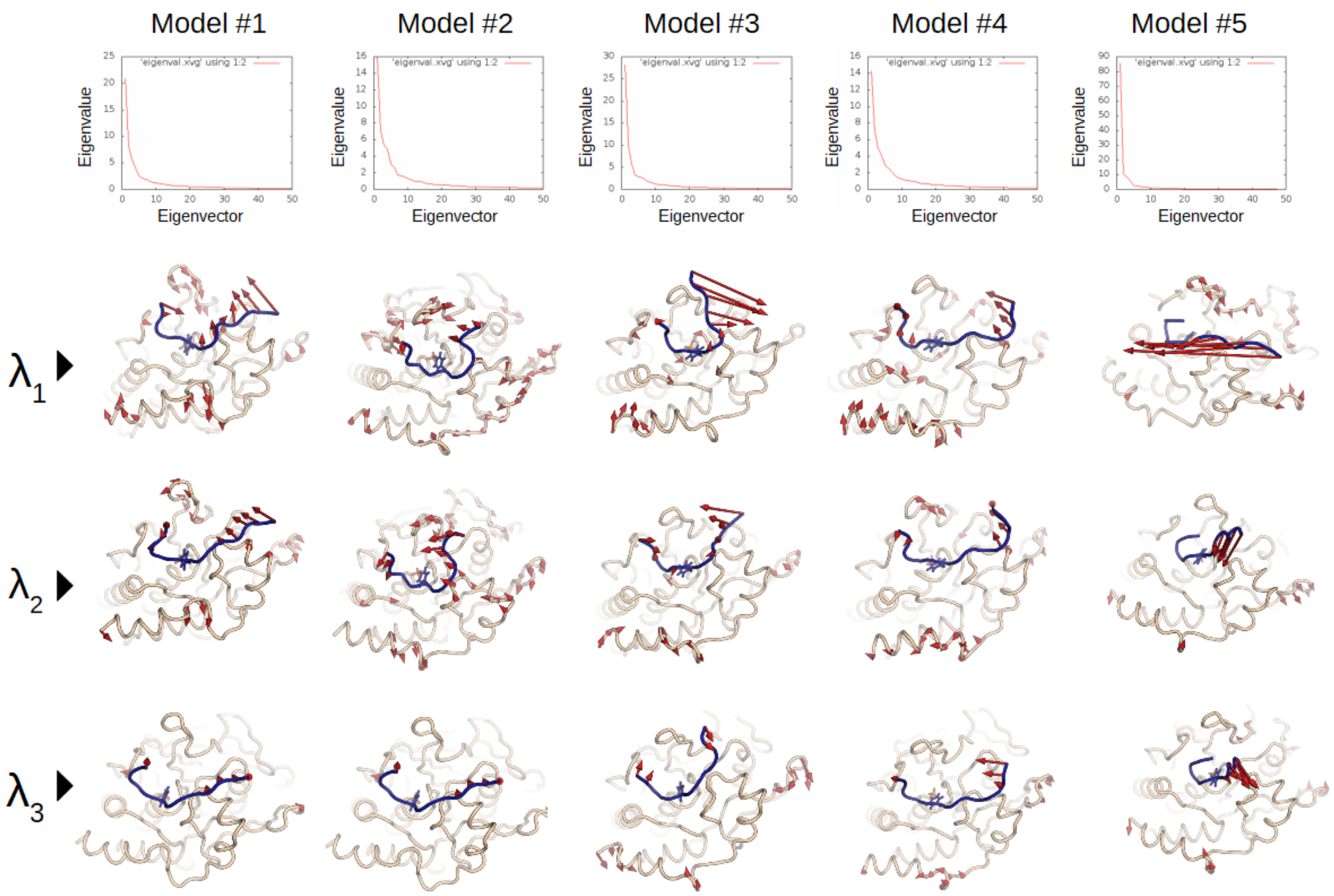

Supplementary figure 79. Principal component analysis for SHP2-p0SRev-IRS1 substrate-peptide complex molecular dynamics simulations. As they account for most mobility, only the movements for the 3 largest eigenvalues are shown for each simulation. Vectors were drawn using the “modevectors” PyMol extension.

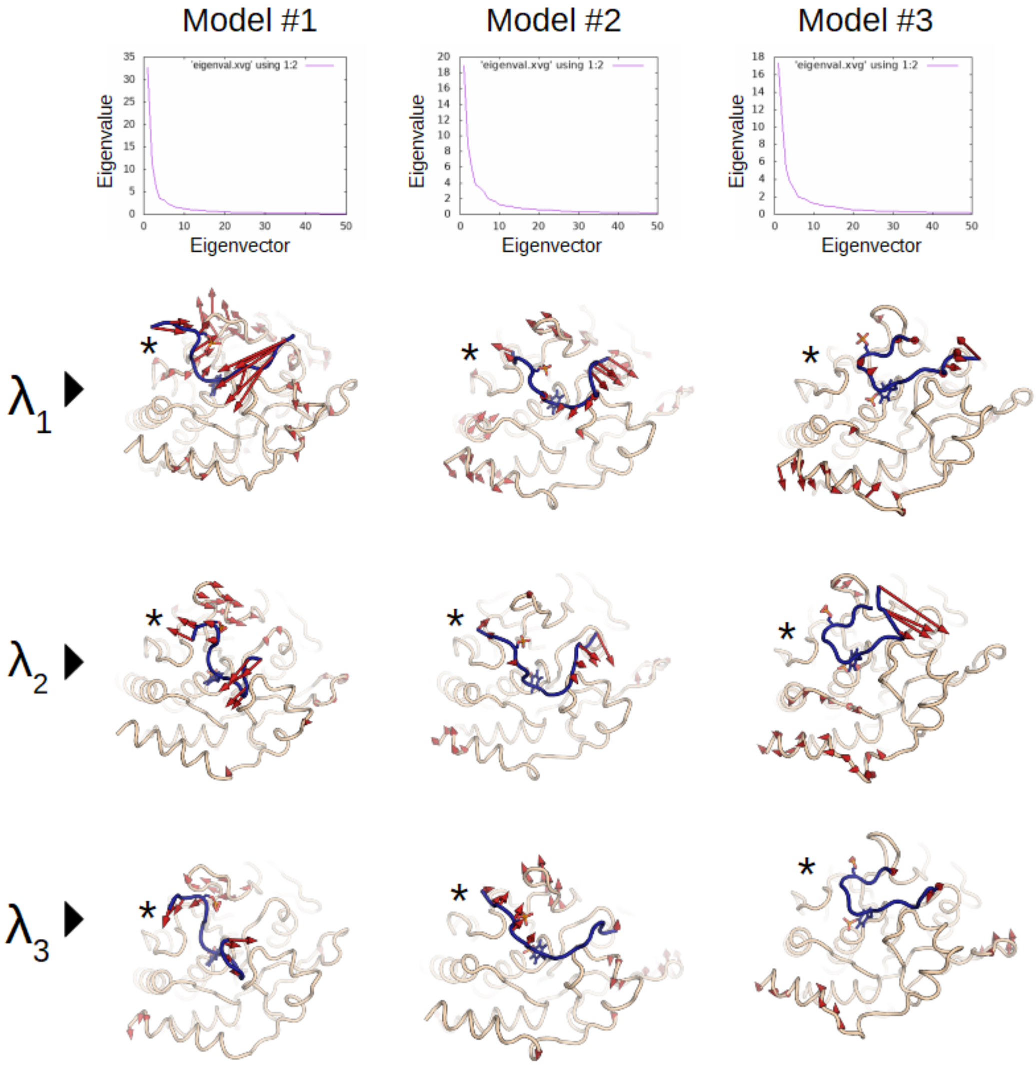

Supplementary figure 80. Principal component analysis of the WPD-closed SHP2-ppIRS1 substrate peptide complex. As they account for most mobility, only the movements for the 3 largest eigenvalues are shown for each simulation. Vectors were drawn using the “modevectors” pymol extension.

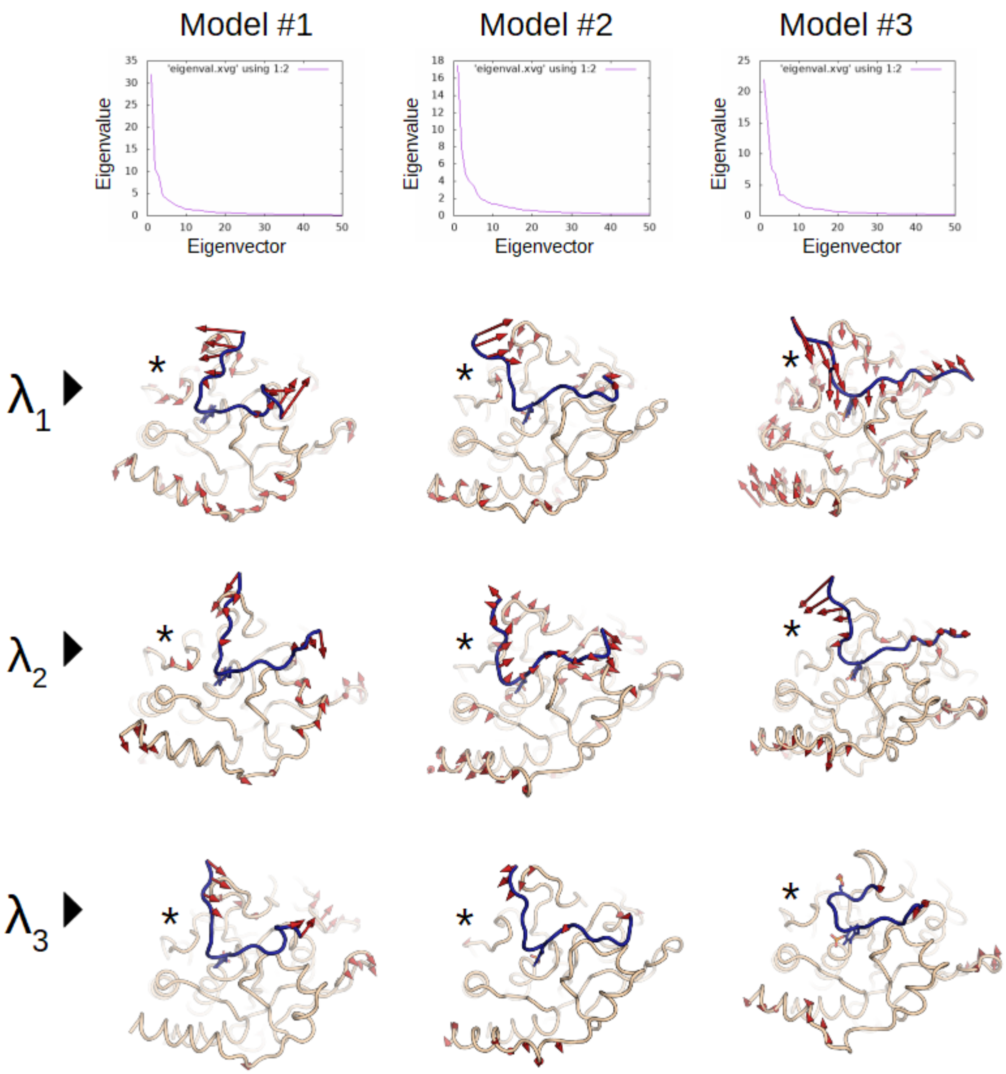

Supplementary figure 81. Principal component analysis of the WPD-closed SHP2-ppCD28 substrate peptide complex. As they account for most mobility, only the movements for the 3 largest eigenvalues are shown for each simulation. Vectors were drawn using the “modevectors” pymol extension.

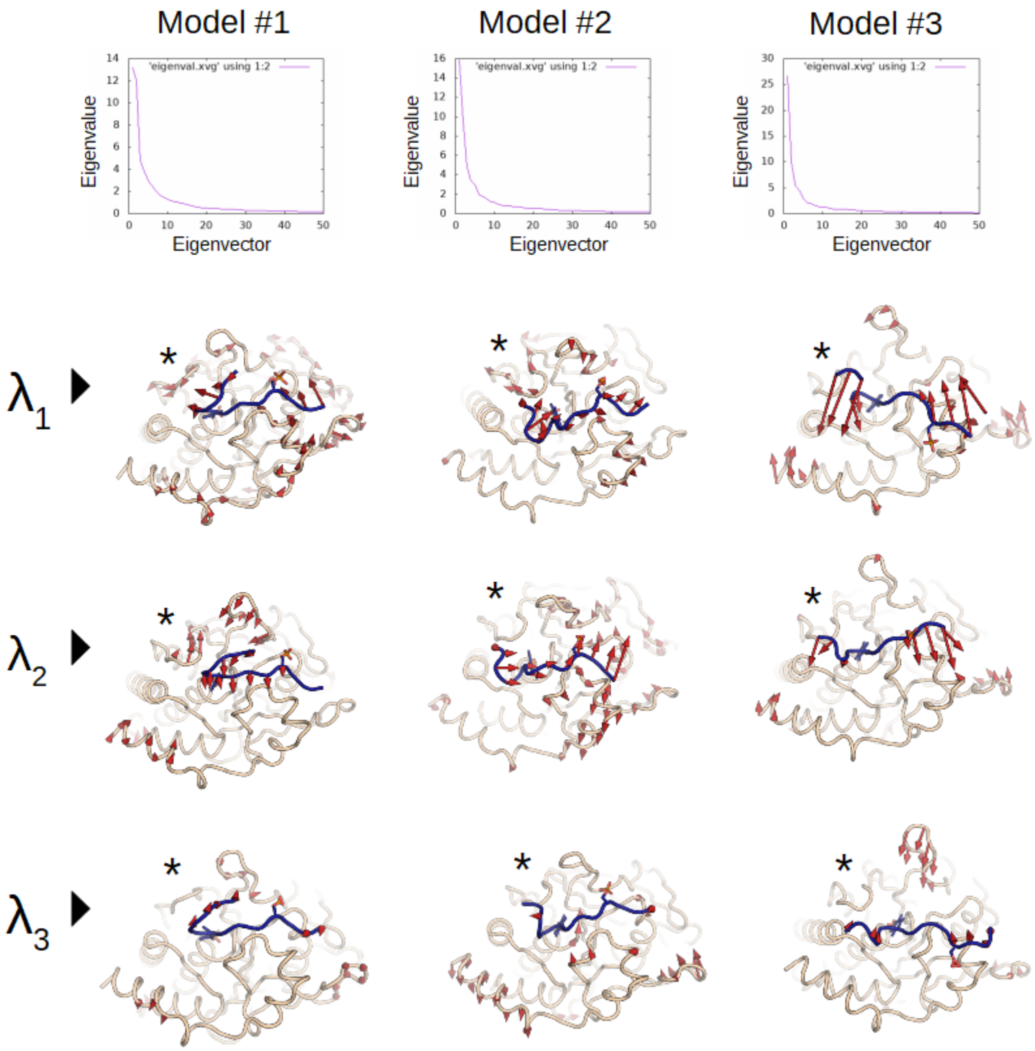

Supplementary figure 82. Principal component analysis of the WPD-closed SHP2-ppSRev-IRS1 substrate peptide complex. As they account for most mobility, only the movements for the 3 largest eigenvalues are shown for each simulation. Vectors were drawn using the “modevectors” pymol extension.

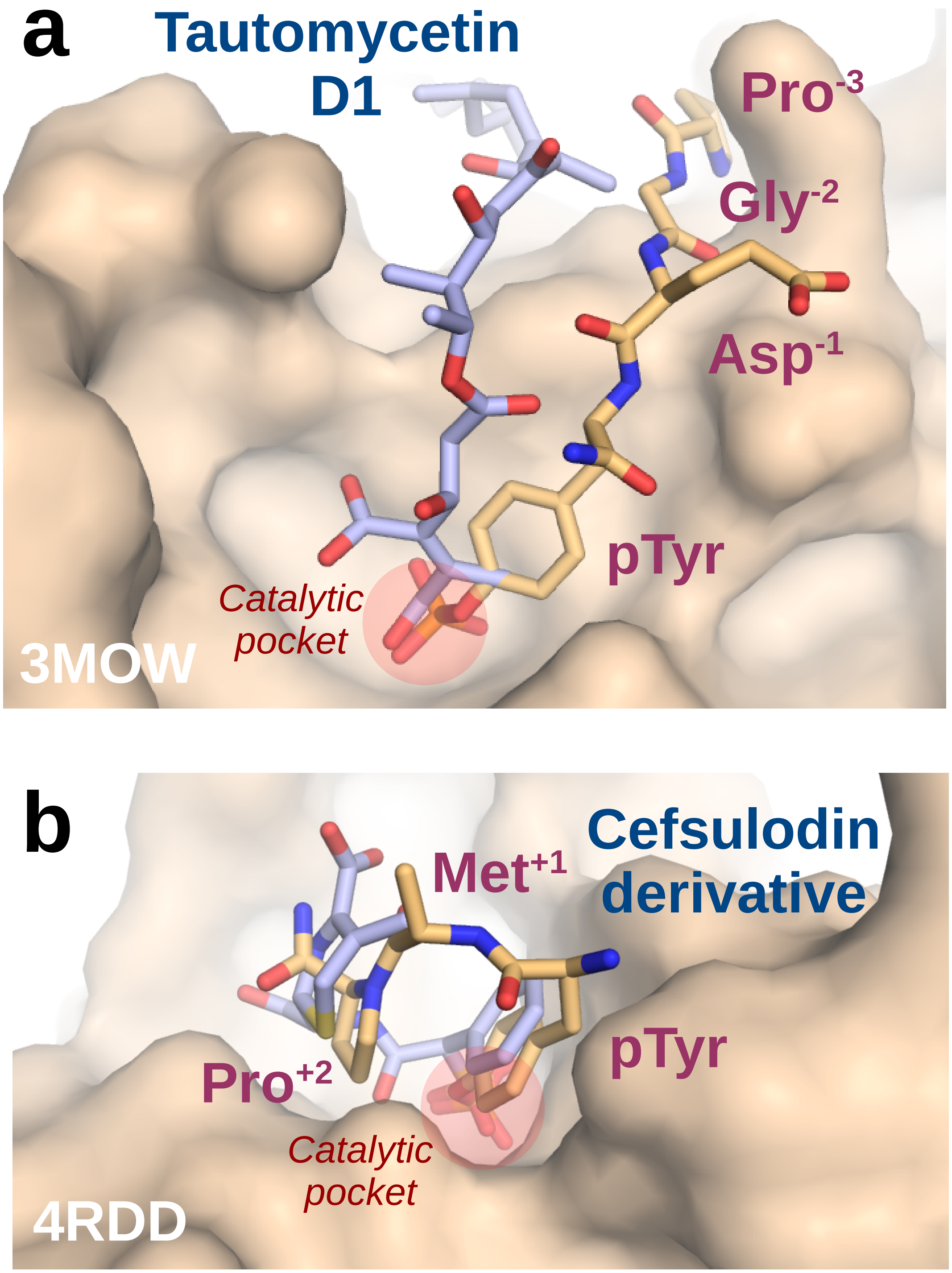

Supplementary figure 83. Crystal structures of orthosteric inhibitors bound to SHP2. Tautomycetin D1 (panel a, above) occupies the position of pTyr and binds very similarly to the preceding peptide chain of ppSRev-IRS1. Cefsulodin and its derivatives (panel b, below) occupy almost exactly the same spot as the pTyr and the +2 Pro in the ppIRS1 peptide.

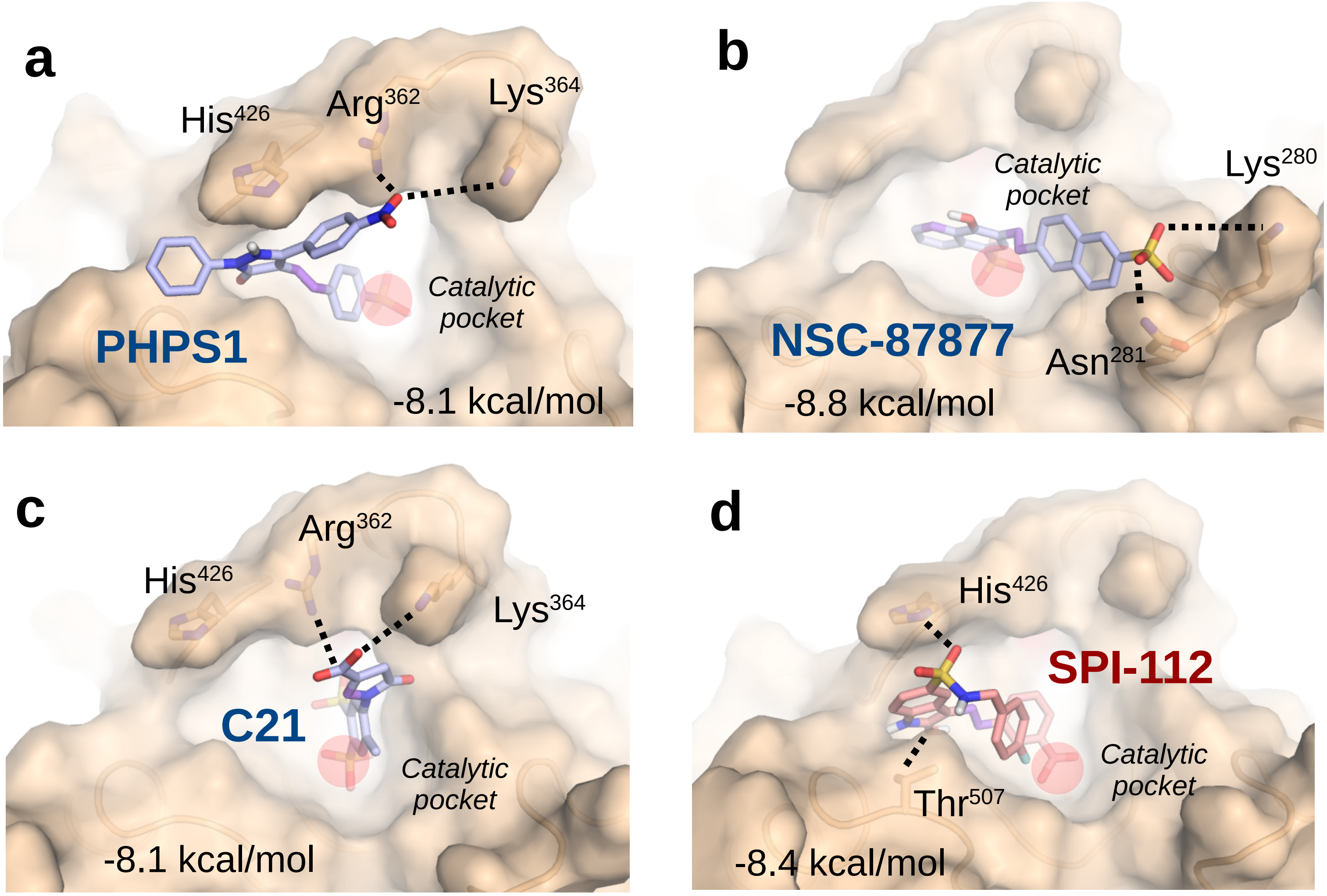

Supplementary figure 84. Most likely poses of orthosteric, substrate-competitive SHP2 inhibitors binding to the open conformation of the enzyme as predicted by AutoDock Vina (from free enthalpies). All inhibitors are known to be competitive, with either sulfonic acid (a: PHPS1, b: NSC-87878 and c: C21, blue) or carboxylic acid (d: SPI-112, red) moieties occupying the catalytic pocket. Potential polar contacts to specific amino acids are indicated by dashed lines.

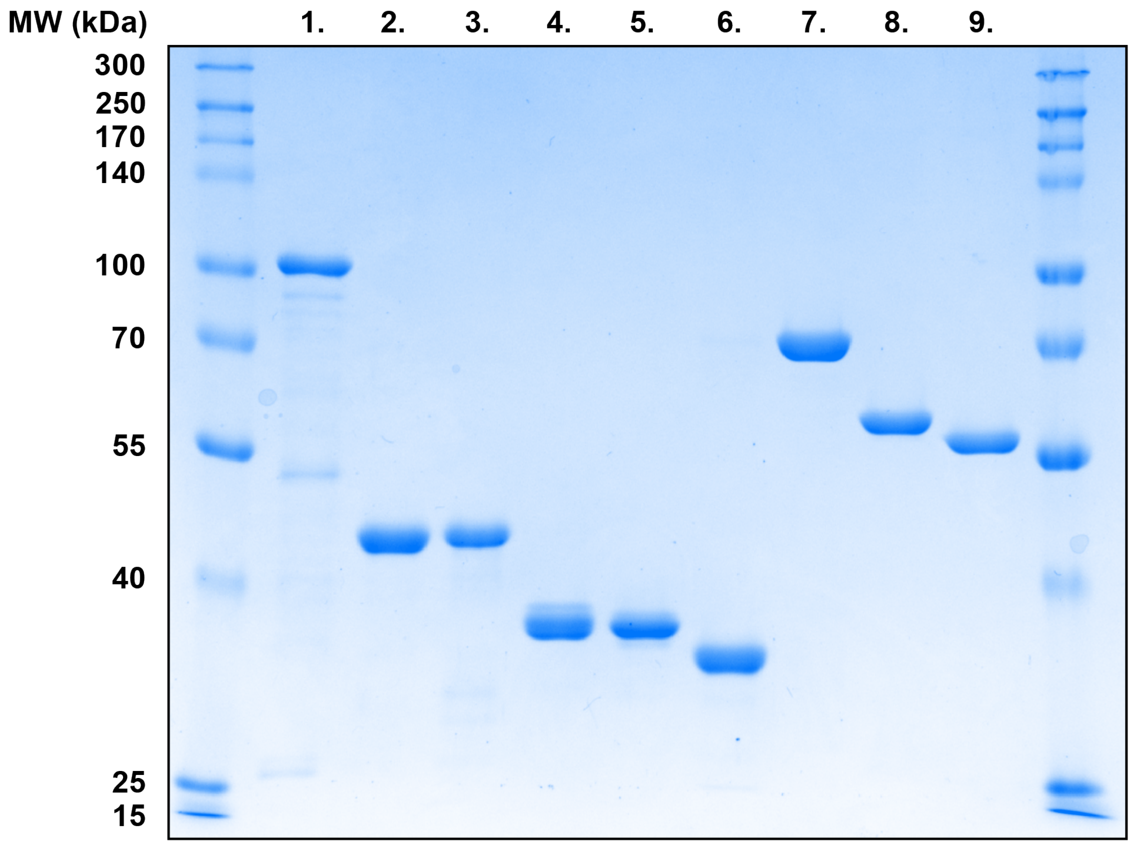

1. **SHP2-full length** (GST-tag; active)
2. **SHP2-cata** (219-528; active)
3. **SHP2-cata C459S** (219-528; inactive)
4. **PTP1B-cata** (1-229; active)
5. **PTP1B-cata C215S** (1-229; inactive)
6. **PTPRε-cata** (107-399; active)
7. **PTPRε-cata C335S** (107-399 + MBP-tag; inactive)
8. **PI3K-cSH3** (PI3KR1 614-724 + MBP-tag)
9. **PI3K-nSH3** (PI3KR1 321-433 + MBP-tag)

Supplementary figure 85. SDS-PAGE quality controls of various purified proteins used in the current study. All protein samples (2ug of each) were loaded on 10% polyacrylamide gel and stained using Coomassie brilliant blue (single gel without repetitions)

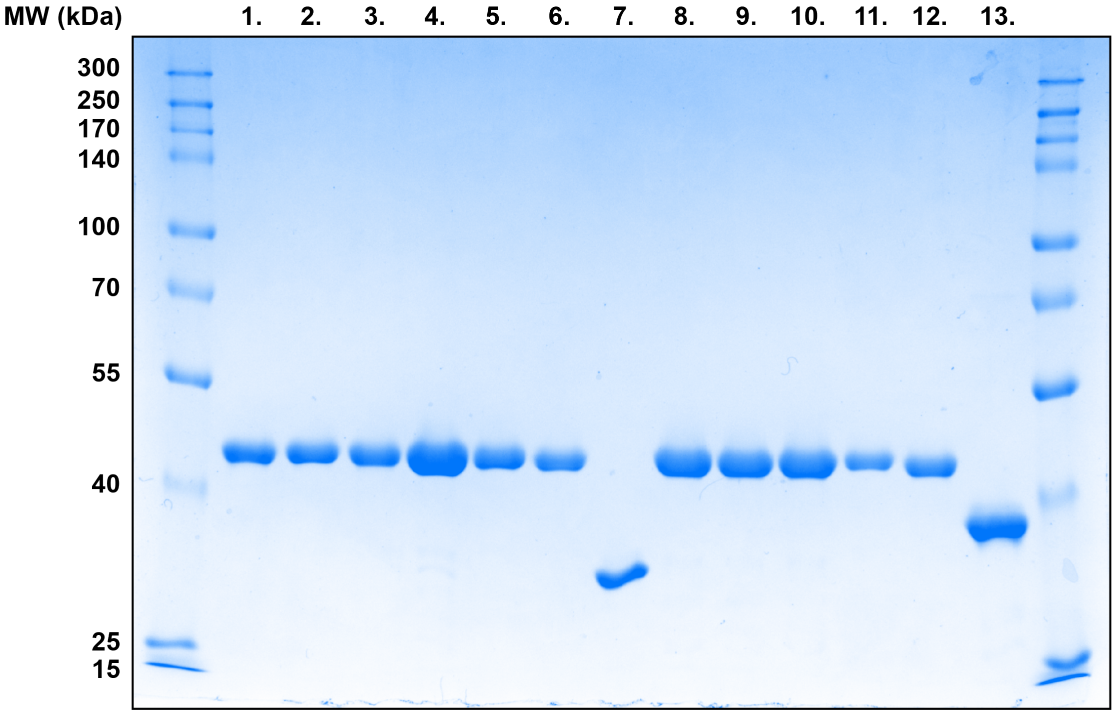

1. **SHP2 K274E** (**Hillmut**; 219-528 + C459S; inactive)
2. **SHP2 K364E** (**Loopmut**; 219-528 + C459S; inactive)
3. **SHP2 R362G+K364S** (**Loopless**; 219-528 + C459S; inactive)
4. **SHP2 R362E** (**Loopinv**; 219-528 + C459S; inactive)
5. **SHP2 K260E+R265S** (**Pocketless**; 219-528 + C459S; inactive)
6. **SHP2 H425F** (**Flapmut**; 219-528 + C459S; inactive)
7. **SHP2 Cryst** (Δ(219-245)-Δ(315-323)+GSSG + C459S; inactive)
8. **SHP2 K364E** (**Loopmut active**; 219-528)
9. **SHP2 R362G+K364S** (**Loopless active**; 219-528)
10. **SHP2 R362E** (**Loopinv active**; 219-528)
11. **SHP2 H426A** (219-528; active)
12. **SHP2 D425A** (219-528; active)
13. **SHP2 Cryst act** (Δ(219-245)-Δ(315-323)+GSSG; active)

Supplementary figure 86. SDS-PAGE quality controls of purified SHP2 mutant proteins used in the current study. All protein samples (2ug of each) were loaded on 10% polyacrylamide gel and stained using Coomassie brilliant blue (single gel without repetitions)

Supplementary tables

Peptide name	Sequence	Activity (Arb. u.)
CADH2-Y785	GGGE**E**DQD**Y**DLSQLQQP	0.7
ERBB2-Y1248	TPTA**E**NPE**Y**LGL**D**VPV-	0.6
PGFRB-Y751	MSKD**E**SVD**Y**VPMLDMKG	1
LCK-Y394	ARLI**E**DNE**Y**TAR**E**GAKF	1.3
CSK-Y184	TVAAQDEF**Y**RSGWALNM	1.5
GHR-Y487	SSLSNIDF**Y**AQVSDITP	0.8
TIE2-Y816	KNNP**D**PTI**Y**PVL**D**WNDI	1.2
ERBB2-Y1139	LTCSPQPE**Y**VNQPDVRP	1.2
WASP-Y290	DAETSKLI**Y**DFI**E**DQGG	0.6
PAXI-Y118	RVGE**E**EHV**Y**SFPNKQKS	0.8

Supplementary table 1. SHP2 substrate sites derived from a large-scale comparative study with quantitative results [1]. A cutoff of > 0.5 was applied to separate genuine SHP2 substrates from spurious signals. Phosphorylated tyrosine residues and potential phosphorylation mimics at +4 or -4 positions are underlined.

Protein (site) name	Substrate sequence	Reference
EGFR-Y992	DDVV**D**ADE**Y**LIPQQGFF	[2]
ERBB2-Y1023	GDLV**D**AEE**Y**LVPQQGFF	[2]
FAK1-Y397	VSVS**E**TDD**Y**AEIIDEDT	[3]
ARHGAP35-Y1105	PRNE**E**ENI**Y**SVPHDSTQ	[4]
OCLN-Y443	NFDTGLQE**Y**KSLQSELD	[4]
PLCG2-Y818	DYGTRIQQ**Y**FPSNYVED	[4]
LYN-Y508	FYTATEGQ**Y**QQQP----	[5]
GAB1-Y317	IPPTPGNT**Y**QIPRTFPE	[6]
JAK2-Y1008	VLPQ**D**KEY**Y**KVK**E**PGES	[7]
SRC-Y530	YFTSTEPQ**Y**QPG**E**NL--	[8]
ROCK2-Y722	RLADKNKI**Y**ESI**E**EAKS	[9]
CDC73-Y290	TKQPIPAA**Y**NRY**D**QERF	[10]
CDC73-Y293	PIPAAYNR**Y**DQERFKGK	[10]
CDC73-Y315	FKIDTMGT**Y**HGMTLKSV	[10]
NMDE2-Y1252	ACKKAGNL**Y**DIS**E**DNSL	[11]
CADH5-Y658	GGEM**D**TTS**Y**DVSVLNSV	[12]
ASK1-Y718	EIPERDSR**Y**SQPLHEEI	[13]
KRAS-Y32	IQNHFVDE**Y**DPTIEDSY	[14]
KRAS-Y64	LDTAGQEE**Y**SAMRDQYM	[14]
SPRY1-Y53	KAIRGSNE**Y**TEGPSVVK	[15]

Supplementary table 2. Biochemically validated direct SHP2 substrate sites derived from low-throughput literature data. Phosphorylated tyrosine residues and potential phosphorylation mimics at +4 or -4 positions are underlined. For individual publications, see supplementary refereces

PDB code	Phos-phatase	Substrate peptide	Dist-01 (P_2-Ala_N)	Dist-02 (P_1-Ile_N)	Dist-03 (P_1-Gly_N)	Dist-04 (P_3-Arg_N)	Dist-05 (T_A-Tyr_B)	Dist-06 (T_A-Ile_G)	Dist-07 (G_O-Lys_N)
1EEN	PTP1B	ADX{pY}LIP	3.0	2.9	2.8	3.0	3.5	3.8	2.8
1EEO	PTP1B	ELEF{pY}MDYE	3.2	3.1	2.9	2.9	3.5	3.8	2.8
4ZRT	PTP1B	GPL{pY}DE	3.4	3.1	2.8	2.9	3.8	3.9	3.2
1LQF	PTP1B	FX{pY}	2.5 †	3.2 †	2.6 †	3.5 †	3.7	3.8	2.8
3ZMP	PTP1B	EPQ{pY}QPGEN	3.6 †	3.2 †	3.5 †	2.5 †	3.6	3.9	4.9 ‡
1G1F	PTP1B	ETD{pY}XR	3.1	2.9	2.9	3.0	3.7	3.9	2.9
1G1H	PTP1B	TD{pY}XRKG	3.3	3.0	2.9	3.0	3.7	4.3	3.7
1G1G	PTP1B	TDY{pY}R	3.0	3.0	2.8	2.9	3.9	4.0	3.0
1YGU	CD45	PT{pY}S	3.2	3.1	2.8	2.6	3.4	5.3	3.3
3D42	HePTP	TE{pY}V	2.9	3.2	2.7	2.8	3.8	4.8	3.6
3D44	HePTP	DE{pY}V	3.0	3.0	2.7	2.7	3.6	4.0	3.2
3OLR	PTPN22	YGEE{pY}DDLY	3.0	2.7	2.6	2.8	3.7	3.5	5.8 ‡
3OMH	PTPN22	DGEE{pY}DDPF	3.2	2.8	3.2	2.7	3.8	3.4	7.1 ‡
4QUM	PTPH1	MXG{pY}VVTR	3.0	3.0	2.6	2.8	3.5	3.9	4.1
4RH5	PTPH1	FSA{pY}PS	3.0	2.9	2.8	2.9	3.9	3.8	2.8
4S0G	PTPH1	FSA{pY}VS	2.9	2.9	2.7	2.8	3.8	3.9	3.0
4RH9	PTPH1	FSA{pY}PSEE	3.0	3.0	2.8	2.9	3.9	3.8	2.8
4RHG	PTPH1	FSA{pY}PSE	2.9	2.9	2.8	2.8	3.8	3.9	2.9
4ICZ	PTPN9	NLX{pY}W	2.9	2.9	2.7	2.9	3.8	3.6	2.9
4GFU	PTPN18	PE{pY}LGLD	3.1	2.7	3.3	3.1	3.5	4.1	3.2
4GFV	PTPN18	PE{pY}LTP	2.9	2.5	3.0	3.0	3.5	3.7	2.9
4NND	PTPN18	LQR{pY}SE	3.2	2.9	3.2	3.1	3.6	3.7	3.0
**Average**	**ALL**	**ALL**	**3.06**	**2.93**	**2.85**	**2.88**	**3.68**	**3.95**	**3.10**
± StDev	ALL	ALL	0.15	0.16	0.19	0.13	0.15	0.41	0.36
CV%	ALL	ALL	*5%*	*6%*	*7%*	*5%*	*4%*	*10%*	*12%*
Our crystal	*SHP2*	*KGSGD{pY}MPM*	*3.1*	*3.2*	*2.9*	*2.9*	*3.8*	*3.7*	*3.3*
Our crystal	*SHP2*	*SD{pY}MN*	*3.0*	*3.1*	*2.8*	*2.8*	*3.9*	*3.8*	*2.9*
Our crystal	*SHP2*	*PGE{pY}VN*	*3.1*	*3.0*	*2.9*	*2.8*	*3.8*	*3.7*	*2.8*
MIN	ALL	ALL	2.9	2.5	2.6	2.6	3.4	3.4	2.8
MAX	ALL	ALL	3.4	3.2	3.3	3.1	3.9	5.3	4.1

Supplementary table 3. Characteristic distances between key atoms in published phosphotyrosine phosphatase and substrate structures (used for HADDOCK minimal restraint definition). Distances are abbreviated as follows: Dist-01 = dist(pTyrO2P-Ala461N), Dist-02 = dist(pTyrO1P-Ile463N), Dist-03 = dist(pTyrO1P-Gly464N), Dist-04 = dist(pTyrO3P-Arg465N), Dist-05 = dist(pTyrCB-38CG), Dist-06 = dist(pTyrCB-Ile282CG1), Dist-07 = dist(GlyO-Lys280N) [-2 aa preceding the pTyr]. The following special cases were omitted from evalution: †Tyrosine phosphonic acid substrate analogues (angles differ from pTyr) and ‡Substrate in divergent conformation lacking H-bonding at the -2 position.

Oligonucleotide name	Sequence
SHP2-LoopInv-Fwd	5′-gtcatgacaacgaaagaagtggaggaaggaaagagtaaatgtgtcaaatactgg-3′
SHP2-LoopInv-Rev	5′-ccagtatttgacacatttactctttccttcctccacttctttcgttgtcatgac-3′
SHP2-ActCys-Fwd	5′-ggccggtcgtggtgcactgcagtgctggaattggccggacag-3′
SHP2-ActCys-Rev	5′-ctgtccggccaattccagcactgcagtgcaccacgaccggcc-3′
SHP2-DA-mut-Fwd	5′-ccactttcggacctggccggcccacggcgtgcccagc-3′
SHP2-DA-mut-Rev	5′-gctgggcacgccgtggGccggccaggtccgaaagtgg-3′
SHP2-HA-mut-Fwd	5′-ccactttcggacctggccggacgccggcgtgcccagcgaccc-3′
SHP2-HA-mut-Rev	5′-gggtcgctgggcacgccggcgtccggccaggtccgaaagtgg-3′
SHP2-trunc	5′-ttttttggatccggtagcggcttttgggaagaatttgagacactac-3′
SHP2-loopedit-Fwd	5′-gcaaatatcatcatgcctgaatttggaagctctggtaaaaagagttacattgccacac-3′
SHP2-loopedit-Rev	5′-gtgtggcaatgtaactctttttaccagagcttccaaattcaggcatgatgatatttgc-3′
SHP2-cata-Fwd	5′-ccccttggatccactcgtataaatgctgctgaaatagaaagc-3′
SHP2-cata-Rev	5′-ttttatgtgcggccgcttacctgcgctgtagtgtttcaatataatgc-3′
SHP2-Loopmut-Fwd	5′-aacgaaagaagtggagagaggagagagtaaatgtgtcaaatactgg-3′
SHP2-Loopmut-Rev	5′-ccagtatttgacacatttactctctcctctctccacttctttcgtt-3′
SHP2-Loopless-Fwd	5′-catgacaacgaaagaagtggagggaggaagtagtaaatgtgtcaaatactggcctg-3′
SHP2-Loopless-Rev	5′-caggccagtatttgacacatttactacttcctccctccacttctttcgttgtcatg-3′
SHP2-Flapmut-Fwd	5′-cggacctggccggacttcggcgtgcccagcg-3′
SHP2-Flapmut-Rev	5′-cgctgggcacgccgaagtccggccaggtccg-3′
SHP2-Hillmut-Fwd	5′-gaaaagagggtcaaaggcaagaaaacgaaaacaaaaatagatataaaaacatcc-3′
SHP2-Hillmut-Rev	5′-ggatgtttttatatctatttttgttttcgttttcttgcctttgaccctcttttc-3′
SHP2-Pocketless-Rev	5′-cttgcctttgaccctcttttgagctgtagagaagttcgcactcctgttgttgtagtgtc-3′
SHP2-Pocketless-Fwd	5′-gacactacaacaacaggagtgcgaacttctctacagctcaaaagagggtcaaaggcaag-3′
PTP1B-mut-Rev	5′-cgatgcctgcactgctgtgcaccacaacggg-3′
PTP1B-mut-Fwd	5′-cccgttgtggtgcacagcagtgcaggcatcg-3′
PTPRe-CS-Rev	5′-ccacgcccgcgctagagtggaccacgatg-3′
PTPRe-CS-Fwd	5′-catcgtggtccactctagcgcgggcgtgg-3′
PTPRe-Fwd	5′-tttaatggatcctgagggcccaagaagtattttcccatcc-3′
PTPRe-NS-Rev	5′-tatttaatgcggccgctgtccccgtagaggtagtactcgagtaagg-3′
PTPRe-Rev	5′-tatttaatgcggccgcttagtccccgtagaggtagtactcgagtaagg-3′
PI3K-cSH2-Fwd	5′-gtggaaggatccgaagatttgccccatcatgatgagaagacatggaatgttgg-3′
PI3K-nSH2-Fwd	5′-gtagttggatccggtatgaataacaatatgtccttacaagatgctgaatgg-3′
PI3K-nSH2-Rev	5′-cttctttggcggccgcttactgttggtatttggatactggataaagtaatttcacatc-3′
IRS1-Mod1-Fwd	5′-ctacccagaggagggtctagaaatgcaccccttggag-3′
IRS1-Mod1-Rev	5′-ctccaaggggtgcatttctagaccctcctctgggtag-3′
IRS1-Mod2-Fwd	5′-cacctccgcctttccacgagctctggtcgccttc-3′
IRS1-Mod2-Rev	5′-gaaggcgaccagagctcgtggaaaggcggaggtg-3′

Supplementary table 4. Oligonucleotides used in the current study for SHP2, PTP1B, PTPRε, PI3KR1 and IRS1 cloning, subcloning and/or mutagenesis purposes

Supplementary notes

Supplementary note 1. Curve fitting of fluorescence polarization (FP) measurements.

To evaluate polarization values from direct and competitive titrations, we used the following fitting formulae in Origin 2018, as detailed below.

Direct titration curves were fitted according to the following formula:

$$I = I_{min} + (I_{max} - {}_{min}) \cdot \frac{T_0 + K_0 + K_d - \sqrt{(T_0 + K_0 + K_d)^2 - 4T_0K_0}}{2K_0} \qquad (1)$$

Where I_{min} is the minimal anisotropy, I_{max} is the (asymptotically estimated) maximal intensity. K_0 is the starting concentration of the protein, while T_0 is the (fixed) concentration of the labelled peptide and K_d is the dissociation constant.

Competitive titration curves were fitted with a different formula (In addition to those defined above, constants K_{dT} K_{dB} B_0 T_0 and D_0 here refer to the Kd derived from the direct titration, the unknown Kd of the analyte, the labelled peptide concentration, the competitor peptide concentration as well as the protein concentration, respectively). The ± sign refers to two different scenarios, depending on the sign of discriminant ($K_{dT} > K_{dB}$ or $K_{dT} < K_{dT}$). For unknown analytes, the choice of sign is not arbitrary, as the fitting will not converge unless the appropriate formula is chosen:

$$I = I_{min} + (I_{max} - {}_{min}) \cdot \left\{ -\frac{\sqrt{A}}{3B_0} \left[\cos\left(\frac{1}{3}\arccos(B)\right) \pm \sqrt{3}\sin\left(\frac{1}{3}\arccos(B)\right)\right] - \frac{C}{3B_0} \right\} \qquad (2)$$

Where A, B and C correspond to the following expressions:

$$A = C^2 - 3B_0D_0 + \frac{3B_0K_{dT}(B_0 + D_0 + K_{dB}) + 3B_0T_0K_{dB}}{K_{dB} - K_{dT}} \qquad (3)$$

$$B = \frac{9C\left[B_0D_0 - \frac{B_0K_{dT}(B_0+D_0+K_{dB}) + B_0T_0K_{dB}}{K_{dB}-K_{dT}}\right] - 2C^3 - 27\frac{B_0^2D_0K_{dT}}{K_{dB}-K_{dT}}}{2\sqrt{-\left\{3B_0D_0 - \frac{3B_0K_{dT}(B_0+D_0+K_{dB})+3B_0T_0K_{dB}}{K_{dB}-K_{dT}} - C^2\right\}^3}} \qquad (4)$$

$$C = \frac{T_0K_{dB} + B_0K_{dT}}{K_{dB} - K_{dT}} - (B_0 + D_0 + K_{dB}) \qquad (5)$$

Supplementary note 2. Simulation of the mean ± SD for Kd ratios.

To estimate the means and errors of the ration distribution on figure 6, we applied a Monte-Carlo approach. The following simulation code was used (wrritten in Python 3) to calculate mean and SD values for the proportion of two Kd (dissociation constant) distributions, assumed as normal:

Ratio-distribution-simulator.py

```
import numpy as np

mu1 = 45.72
sigma1 = 7.49

mu2 = 15.19
sigma2 = 0.49

counts = 5000

dist1 = np.random.normal(mu1, sigma1, counts)
dist2 = np.random.normal(mu2, sigma2, counts)

ratio = []
avrg = []
SD = []

for i, var1 in enumerate(dist1):
      var2 = dist2[i]
      r = var1 / var2
      ratio.append(r)

avrg = np.mean (ratio)
SD = np.std (ratio)

print ("Estimated Mean:", avrg)
print ("Estimated Stdev:", SD)
```

Supplementary note 3. Comparative plotting of characteristic distances observed in molecular dynamics (MD) simulations.

To plot the distances obseved in molecular dynamics (MD) runs, we used simple scripts written in Python 3 utilizing matplotlib (an example is shown below). When the number of runs between models was unequal (as was the case when assessing WPD loop closure), we applied a random sampling procedure to equalize data points. Repeating the same stochastic procedure gave visually highly similar plots.

MD-distance-histogram.py

```
import random
import csv
import numpy
from matplotlib import pyplot

WPD_data = "WPD-SRevIRS1-pp-dist.csv"
orig_data = "WPD-SRevIRS1-orig-dist.csv"

target_column = 1

col1 = []
with open(WPD_data, 'r') as i1:
      reader = csv.reader(i1, delimiter = ';')
      for ind, line in enumerate(reader):
            if ind > 0:
                  data = line[target_column-1]
                  col1.append(float(data))
            else:
                  atom = line[target_column-1]
                  print "Now comparing:", atom

col2_raw = []
with open(orig_data, 'r') as i2:
      reader = csv.reader(i2, delimiter = ';')
      for ind, line in enumerate(reader):
            if ind > 0:
                  data = line[target_column-1]
                  col2_raw.append(float(data))

n = len (col1)
col2 = random.sample(col2_raw, n) # this to equalize data
by random bootsrapping


bins = numpy.linspace(0, 25, 100)

pyplot.hist (col1 , bins, alpha = 0.5, label = 'WPD-closed')
pyplot.hist (col2, bins, alpha = 0.5, label = 'WPD-open')
pyplot.legend (loc = 'upper right')
pyplot.show()
```

Supplementary references

1. Barr AJ et al. Large-scale structural analysis of the classical human protein tyrosine phosphatome. *Cell* **136**, 352-63 (2009)
2. Hartman Z, Geldenhuys WJ, Agazie YM. A specific amino acid context in EGFR and HER2 phosphorylation sites enables selective binding to the active site of Src homology phosphatase 2 (SHP2). *J Biol Chem.* **295**, 3563-3575 (2020)
3. Hartman ZR, Schaller MD, Agazie YM. The tyrosine phosphatase SHP2 regulates focal adhesion kinase to promote EGF-induced lamellipodia persistence and cell migration. *Mol Cancer Res.* **11**, 651-64 (2013)
4. Vemulapalli V et al. Time-resolved phosphoproteomics reveals scaffolding and catalysis-responsive patterns of SHP2-dependent signaling. *Elife* **10** (2021)
5. Sharma N, Everingham S, Ramdas B, Kapur R, Craig AW. SHP2 phosphatase promotes mast cell chemotaxis toward stem cell factor via enhancing activation of the Lyn/Vav/Rac signaling axis. *J Immunol.* **192**, 4859-66 (2014)
6. Montagner A, Yart A, Dance M, Perret B, Salles JP, Raynal P. A novel role for Gab1 and SHP2 in epidermal growth factor-induced Ras activation. *J Biol Chem.* **280**, 5350-60 (2005)
7. Li J et al. Specific dephosphorylation of Janus Kinase 2 by protein tyrosine phosphatases. *Proteomics* **15**, 68-76 (2015)
8. Peng ZY, Cartwright CA. Regulation of the Src tyrosine kinase and Syp tyrosine phosphatase by their cellular association. *Oncogene* **11**, 1955-62 (1995)
9. Lee HH, Chang ZF. Regulation of RhoA-dependent ROCKII activation by Shp2. *J Cell Biol.* **181**, 999-1012 (2008)
10. Takahashi A et al. SHP2 tyrosine phosphatase converts parafibromin/Cdc73 from a tumor suppressor to an oncogenic driver. *Mol Cell.* **43**, 45-56 (2011)
11. Levy AD et al. Noonan Syndrome-Associated SHP2 Dephosphorylates GluN2B to Regulate NMDA Receptor Function. *Cell Rep.* **24**, 1523-1535 (2018)
12. Hatanaka K, Lanahan AA, Murakami M, Simons M. Fibroblast growth factor signaling potentiates VE-cadherin stability at adherens junctions by regulating SHP2. *PLoS One* **7** (2012)
13. Yu L et al. JAK2 and SHP2 reciprocally regulate tyrosine phosphorylation and stability of proapoptotic protein ASK1. *J Biol Chem.* **284**, 13481-13488 (2009)
14. Kano Y et al. Tyrosyl phosphorylation of KRAS stalls GTPase cycle via alteration of switch I and II conformation. *Nat Commun.* **10**, 224 (2019)
15. Takahashi A et al. SHP2 tyrosine phosphatase converts parafibromin/Cdc73 from a tumor suppressor to an oncogenic driver. *Mol Cell.* **43**, 45-56 (2011)
