# Supplementary material for: Structural insights into the pSer/pThr dependent regulation of the SHP2 tyrosine phosphatase in insulin and CD28 signaling
Source: Nat Commun. 2022 Sep 16;13:5439. doi: 10.1038/s41467-022-32918-5 (PMC9481563; doi:10.1038/s41467-022-32918-5)
Supplement: Supplementary file 2 — Description of supplementary datasets [file 41467_2022_32918_MOESM2_ESM.docx]

**Description of supplementary datasets**

**Supplementary dataset 1.**

Representative Haddock ensembles for the ppIRS1, ppCD28 and ppRS-IRS1 complexes

**Supplementary dataset 2.**

PDB files for all molecular dynamics runs (a total of 39)

**Supplementary dataset 3.**

Selected molecular dynamics movies showing the ppIRS1, p0IRS1, ppCD28, p0CD28, ppRS-IRS1 and p0RS-IRS1 peptides onSHP2, as well as representative catalytic-like models.
