# Supplementary material for: Structural insights into the pSer/pThr dependent regulation of the SHP2 tyrosine phosphatase in insulin and CD28 signaling
Source: Nat Commun. 2022 Sep 16;13:5439. doi: 10.1038/s41467-022-32918-5 (PMC9481563; doi:10.1038/s41467-022-32918-5)
Supplement: Supplementary file 6 — Reporting summary [file 41467_2022_32918_MOESM6_ESM.pdf]

nature portfolio

Corresponding author(s): Andras Zeke, MD PhD

Last updated by author(s): Jul 19, 2022

Reporting Summary

Nature Portfolio wishes to improve the reproducibility of the work that we publish. This form provides structure for consistency and transparency in reporting. For further information on Nature Portfolio policies, see our Editorial Policies and the Editorial Policy Checklist.

Statistics

For all statistical analyses, confirm that the following items are present in the figure legend, table legend, main text, or Methods section.

n/a	Confirmed	
☐	☒	The exact sample size (*n*) for each experimental group/condition, given as a discrete number and unit of measurement
☐	☒	A statement on whether measurements were taken from distinct samples or whether the same sample was measured repeatedly
☐	☒	The statistical test(s) used AND whether they are one- or two-sided<br>*Only common tests should be described solely by name; describe more complex techniques in the Methods section.*
☒	☐	A description of all covariates tested
☒	☐	A description of any assumptions or corrections, such as tests of normality and adjustment for multiple comparisons
☐	☒	A full description of the statistical parameters including central tendency (e.g. means) or other basic estimates (e.g. regression coefficient) AND variation (e.g. standard deviation) or associated estimates of uncertainty (e.g. confidence intervals)
☐	☒	For null hypothesis testing, the test statistic (e.g. *F*, *t*, *r*) with confidence intervals, effect sizes, degrees of freedom and *P* value noted<br>*Give P values as exact values whenever suitable.*
☒	☐	For Bayesian analysis, information on the choice of priors and Markov chain Monte Carlo settings
☒	☐	For hierarchical and complex designs, identification of the appropriate level for tests and full reporting of outcomes
☒	☐	Estimates of effect sizes (e.g. Cohen's *d*, Pearson's *r*), indicating how they were calculated

Our web collection on statistics for biologists contains articles on many of the points above.

Software and code

Policy information about availability of computer code

Data collection	Only commercial software was used for data recording: BioTek Gen5 (v3.11) was utilized for fluorescence polarization (FP), an Enspire Multimode Plate Reader (Perkin Elmer Inc.) for small molecule substrate kinetics data collection, and an Agilent Chemstation for CE & CE/MS Systems (ver. B. 04.02. SP1) for evaluation of capillary electrophoresis runs. Western blots were recorded using the Odyssey CLx Fluorescence Imaging System using LICOR v5.2.5. No custom software or script was utilized.
Data analysis	Both commercial and openly available packages were used: XDS (version Jan 26 2018), Aimless (0.6.2), Phaser (3.19), PHENIX (1.17.1-3660) and Coot (0.8.9.2) were utilized for X-ray structure determination. Coot (0.8.9.2) and Pymol (1.8) was used for structural analysis, model building and figure making (in addition to LibreOffice 5.2 for vector graphics). We used the HADDOCK (v2.2) webserver for peptide docking, the Vienna-PTM webserver (2.1) and GROMACS (2018.8) for molecular dynamics and AutoDockTools (v1.5.7) and AutoDock Vina (v.1.1.2) for small molecule inhibitor docking. All kinetics and FP curves were fitted using Origin 2018. Statistics were made using Origin 2018. Molecular dynamics videos were converted to MPEG-4 using OpenShot(v 2.6.1). Only minor custom scripts were utilized, written in Python 3 (included in the appropriate supplementary sections).

For manuscripts utilizing custom algorithms or software that are central to the research but not yet described in published literature, software must be made available to editors and reviewers. We strongly encourage code deposition in a community repository (e.g. GitHub). See the Nature Portfolio guidelines for submitting code & software for further information.

Data

Policy information about availability of data

All manuscripts must include a data availability statement. This statement should provide the following information, where applicable:

- Accession codes, unique identifiers, or web links for publicly available datasets
- A description of any restrictions on data availability
- For clinical datasets or third party data, please ensure that the statement adheres to our policy

Source data supporting the findings of the manuscript has been included in the current article. X-ray structures were deposited to the Protein Data Bank (PDB) under entries 7PPL [http://doi.org/10.2210/pdb7PPL/pdb] (ppIRS1), 7PPN [http://doi.org/10.2210/pdb7PPN/pdb] (ppCD28) and 7PPM [http://doi.org/10.2210/pdb7PPM/pdb] (ppSRev-IRS1), respectively. All other structures analyzed in our article are freely available in the worldwide PDB. Representative Haddock ensembles and MD simulations are also provided as supplementary data (Supplementary datasets 1, 2 and 3). Authors confirm that all other relevant experimental and structural data are included in the article and its supplementary files.

Human research participants

Policy information about studies involving human research participants and Sex and Gender in Research.

Reporting on sex and gender	Not relevant for the current study.
Population characteristics	Not relevant for the current study.
Recruitment	Not relevant for the current study.
Ethics oversight	Not relevant for the current study.

Note that full information on the approval of the study protocol must also be provided in the manuscript.

Field-specific reporting

Please select the one below that is the best fit for your research. If you are not sure, read the appropriate sections before making your selection.

☒ Life sciences ☐ Behavioural & social sciences ☐ Ecological, evolutionary & environmental sciences

For a reference copy of the document with all sections, see nature.com/documents/nr-reporting-summary-flat.pdf

Life sciences study design

All studies must disclose on these points even when the disclosure is negative.

Sample size	No experimental or statistic methods were used that would necessitate a predetermined sample size. In most in vitro experiments with a large effect size (i.e. several fold difference between samples in the measured parameters) and negligible standard deviation, n=3 technical repeats were generally deemed as sufficient. [Data points were only exluded if they could clearly be demonstrated to stem from a technical error.] In the case of cell-based studies (that showed rather small difference and higher variance), we performed paired experiments (always as WT vs. mutant) until the always reproducibly observed differences passed the significance threshold (p0=0.05 and n=12 for baseline IRS1 phosphorylation data). No data point was excluded from the latter studies to yield consistently unbiased results.
Data exclusions	All datasets were included. Individual data points were only exluded if they could clearly be demonstrated to stem from a technical error.
Replication	Separately repeated experiments and/or technical replicates were used, wherever appropriate (as detailed in the Results and Methods sections and relevant figure legends). All replicates supported the conclusions of the article.
Randomization	Randomization and/or oberver blinding is not generally performed or required by strictly in vitro biological studies. It is also not applicable for the determination or modelling of protein structures. Because of the rather large differences (i.e. several fold change) observed between different proteins and peptides, observer bias was generally not an issue. In other words, the outcome of the study was not possible to be altered by unconscious manipulation of data points.
Blinding	Randomization and/or oberver blinding is not generally performed or required by strictly in vitro biological studies. It is also not applicable for the determination or modelling of protein structures. Because of the rather large differences (i.e. several fold change) observed between different proteins and peptides, observer bias was generally not an issue. In other words, the outcome of the study was not possible to be altered by unconscious manipulation of data points.

Reporting for specific materials, systems and methods

We require information from authors about some types of materials, experimental systems and methods used in many studies. Here, indicate whether each material, system or method listed is relevant to your study. If you are not sure if a list item applies to your research, read the appropriate section before selecting a response.

Materials & experimental systems

n/a	Involved in the study
☐	☒ Antibodies
☐	☒ Eukaryotic cell lines
☒	☐ Palaeontology and archaeology
☒	☐ Animals and other organisms
☒	☐ Clinical data
☒	☐ Dual use research of concern

Methods

n/a	Involved in the study
☒	☐ ChIP-seq
☒	☐ Flow cytometry
☒	☐ MRI-based neuroimaging

Antibodies

Antibodies used	M2 anti-FLAG (#1804-200UG Sigma) Lot#: SlCF4933<br>Anti-pY612-iRS1 (#44-816G Invitrogen) Lot#: 2066357<br>IRDye anti-mouse 680 (#92668070 LI-COR Biosciences, Lincoln, NE, USA) Lot#: C90910-21<br>IRDye anti-rabbit 800 (#92632211 LI-COR Biosciences) Lot#: C90723-19
Validation	The antibodies were not tested in-house for specificity directly, e.g. by using mutant proteins. However, the anti-FLAG and LICOR secondary antibodies have been previously found to be robust and reliable while the pY612 IRS1 antibody was selected specifically for this study based on the documentation and validation by the manufacturer (See for example: PMID:31042800, PMID:26100015 or PMID:25383313.). For all other validation regarding these commercially available antibodies, see the manufacturers' website.

Eukaryotic cell lines

Policy information about cell lines and Sex and Gender in Research

Cell line source(s)	HEK293T (#CRL-3216 ATCC, Manassas VA, USA). See the Certificate of Analysis attached separately.
Authentication	Cell line was obtained from a trusted source thus not authenticated locally.
Mycoplasma contamination	HEK293T cells were obtained directly from ATCC as a verified mycoplasma-free cell line product, therefore the cells were not tested directly after arrival. However, regular mycoplasma contamination tests were performed on cultures using MycoAlertTM PLUS Mycoplasma Detection Kit (Lonza, Catalog #: LT07-701). All tests were negative.
Commonly misidentified lines<br>(See ICLAC register)	No commonly misidentified cell lines were used.
